# Template-assisted covalent modification underlies activity of covalent molecular glues

**DOI:** 10.1038/s41589-024-01668-4

**Published:** 2024-07-29

**Authors:** Yen-Der Li, Michelle W. Ma, Muhammad Murtaza Hassan, Moritz Hunkeler, Mingxing Teng, Kedar Puvar, Justine C. Rutter, Ryan J. Lumpkin, Brittany Sandoval, Cyrus Y. Jin, Anna M. Schmoker, Scott B. Ficarro, Hakyung Cheong, Rebecca J. Metivier, Michelle Y. Wang, Shawn Xu, Woong Sub Byun, Brian J. Groendyke, Inchul You, Logan H. Sigua, Isidoro Tavares, Charles Zou, Jonathan M. Tsai, Paul M. C. Park, Hojong Yoon, Felix C. Majewski, Haniya T. Sperling, Jarrod A. Marto, Jun Qi, Radosław P. Nowak, Katherine A. Donovan, Mikołaj Słabicki, Nathanael S. Gray, Eric S. Fischer, Benjamin L. Ebert

**Affiliations:** 1 Department of Molecular and Cellular Biology, Harvard University, Cambridge, MA USA; 2 Department of Medical Oncology, Dana-Farber Cancer Institute, Boston, MA USA; 3 Cancer Program, Broad Institute of MIT and Harvard, Cambridge, MA USA; 4 Department of Cancer Biology, Dana-Farber Cancer Institute, Boston, MA USA; 5 Department of Biological Chemistry and Molecular Pharmacology, Harvard Medical School, Boston, MA USA; 6 Department of Chemical and Systems Biology, Chem-H and Stanford Cancer Institute, Stanford School of Medicine, Stanford University, Stanford, CA USA; 7 Center for Drug Discovery, Department of Pathology & Immunology, and Verna and Marrs McLean Department of Biochemistry and Molecular Pharmacology, Baylor College of Medicine, Houston, TX USA; 8 Blais Proteomics Center and Center for Emergent Drug Targets, Dana-Farber Cancer Institute, Boston, MA USA; 9 Department of Pathology, Brigham and Women’s Hospital, Boston, MA USA; 10 Howard Hughes Medical Institute, Boston, MA USA

**Keywords:** Small molecules, Structural biology, Medicinal chemistry, Mechanism of action

## Abstract

Molecular glues are proximity-inducing small molecules that have emerged as an attractive therapeutic approach. However, developing molecular glues remains challenging, requiring innovative mechanistic strategies to stabilize neoprotein interfaces and expedite discovery. Here we unveil a *trans*-labeling covalent molecular glue mechanism, termed ‘template-assisted covalent modification’. We identified a new series of BRD4 molecular glue degraders that recruit CUL4^DCAF16^ ligase to the second bromodomain of BRD4 (BRD4_BD2_). Through comprehensive biochemical, structural and mutagenesis analyses, we elucidated how pre-existing structural complementarity between DCAF16 and BRD4_BD2_ serves as a template to optimally orient the degrader for covalent modification of DCAF16_Cys58_. This process stabilizes the formation of BRD4–degrader–DCAF16 ternary complex and facilitates BRD4 degradation. Supporting generalizability, we found that a subset of degraders also induces GAK–BRD4_BD2_ interaction through *trans*-labeling of GAK. Together, our work establishes ‘template-assisted covalent modification’ as a mechanism for covalent molecular glues, which opens a new path to proximity-driven pharmacology.

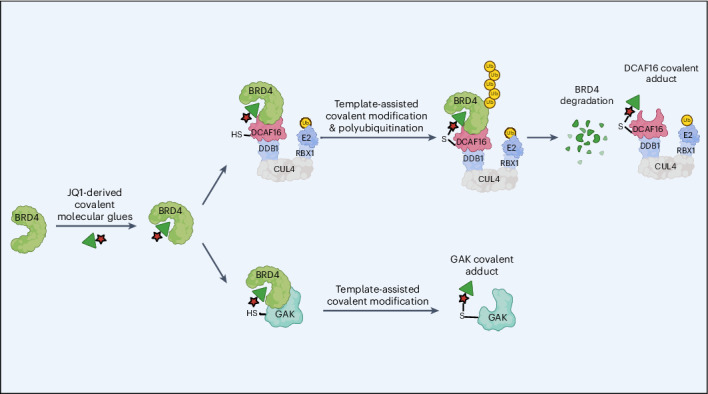

## Abstract (web summary)

Characterization of DCAF16-based BRD4 molecular glue degraders revealed a *trans*-labeling mechanism termed ‘template-assisted covalent modification’, which opens a new path for proximity-driven pharmacology.

## Main

Molecular glue degraders have emerged as a powerful therapeutic modality, as demonstrated by the clinical successes of thalidomide analogs in the treatment of hematological malignancies^1,2^. These small-molecule degraders stabilize the protein–protein interface between ubiquitin ligases and disease-relevant neosubstrates, resulting in ubiquitination and proteasomal degradation of the targets^3^. Unlike traditional occupancy-driven pharmacology of inhibitors, the event-driven pharmacology of degraders can result in more potent and sustained drug activity^4^. The elimination of target proteins by molecular glue degraders decreases both enzymatic and scaffold function of target proteins, leading to differentiated pharmacology and often superior inhibition of protein function^5^. Moreover, molecular glue degraders hold the potential to target proteins that do not have ligandable pockets and are considered difficult to drug, including transcription factors^6^.

The clinical efficacy of thalidomide-derived drugs, such as lenalidomide, and the broad utility of targeted protein degradation in research and drug discovery have inspired numerous efforts to explore proximity-driven pharmacology^7–9^. Although bifunctional molecules, such as PROTACs, can lead to rapid proof of concept and highly potent chemical probes, molecular glues are favorable for clinical development due to reduced size and overall chemical properties^3,6^. Despite these advantages, to date, only a small number of ubiquitin ligases have been exploited by molecular glue degraders, including CRBN^10,11^, DCAF15 (ref. ^12^) and DDB1 (refs. ^13–15^). Other proximity-driven approaches lack molecular glues. Covalency has the potential not only to aid the discovery of molecular glues but also to impart improved efficacy through strengthening of the interface^16,17^. Chemo-proteomic studies have indeed identified putative covalent molecular glues^18,19^, but it remains to be shown mechanistically whether these molecules truly act as molecular glues and whether general principles can be derived to aid future discovery.

Here we demonstrate that a set of derivatives of JQ1 (ref. ^20^), a non-degrading inhibitor of BRD4, act as molecular glue degraders. Using genetic screens, biochemical analyses, medicinal chemistry, structural studies and systematic mutagenesis, we elucidate the mechanism of action for a novel class of degraders that act through template-assisted covalent modification of DCAF16. We also demonstrate this *trans*-labeling mechanism in the context of the GAK–BRD4 interaction facilitated by reactive JQ1 analogs, indicating the generality of this mechanism.

## Results

### JQ1-derived compounds degrade BRD4 via DCAF16

**Fig. 1:**
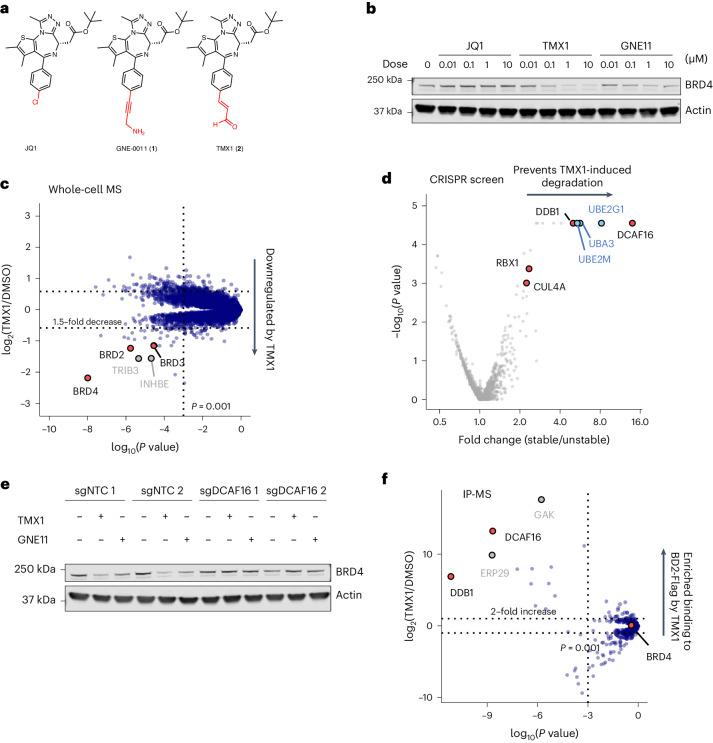
JQ1-derived compounds degrade BRD4 via DCAF16. **a**, Chemical structures of JQ1, GNE11 and TMX1. **b**, Western blots of BRD4 degradation in K562 cells treated with DMSO or different concentrations of JQ1, TMX1 and GNE11 for 16 h. **c**, Quantitative whole proteome analysis of K562 cells after treatment with TMX1 at 0.5 μM (*n* = 2) or DMSO (*n* = 3) for 5 h. Statistical analysis was performed using a two-sided moderated *t*-test as implemented in the limma package. **d**, UPS-focused CRISPR degradation screen for BRD4_BD2_-eGFP stability in K562–Cas9 cells treated with TMX1 at 1 μM for 16 h (*n* = 2). Statistical analysis was performed using a two-sided empirical rank-sum test. **e**, Western blots of BRD4 degradation in DCAF16 and non-targeting control (NTC) sgRNA-infected K562–Cas9 cells treated with DMSO, TMX1 at 1 μM or GNE11 at 1 μM for 16 h. **f**, Flag IP followed by mass spectrometry in 293T cells overexpressing BRD4_BD2_-Flag of cells treated with either MLN4924 plus TMX1 both at 1 μM (*n* = 4) or MLN4924 at 1 μM only (*n* = 4). Fold enrichment and *P* values were calculated by comparing TMX1/MLN4924-treated samples to MLN4924-only control samples. Statistical analysis was performed using a two-sided moderated *t*-test as implemented in the limma package. All western blot data are representative of two independent measurements. Source data

**Extended Data Fig. 1:**
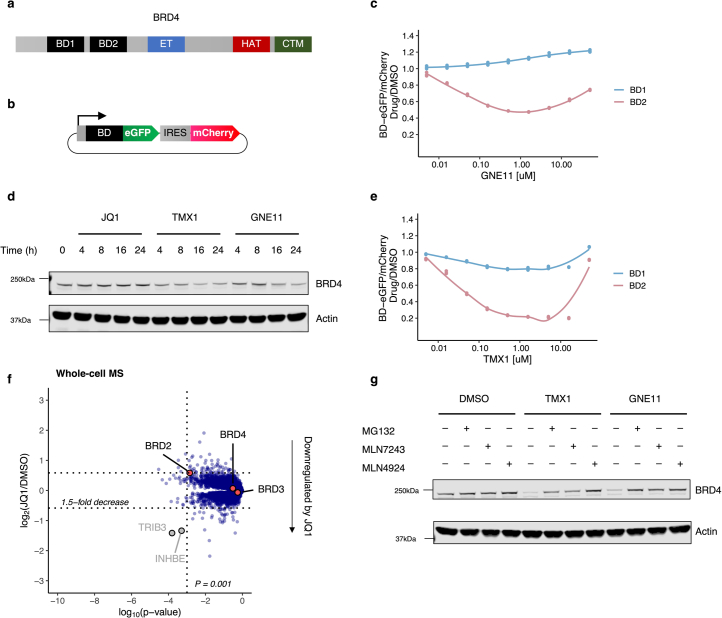
Degradation characterization of JQ1-derived compounds. **a**. The domain structure of BRD4. **b**. Schematic of BRD4_BD_ stability reporter. IRES, internal ribosome entry site. **c**. Flow cytometry analysis of BRD4_BD1_-eGFP and BRD4_BD2_-eGFP degradation in K562 cells that were treated with increasing concentrations of GNE11 for 16 h (n = 3). **d**. Western blots of BRD4 degradation in K562 cells that were treated with JQ1, TMX1 or GNE11 at 1 μM for increasing time points. **e**. Flow cytometry analysis of BRD4_BD1_-eGFP and BRD4_BD2_-eGFP degradation in K562 cells that were treated with increasing concentrations of TMX1 for 16 h (n = 3). **f**. Quantitative whole proteome analysis of K562 cells after treatment with JQ1 at 0.5 μM (n = 1) or DMSO (n = 3) for 5 h. Statistical analysis was performed using a two-sided moderated t-test as implemented in the limma package. **g**. Western blots of BRD4 degradation in K562 cells that were treated with DMSO, TMX1 at 1 μM, GNE11 at 1 μM, MG132 at 10 μM, MLN7243 at 1 μM, and MLN4924 at 1 μM for 16 h. All western blot data are representative of two independent measurements. Source data

GNE-0011 (GNE11) (**1**) is a derivative of the inhibitor JQ1 that has been reported to degrade BRD4 (refs. ^21,22^). To characterize the activity of GNE11, we generated and optimized a fluorescent reporter assay for BRD4 stability (Extended Data Fig. 1a,b). We found that GNE11 induces selective degradation of a fluorescent reporter containing the second bromodomain of BRD4 (BRD4_BD2_) with a maximal depth of degradation at 16 h (D_max/16 h_) of approximately 50% (Extended Data Fig. 1c), indicating that the BRD4_BD2_ domain, but not the first bromodomain of BRD4 (BRD4_BD1_) domain, serves as the degron for drug-mediated degradation. Through synthesis of a series of GNE11 structural analogs, we discovered an acrolein analog, TMX1 (**2**), that exhibited more potent degradation of BRD4 (Fig. 1a,b and Extended Data Fig. 1d) while maintaining selectivity for BRD4_BD2_ (D_max/16 h_ ~ 80%) (Extended Data Fig. 1e). To examine the specificity of TMX1, we performed quantitative proteome-wide mass spectrometry in K562 cells after treatment with TMX1 for 5 h. BRD4 was the primary degradation target with a more minor effect on two of the other BET family proteins, BRD2 and BRD3 (Fig. 1c). Treatment with JQ1, which lacks the acrolein moiety of TMX1, did not alter the abundance of BRD2, BRD3 or BRD4 (Extended Data Fig. 1f). In accordance with a ubiquitin-mediated mechanism, BRD4 degradation induced by either TMX1 or GNE11 was rescued by inhibition of the proteasome with MG132, inhibition of the ubiquitin-activating enzyme UBA1 with MLN7243 or inhibition of cullin neddylation with MLN4924 (Extended Data Fig. 1g).

**Extended Data Fig. 2:**
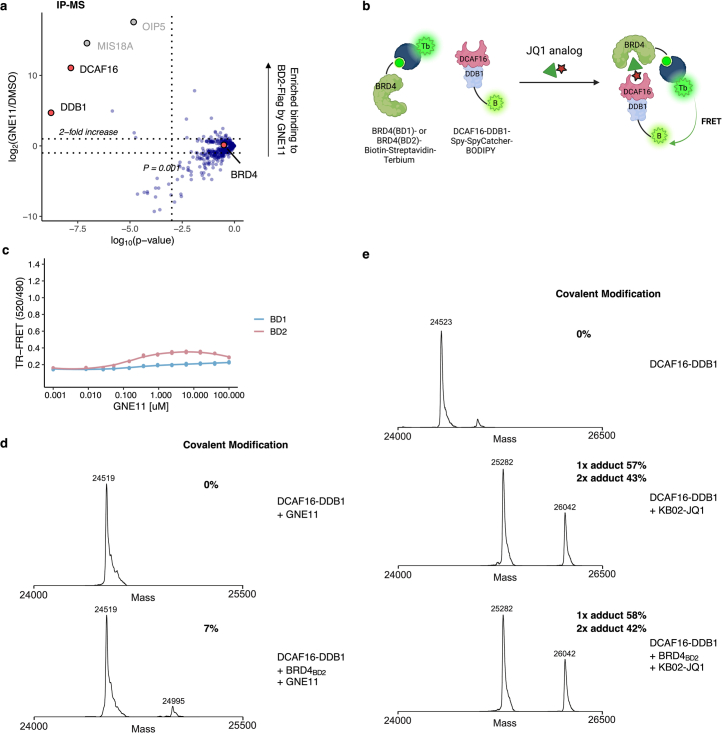
Covalent recruitment of DCAF16 is facilitated by BRD4_BD2_. **a**. Flag immunoprecipitation (IP) followed by mass spectrometry in 293 T cells overexpressing BRD4_BD2_-Flag of cells treated with either MLN4924 plus GNE11 both at 1 μM (n = 4), or MLN4924 at 1 μM only (n = 4). Fold enrichment and p-values were calculated by comparing GNE11/MLN4924 treated samples to MLN4924 only control samples. Statistical analysis was performed using a two-sided moderated t-test as implemented in the limma package. **b**. Schematic of the TR-FRET set-up. Positions of FRET donor (terbium-coupled streptavidin) and acceptor (BODIPY–SpyCatcher) are indicated in the structural model. **c**. TR-FRET signal for DDB1-DCAF16-BODIPY to BRD4_BD1_-terbium or BRD4_BD2_-terbium with increasing concentrations of GNE11 (n = 3). **d**. Intact protein mass spectra of DDB1-DCAF16 co-incubated with GNE11 at 25 °C for 16 h, or DDB1-DCAF16 co-incubated with GNE11 and BRD4_BD2_ at 25 °C for 16 h. **e**. Intact protein mass spectra of DDB1-DCAF16 alone, DDB1-DCAF16 co-incubated with KB02-JQ1 at 4 °C for 16 h, or DDB1-DCAF16 co-incubated with KB02-JQ1 and BRD4_BD2_ at 4 °C for 16 h. Source data

To identify the molecular machinery required for TMX1-mediated and GNE11-mediated BRD4 degradation, we performed CRISPR–Cas9 reporter degradation screens. K562 or 293T cells expressing Cas9 and the BRD4_BD2_ reporter were transduced with a single guide RNA (sgRNA) library targeting genes in the ubiquitin–proteasome system (UPS)^13^ and then sorted for cells with increased and decreased levels of BRD4_BD2_-eGFP after drug treatment (Supplementary Fig. 1a). The screen revealed that TMX1-induced or GNE11-induced reporter degradation requires DCAF16, DDB1, RBX1 and CUL4A (Fig. 1d and Supplementary Fig. 1b–d). In engineered K562 cells with complete genetic knockout of DCAF16 (see Supplementary Note: Deep sequencing results for DCAF16 knockout clones), treatment with TMX1 or GNE11 did not cause BRD4 degradation (Fig. 1e). To corroborate these findings, we performed a CRISPR–Cas9 resistance screen to identify genes required for TMX1-induced and GNE11-induced cellular toxicity (Supplementary Fig. 2a). sgRNAs against DCAF16 were again the most enriched, suggesting that loss of DCAF16 caused TMX1 or GNE11 resistance (Supplementary Fig. 2b,c), whereas DCAF16 was not required for JQ1-induced cellular toxicity (Supplementary Fig. 2d). We validated that sgRNAs targeting DCAF16 confer resistance to the degraders in a competitive growth assay (Supplementary Fig. 2e). Consistent with cellular and genetic data, immunoprecipitation mass spectrometry (IP-MS) experiments with BRD4_BD2_ as a bait confirm a direct and specific compound-dependent interaction with DCAF16 (Fig. 1f and Extended Data Fig. 2a). Collectively, these data indicate that TMX1 and GNE11 act through RBX1–CUL4–DDB1–DCAF16 (CRL4^DCAF16^) ubiquitin ligase-dependent degradation of BRD4.

### Covalent recruitment of DCAF16 is facilitated by BRD4_BD2_

**Fig. 2:**
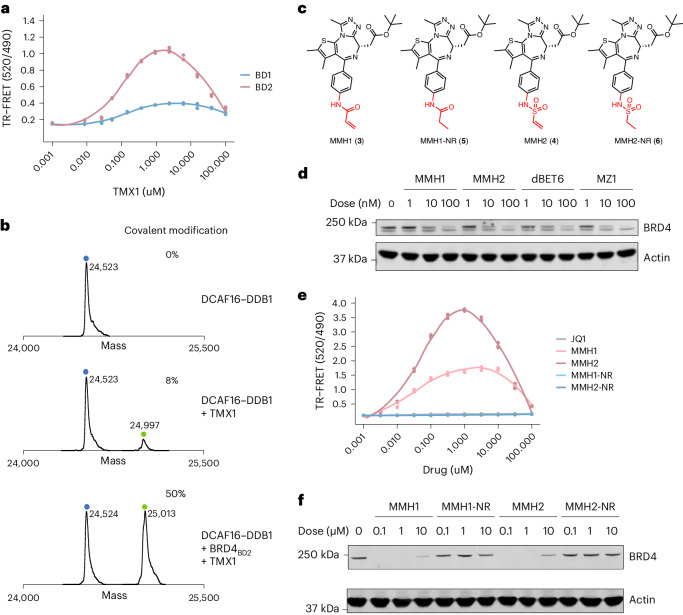
Template-assisted covalent modification of DCAF16 and degraders with optimized electrophilic warheads. **a**, TR-FRET signal for DDB1–DCAF16–BODIPY to BRD4_BD1_-terbium or BRD4_BD2_-terbium with increasing concentrations of TMX1 (*n* = 3). **b**, Intact protein mass spectra of DDB1–DCAF16 alone, DDB1–DCAF16 co-incubated with TMX1 at 4 °C for 16 h or DDB1–DCAF16 co-incubated with TMX1 and BRD4_BD2_ at 4 °C for 16 h. **c**, Chemical structures of MMH1, MMH2, MMH1-NR and MMH2-NR. **d**, Western blot of BRD4 degradation in K562 cells that were treated with DMSO or different concentrations of MMH1, MMH2, dBET6 or MZ1 for 6 h. **e**, TR-FRET signal for DDB1–DCAF16–BODIPY to BRD4_BD2_-terbium with increasing concentrations of JQ1, MMH1, MMH2, MMH1-NR and MMH2-NR (*n* = 3). **f**, Western blots of BRD4 degradation in K562 cells that were treated with DMSO or different concentrations of MMH1, MMH1-NR, MMH2 or MMH2-NR for 16 h. All western blot data are representative of two independent measurements. Source data

To determine the mechanism of DCAF16 recruitment, we sought to reconstitute the BRD4–DCAF16 interaction in a fully recombinant system. We developed a time-resolved fluorescence energy transfer (TR-FRET) assay (Extended Data Fig. 2b) and observed a tighter TMX1-induced interaction between DDB1–DCAF16 and BRD4_BD2_ compared to BRD4_BD1_, supporting the finding that the BD2 domain is the primary degron for TMX1-mediated degradation (Fig. 2a). We repeated a similar TR-FRET experiment with GNE11 and observed similar trends, but we found that the BRD4–DCAF16 interaction was much weaker compared to TMX1 (Extended Data Fig. 2c), consistent with the lower potency of GNE11 as a BRD4 degrader. These findings suggest that TMX1 functions as a molecular glue to recruit DCAF16 selectively to BRD4_BD2_, causing degradation of BRD4.

In TR-FRET experiments, the interaction of DCAF16 and BRD4_BD2_ unexpectedly decreased when the concentration of TMX1 exceeded 5 μM (Fig. 2a). We also observed decreases in reporter degradation at similar compound concentrations (Extended Data Fig. 1e). This pattern, referred to as a hook effect, is commonly seen with heterobifunctional degraders in which both compound–substrate and compound–ligase interactions become saturated at high ligand concentrations^23^. Hook effects are not observed with canonical molecular glues. Because TMX1 contains an electrophilic acrolein moiety, we hypothesized that TMX1 might form a covalent bond with DCAF16, thereby providing an alternative explanation for the hook effect.

To test whether TMX1 forms a covalent bond, we incubated recombinant DCAF16–DDB1 with TMX1 and performed intact mass spectrometry. We observed minimal (8%) modification of DCAF16 (Fig. 2b). Next, to see if the ternary complex might facilitate covalent bond formation, we incubated both recombinant BRD4_BD2_ and DCAF16–DDB1 with TMX1 and performed intact mass spectrometry. With both ubiquitin ligase and substrate present in the reaction, we observed 50% modification of DCAF16 (Fig. 2b). These data suggest that TMX1 has negligible reactivity with DCAF16 alone and that the presence of BRD4_BD2_ facilitates covalent modification, perhaps because it orients the acrolein warhead for attack by the cysteine in a mechanism that we refer to as ‘template-assisted covalent modification’. We observed similar modification with GNE11, albeit much weaker with only a 7% DCAF16 mass shift in the presence of BRD4_BD2_ even at extended timepoints (Extended Data Fig. 2d). The weaker reactivity of GNE11 is consistent with the propargylamine, although previously shown to be reactive^24^, being a weaker electrophile. As a control, we investigated the previously reported covalent DCAF16-dependent BRD4 heterobifunctional degrader KB02-JQ1 (ref. ^25^), which exhibited the expected covalent modification of DCAF16 regardless of whether BRD4_BD2_ was included in the reaction (Extended Data Fig. 2e). These studies demonstrate that the JQ1-derived molecular glue degraders act through a template-assisted covalent mechanism that is distinct from heterobifunctional degraders or traditional molecular glue degraders.

### Optimized warheads boost degrader potency and limit off-targets

**Extended Data Fig. 3:**
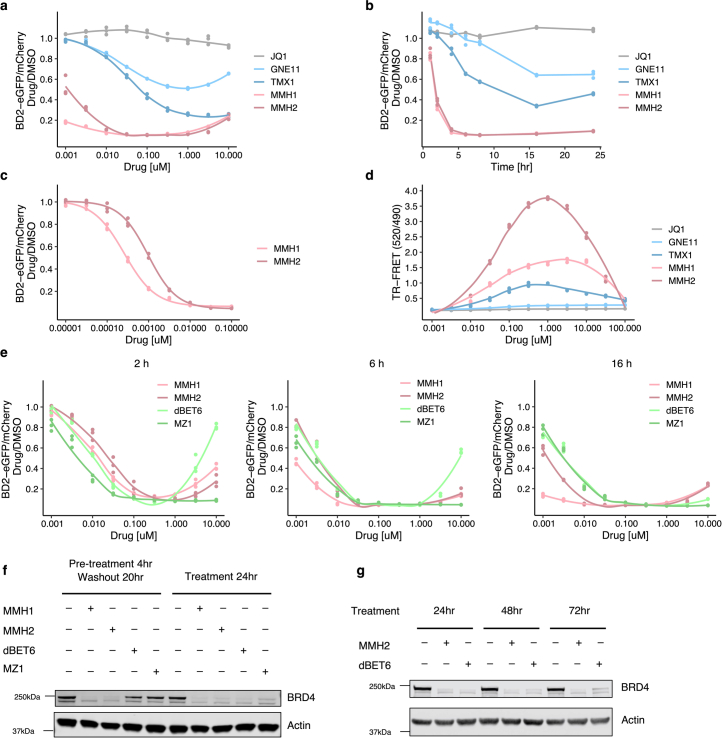
Optimized electrophilic warheads increases potency of degraders. **a**. Flow cytometry analysis of BRD4_BD2_-eGFP degradation in K562 cells that were treated with increasing concentrations of JQ1, GNE11, TMX1, MMH1 or MMH2 for 16 h (n = 3). **b**. Flow analysis of BRD4_BD2_-eGFP degradation in K562 cells that were treated with JQ1 at 1 μM, TMX1 at 1 μM, GNE11 at 1 μM, MMH1 at 0.1 μM or MMH2 at 0.1 μM for increasing time points (n = 3). **c**. Flow cytometry analysis of BRD4_BD2_-eGFP degradation in K562 cells that were treated with increasing concentrations of MMH1 or MMH2 for 16 h (n = 3). **d**. TR-FRET signal for DDB1-DCAF16-BODIPY to BRD4_BD2_-terbium with increasing concentrations of JQ1, GNE11, TMX1, MMH1 or MMH2 (n = 3). **e**. Flow cytometry analysis of BRD4_BD2_-eGFP degradation in K562 cells that were treated with increasing concentrations of MMH1, MMH2, dBET6 or MZ1 for 2 h, 6 h, or 16 h (n = 3). **f**. Western blot of BRD4 degradation in K562 cells pre-treated with MMH1, MMH2, dBET6 or MZ1 at 0.1 μM for 4 h, washed with PBS and resuspended in fresh media or the same drug-treated media for an additional 20 h. **g**. Western blots of BRD4 degradation in K562 cells that were treated with DMSO, MMH2 or dBET6 at 0.1 μM for 24, 48, or 72 h. All western blot data are representative of two independent measurements. Source data

The observation that a more reactive molecule, TMX1, demonstrated higher degradation potency than GNE11 suggests that optimization of the covalent warhead might improve the degradation activity of DCAF16-based BRD4 degraders. To test this hypothesis and to facilitate structural studies, we expanded the electrophilic chemotypes on the phenyl exit vector and characterized their BRD4_BD2_ degradation and DCAF16 recruitment activity using degradation and TR-FRET assays. We discovered an acrylamide analog, MMH1 (**3**), and a vinyl sulfonamide analog, MMH2 (**4**) (Fig. 2c), that both showed improved BRD4_BD2_ degradation activity (D_max_, _16 h_ ~ 95% and half-maximal degradation concentration at 16 h (DC_50, 16 h_) ~ 1 nM) (Extended Data Fig. 3a–c) and markedly stronger DCAF16 binding as compared to TMX1 (Extended Data Fig. 3d). When comparing MMH1-induced and MMH2-induced degradation of BRD4 with non-covalent BRD4 heterobifunctional degraders, dBET6 and MZ1 (refs. ^26,27^), we found that MMH1 and MMH2 exhibited similar BRD4 degradation (Fig. 2d and Extended Data Fig. 3e) with more sustained activity after washout or over an extended timecourse due to the covalent mechanism (Extended Data Fig. 3f,g).

**Extended Data Fig. 4:**
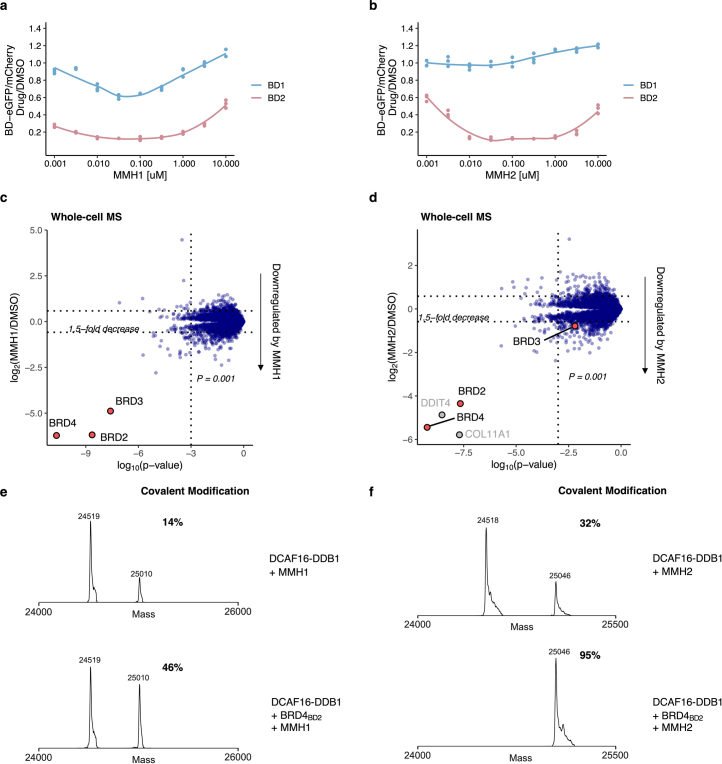
MMH1 and MMH2 conserve the mechanism of action as TMX1 and GNE11. **a**. Flow cytometry analysis of BRD4_BD1_-eGFP and BRD4_BD2_-eGFP degradation in K562 cells that were treated with increasing concentrations of MMH1 for 16 h (n = 3). **b**. Flow cytometry analysis of BRD4_BD1_-eGFP and BRD4_BD2_-eGFP degradation in K562 cells that were treated with increasing concentrations of MMH2 for 16 h (n = 3). **c**. Quantitative whole proteome analysis of K562 cells after treatment with MMH1 at 0.1 μM (n = 2) or DMSO (n = 4) for 5 h. Statistical analysis was performed using a two-sided moderated t-test as implemented in the limma package. **d**. Quantitative whole proteome analysis of K562 cells after treatment with MMH2 at 0.1 μM (n = 2) or DMSO (n = 4) for 5 h. Statistical analysis was performed using a two-sided moderated t-test as implemented in the limma package. **e**. Intact protein mass spectra of DDB1-DCAF16 co-incubated with MMH1 at 4 °C for 16 h, or DDB1-DCAF16 co-incubated with MMH1 and BRD4_BD2_ at 4 °C for 16 h. **f**. Intact protein mass spectra of DDB1-DCAF16 co-incubated with MMH2 at 4 °C for 16 h, or DDB1-DCAF16 co-incubated with MMH2 and BRD4_BD2_ at 4 °C for 16 h. Source data

To further confirm that covalent reactivity is critical for DCAF16 recruitment, we developed MMH1-NR (**5**) and MMH2-NR (**6**), containing a non-reactive (ethyl) group and a saturated vinyl moiety, respectively (Fig. 2c). Compared to their reactive analogs, both non-reactive molecules demonstrated negligible DCAF16 recruitment (Fig. 2e) or degradation activity (Fig. 2f), indicating that covalency is required for the activity of JQ1-derived DCAF16-based BRD4 degrader. To ensure that MMH1 and MMH2 conserve the mechanism of action of TMX1 and GNE11, we repeated the BD1/BD2 degradation assay (Extended Data Fig. 4a,b) and whole-cell proteomics experiments (Extended Data Fig. 4c,d) and observed similar results. Furthermore, we performed DCAF16 intact mass spectrometry experiments on MMH1 and MMH2 and found similar template-assisted covalent modifications (Extended Data Fig. 4e,f). However, the more reactive molecules, MMH1 and MMH2, also caused increased baseline, non-templated-assisted covalent labeling of DCAF16 (Extended Data Fig. 4e,f).

**Extended Data Fig. 5:**
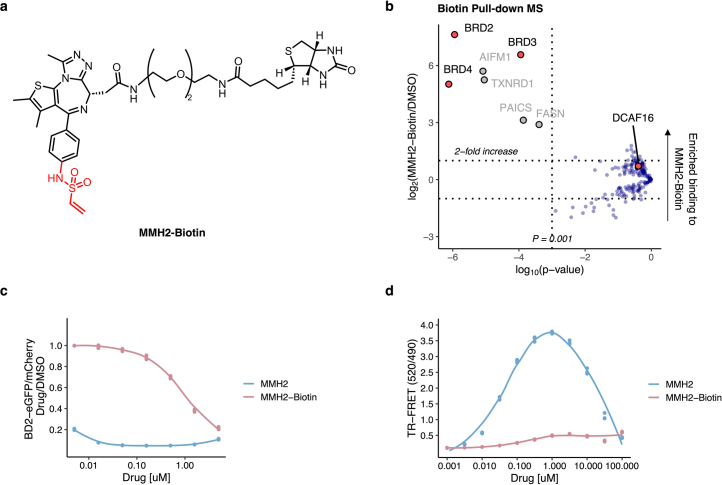
MMH2-Biotin characterization suggests limited off-target activity of covalent degraders. **a**. Chemical structure of MMH2-Biotin. **b**. Biotin pull-down followed by mass spectrometry in K562 cell lysates treated with either MMH2-Biotin at 1 μM (n = 3), or DMSO at 1 μM only (n = 3). Fold enrichment and p-values were calculated by comparing MMH2-Biotin treated samples to DMSO control samples. Statistical analysis was performed using a two-sided moderated t-test as implemented in the limma package. **c**. Flow cytometry analysis of BRD4_BD2_-eGFP degradation in K562 cells that were treated with increasing concentrations of MMH2 or MMH2-Biotin for 16 h (n = 3). **d**. TR-FRET signal for DDB1-DCAF16-BODIPY to BRD4_BD2_-terbium with increasing concentrations of MMH2 or MMH2-Biotin (n = 3). Source data

An issue with covalent warheads is off-target activity independent of reversible binding of the molecule. To profile the potential off-target effects of the more reactive degrader MMH2, we engineered MMH2-Biotin (**7**), a probe molecule containing MMH2 with a linker-biotin on the second exit vector of the JQ1 moiety (Extended Data Fig. 5a). Using MMH2-Biotin in pulldown mass spectrometry studies, we found that, apart from binding BET family proteins, only a very limited number of off-targets were observed, largely representing intrinsically reactive proteins (Extended Data Fig. 5b). These findings suggest that the covalent degraders that we developed are relatively specific with respect to off-target activities. The absence of DCAF16 enrichment is expected because the linker-biotin modification, inevitably given the tight interface, has substantially attenuated the probe’s ability to form ternary complex with DCAF16 and degrade BRD4 (Extended Data Fig. 5c,d).

### BRD4_BD2_ orients MMH2 for DCAF16 modification

**Fig. 3:**
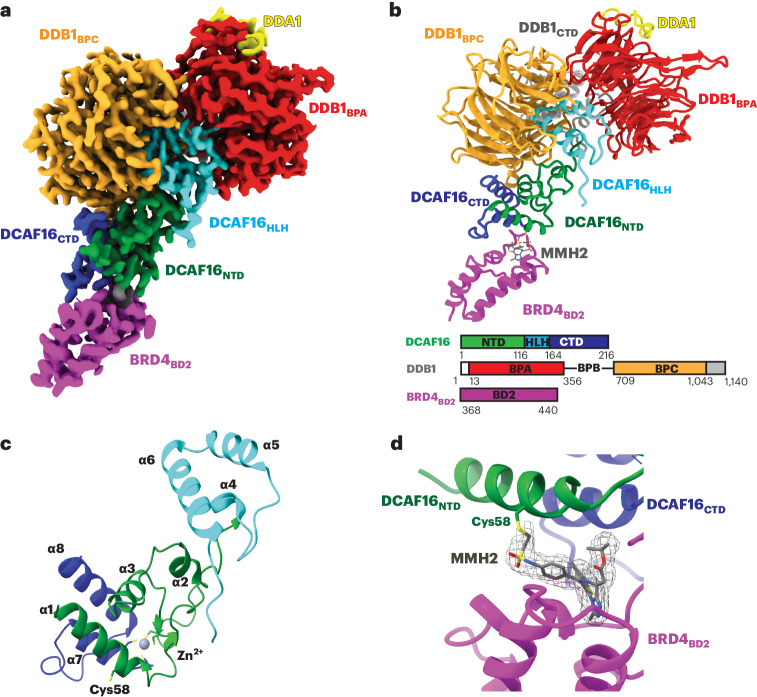
BRD4_BD2_ orients MMH2 for DCAF16 modification. **a**, 2.2-Å cryo-EM map of the DDB1ΔB–DDA1–DCAF16–BRD4_BD2_–MMH2 complex, colored to indicate DDB1_BPA_ (red), DDB1_BPC_ (orange), DDB1_CTD_ (gray), DDA1 (yellow), DCAF16_CTD_ (blue), DCAF16_NTD_ (green), DCAF16_HLH_ (cyan) and BRD4_BD2_ (magenta). The map shown was processed with DeepEMhancer^43^. **b**, Cartoon representation of the DDB1–DCAF16 ligase complex bound to BRD4_BD2_ and MMH2 with same coloring as the cryo-EM map. A sequence scheme for all complex partners is shown at the bottom. **c**, Cartoon representation of DCAF16 indicating secondary structure elements. **d**, Close-up of MMH2 covalently modifying DCAF16_Cys58_ with cryo-EM density around MMH2 shown as mesh.

**Extended Data Fig. 6:**
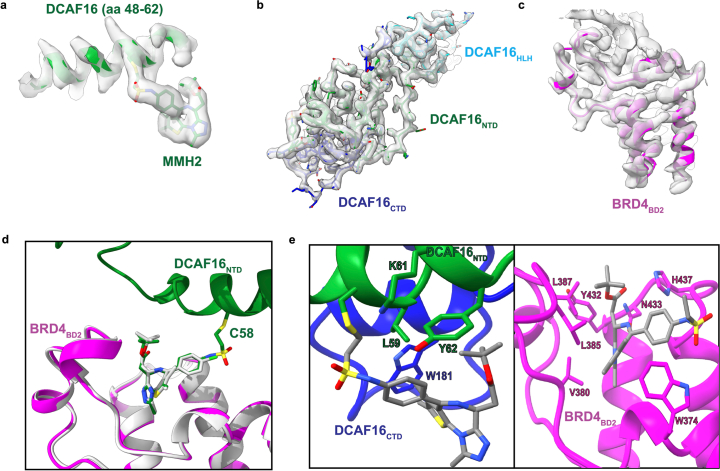
Map quality of the DCAF16-BRD4_BD2_-MMH2 interface. **a**. Cryo-EM density for DCAF16 containing Cys58 covalently bound to MMH2. Map contoured at 0.251. **b**. Cryo-EM density for DCAF16. **c**. Cryo-EM density for BRD4_BD2_. **d**. Overlay of MMH2 with JQ1 (PDB: 3ONI, in white). **e**. Key residues on DCAF16 (in green and blue) and BRD4_BD2_ (in magenta) close to MMH2.

To understand how BRD4_BD2_ facilitates covalent modification of DCAF16, we sought to structurally characterize the ternary complex by cryogenic electron microscopy (cryo-EM). Recombinant DDB1ΔB–DDA1–DCAF16 complex was mixed with recombinant BRD4_BD2_ and MMH2 and purified over size exclusion chromatography (SEC). A dataset was collected on a Titan Krios microscope after several rounds of grid optimization, leading to a condition containing 0.011% lauryl maltose neopentyl glycol (LMNG) detergent on UltrAuFoil grids that mitigated preferred orientations of the particles (see Methods for details). After several rounds of classification, a final reconstruction was refined to 2.2 Å and used for model building (Fig. 3a,b, Supplementary Fig. 3a–f and Supplementary Table 1). DDB1ΔB and BRD4_BD2_ were readily placed into the density using high-resolution structures Protein Data Bank (PDB): 6Q0R and PDB: 6VIX as models. The density filling the gap between DDB1 and BRD4_BD2_ was identified as DCAF16, and a model was manually built (Fig. 3b, Extended Data Fig. 6a–c, Supplementary Fig. 3a–f and Supplementary Table 1).

DCAF16 folds into a structure without any homologies across the PDB or AlphaFold2 databases^28,29^. DCAF16 is anchored to DDB1 with a centrally located helix-loop-helix (HLH, amino acids 113–155) motif distinct from canonical DCAFs that occupies a similar spatial location to the DDB1-binding motif of CRBN. The amino-terminal and carboxy-terminal regions of DCAF16 fold into a four-helix bundle stabilized by a zinc atom forming the primary interface with BRD4_BD2_ (Fig. 3c). The first helix (α1) is followed by an extended loop toward DDB1 with a short helix (α2) packing against the HLH motif. The next helix (α3) packs on top of α1 and, together with α7 and α8, forms the core of the structure. After another extended loop and short helix (α4) back toward DDB1, the HLH motif is formed by α5, α6 and several smaller loops filling the DDB1 cavity. Returning from the HLH motif, another extended loop leads back to the BRD4_BD2_-interacting region forming α7, followed by a loop embracing BRD4_BD2_, and α8, completing the core structure.

DCAF16 embraces BRD4_BD2_ with major contacts contributed by α1, α7 and the loop between α7 and α8 (Fig. 3b–d and Extended Data Fig. 6d) for a total interface area of 560 Å^2^, as assessed using the PISA server^30^. At the interface between DCAF16 and BRD4_BD2_, we observed a density representing MMH2, overlapping with the JQ1 binding site of BRD4_BD2_ (Fig. 3d and Extended Data Fig. 6d). In line with a covalent mechanism, continuous density was observed between MMH2 and Cys58 on DCAF16 (Fig. 3d), with the right geometry and distances for a covalent bond. Additionally, key contacts between MMH2 and DCAF16 (Leu59, Lys61, Tyr62 and Trp181) and BRD4_BD2_ (including Trp374, Val380, Leu385, Leu387, Tyr432, Asn433 and His437), respectively, contribute to the DCAF16–BRD4_BD2_ interface (Extended Data Fig. 6e). Together, the structure and biochemical characterization support a model in which MMH2 binds BRD4_BD2_, leading to recruitment of DCAF16 and orientation of MMH2 for modification of DCAF16_Cys58_. Our data further suggest that this covalent modification of DCAF16 is necessary to stabilize the ternary complex sufficiently for ubiquitylation and consequent degradation to occur.

### DCAF16_Cys58_ is targeted by molecular glue degraders

**Fig. 4:**
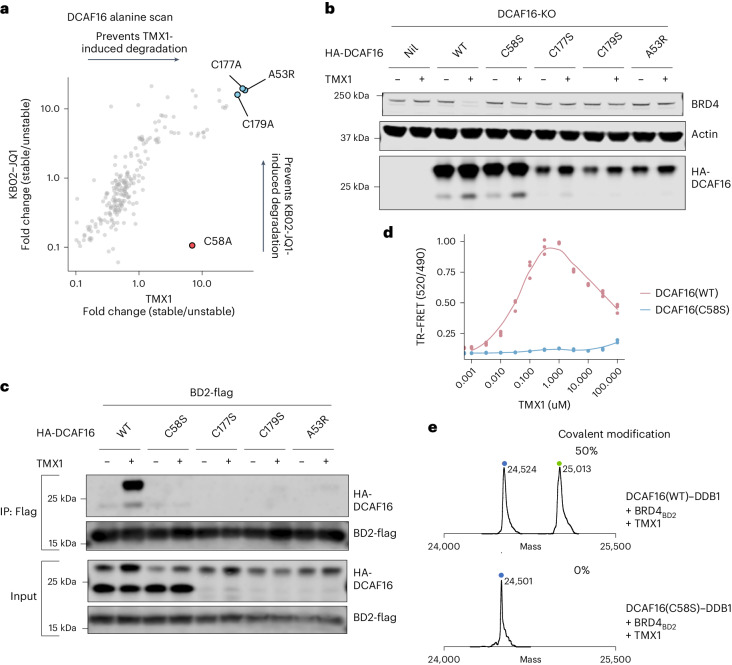
DCAF16_Cys58_ is targeted by molecular glue degraders. **a**, Correlation of fold change for two DCAF16 alanine scans in DCAF16-knockout K562 cells. The *x* axis is a degradation screen for BRD4_BD2_-eGFP upon treatment with TMX1 at 1 μM for 16 h (*n* = 3), and the *y* axis is another degradation screen for BRD4_BD2_-eGFP upon treatment with KB02-JQ1 at 10 μM for 16 h (*n* = 3). **b**, Western blots of BRD4 degradation in DCAF16-knockout K562 cells that were transduced with indicated HA-DCAF16 mutants and treated with DMSO or TMX1 at 1 μM for 16 h. **c**, Flag IP followed by western blots in the presence of DMSO or TMX1 at 1 μM from 293T cells transfected with indicated HA-DCAF16 mutants and BRD4_BD2_-Flag constructs. **d**, TR-FRET signal for DDB1–DCAF16(WT) or DDB1–DCAF16(C58S)–BODIPY to BRD4_BD2_-terbium with increasing concentrations of TMX1 (*n* = 3). **e**, Intact protein mass spectra of DDB1–DCAF16(WT) or DDB1–DCAF16(C58S) co-incubated with TMX1 and BRD4_BD2_ at 4 °C for 16 h. All western blot data are representative of two independent measurements. KO, knockout; WT, wild-type. Source data

**Extended Data Fig. 7:**
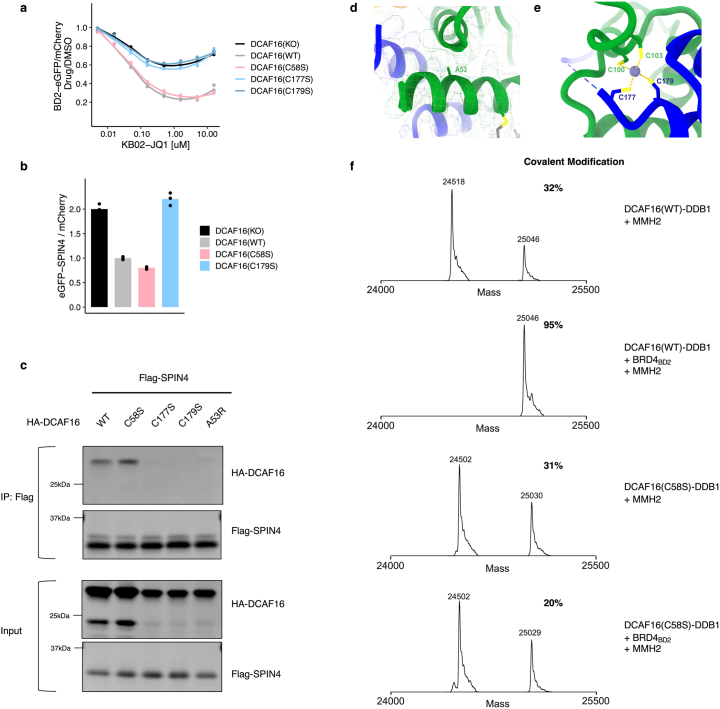
Validation of DCAF16 mutants. **a**. Flow cytometry analysis of BRD4_BD2_-eGFP degradation in DCAF16 knockout K562 cells transduced with indicated HA-DCAF16 mutants and treated with increasing concentrations of KB02-JQ1 for 16 h (n = 3). **b**. Flow cytometry analysis of eGFP-SPIN4 stability in DCAF16 knockout K562 cells transduced with indicated HA-DCAF16 mutants (n = 3). **c**. Western blots of Flag immunoprecipitation (IP) from 293 T cells transfected with indicated HA-DCAF16 mutants and Flag-SPIN4 constructs. **d**. Close up of DCAF16 Ala53 orienting towards the hydrophobic core. **e**. Close up of DCAF16 Cys177 and Cys179 coordinating a structural zinc ion. **f**. Intact protein mass spectra of DDB1-DCAF16(WT) co-incubated with MMH2, DDB1-DCAF16(WT) co-incubated with MMH2 and BRD4_BD2_, DDB1-DCAF16(C58S) co-incubated with MMH2, or DDB1-DCAF16(C58S) co-incubated with MMH2 and BRD4_BD2_ at 4 °C for 16 h. All western blot data are representative of two independent measurements. Source data

To further corroborate the structural findings in an unbiased fashion, we performed a systematic alanine scan on all residues of DCAF16 and evaluated drug-induced BRD4_BD2_ reporter degradation in a pooled screening format (Supplementary Fig. 4a). A53R, C177A and C179A mutants scored as the top hits in those screens with all the molecular glue degraders (Fig. 4a and Supplementary Fig. 4b–e). We validated that these mutations prevent both drug-induced BRD4 degradation (Fig. 4b and Supplementary Fig. 5a–c) and DCAF16–BRD4_BD2_ binding (Fig. 4c and Supplementary Fig. 6a–c). These same three amino acids scored when we performed the screen with KB02-JQ1, a DCAF16-dependent BRD4 PROTAC^25^ (Extended Data Fig. 7a and Supplementary Fig. 4f). In addition, the same three top amino acids also scored when we screened for degradation of SPIN4, a previously reported endogenous substrate of DCAF16 (ref. ^31^) (Extended Data Fig. 7b,c and Supplementary Fig. 4g). These results indicate that Ala53, Cys177 and Cys179 are critical for the general E3 ubiquitin ligase function of DCAF16 but are not specific to template-assisted covalent interactions with the BRD4 molecular glue degraders. These residues are critical for DCAF16 structural integrity as Ala53 oriented toward the hydrophobic core, and Cys177 and Cys179 coordinate a structural zinc ion (Extended Data Fig. 7d,e).

Only one cysteine residue, Cys58, was required exclusively for the activity of the molecular glue degraders but not for KB02-JQ1 activity or SPIN4 degradation (Fig. 4a and Supplementary Fig. 4b–g). We confirmed the Cys58-selective effect on binding and degradation using co-immunoprecipitation, TR-FRET, western blots and degradation assays (Fig. 4b–d, Extended Data Fig. 7a–c and Supplementary Figs. 5a–c and 6a–f). We also expressed and purified recombinant DCAF16 protein with Cys58 mutated to serine. By intact mass spectrometry, the DCAF16 C58S mutant completely eliminated DCAF16–TMX1 adduct formation (Fig. 4e). We also performed intact mass spectrometry analysis on wild-type and C58S mutant DCAF16 co-incubated with BRD4_BD2_ and MMH2, showing that DCAF16 C58S mutant greatly reduces adduct formation from 95% to 20%, close to the baseline labeling efficiency of MMH2 without the presence of BRD4_BD2_ template (Extended Data Fig. 7f). Collectively, these results validate the structural insight that DCAF16_Cys58_ is the amino acid targeted for template-assisted covalent modification by the BRD4 molecular glue degraders.

**Extended Data Fig. 8:**
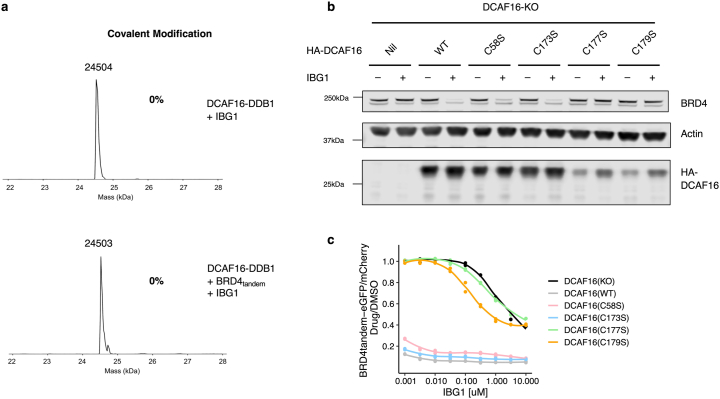
Characterization of IBG1. **a**. Intact protein mass spectra of DDB1-DCAF16 co-incubated with IBG1 at 4 °C for 16 hours or DDB1-DCAF16 co-incubated with IBG1 and BRD4_tandem_ at 4 °C for 16 hours. **b**. Western blots of BRD4 degradation in DCAF16 knockout K562 cells that were transduced with indicated HA-DCAF16 mutants, and treated with DMSO or IBG1 at 1 μM for 16 h. **c**. Flow cytometry analysis of BRD4_tandem_-eGFP degradation in DCAF16 knockout K562 cells transduced with indicated HA-DCAF16 mutants and treated with increasing concentrations of IBG1 for 16 h (n = 3). All western blot data are representative of two independent measurements. Source data

Notably, a separate study introduced IBG1 (ref. ^32^), a bivalent degrader that reinforces an intrinsic interaction between DCAF16 and BRD4_tandem_ (a BRD4 construct containing both BD1 and BD2 bromodomains connected by the native linker). To examine whether IBG1 engages DCAF16 through a mechanism similar to or distinct from our covalent molecular glues, we performed intact mass spectrometry experiments on recombinant DCAF16 co-incubated with IBG1, with or without the addition of recombinant BRD4_tandem_, and found no evidence of covalent modifications on DCAF16 (Extended Data Fig. 8a). Additionally, we conducted western blots and BRD4_tandem_ reporter assays with DCAF16-knockout K562 cell lines ectopically expressing different DCAF16 mutants. We observed that only the structural mutants C177S and C179S, but not the reactive C58S mutants, were able to prevent IBG1-induced BRD4 degradation (Extended Data Fig. 8b,c). These findings clearly demonstrate that IBG1 operates through a distinct, non-covalent mechanism.

### Residues crucial for BRD4_BD2_ conformation confer selectivity

**Fig. 5:**
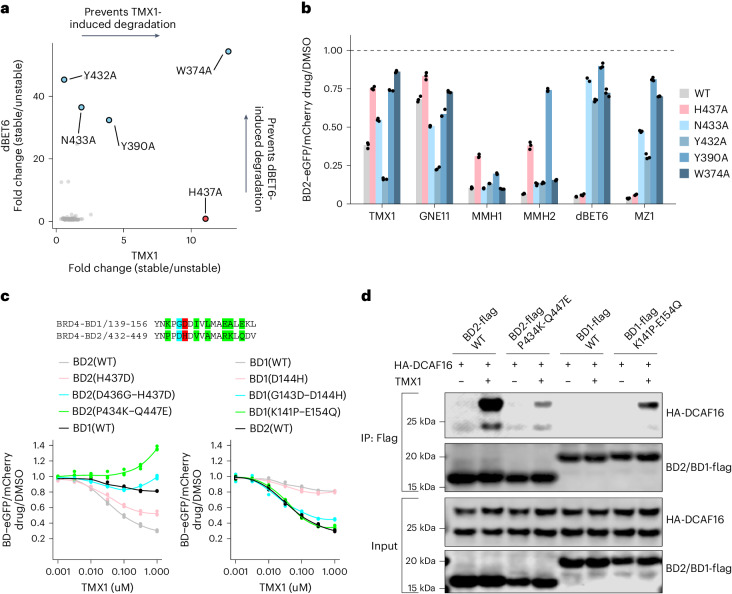
Residues crucial for BRD4_BD2_ conformation confer selectivity. **a**, Correlation of fold change for two BRD4_BD2_ alanine mutagenesis screens. The *x* axis is a degradation screen for BRD4_BD2_-eGFP in K562 cells upon treatment with TMX1 at 1 μM for 16 h (*n* = 2), and the *y* axis is another degradation screen for BRD4_BD2_-eGFP in K562 cells upon treatment with dBET6 at 1 μM for 16 h (*n* = 2). **b**, Flow cytometry analysis of K562 cells expressing wild-type or indicated mutant BRD4_BD2_-eGFP construct and treated with DMSO, GNE11 at 1 μM, TMX1 at 1 μM, MMH1 at 0.1 μM, MMH2 at 0.1 μM, MZ1 at 1 μM or dBET6 at 1 μM for 16 h. Biologically independent replicates are shown (*n* = 3). **c**, Flow cytometry analysis of K562 cells expressing the indicated BRD4_BD2_-eGFP, BRD4_BD1_-eGFP mutant construct and treated with increasing concentrations of TMX1 for 16 h (*n* = 3). **d**, Flag IP followed by western blots in the presence of DMSO or TMX1 at 1 μM from 293T cells transfected with HA-DCAF16 and indicated BRD4_BD2_-Flag, BRD4_BD1_-Flag mutant constructs. All western blot data are representative of two independent measurements. WT, wild-type. Source data

**Extended Data Fig. 9:**
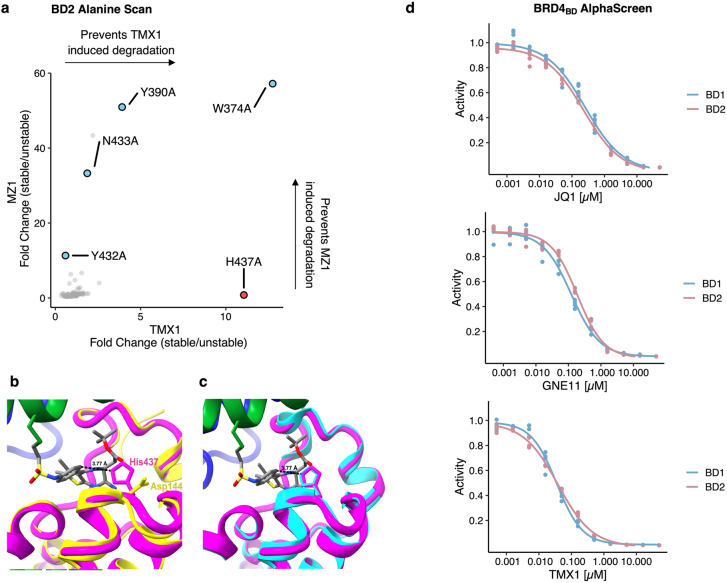
Mechanism of bromodomain selectivity. **a**. Correlation of fold change for two BRD4_BD2_ alanine mutagenesis screens. The *x* axis is a degradation screen for BRD4_BD2_-eGFP in K562 cells upon treatment with TMX1 at 1 μM for 16 h (n = 2), and the *y* axis is another degradation screen for BRD4_BD2_-eGFP in K562 cells upon treatment with MZ1 at 1 μM for 16 h (n = 2). **b**. Overlay of BRD4_BD1_ (PDB: 3MXF, in yellow) with BRD4_BD2_ (in magenta) showing a close-up of residues His437. When substituted for Asp144 in BRD4_BD1,_ there is repulsion between Asp144 and the JQ1 carbonyl. **c**. Overlay of BRD2_BD2_ (PDB: 3ONI, in cyan) with BRD4_BD2_ (in magenta) showing a close-up of residues His437 and the corresponding His433 in BRD2_BD2._ **d**. AlphaScreen competitive assay of JQ1, GNE11, and TMX1 to quantify the drug’s inhibition of binding between biotinylated-JQ1 and His-tagged BRD4_BD1_ or BRD4_BD2_ (n = 4). Source data

Because we observed a selectivity for BRD4_BD2_, despite close homology of the BD1 and BD2 domains around the drug binding site and similar affinities for JQ1, we set out to dissect the residues on BRD4_BD2_ critical for degradation with a systematic alanine scan (Supplementary Fig. 7a). For BRD4_BD2_, His437 was the most critical residue for activity of molecular glue degraders, but not heterobifunctional degraders, indicating that it is functionally important for drug-induced DCAF16–BRD4_BD2_ recruitment (Fig. 5a, Extended Data Fig. 9a and Supplementary Fig. 7b–g). Known JQ1 contacting residues, including Asn433, Tyr432, Tyr390 and Trp374 (refs. ^20,33^), also scored as amino acids required for dBET6-induced and MZ1-induced degradation (Fig. 5a, Extended Data Fig. 9a and Supplementary Fig. 7f,g). These findings were validated using individual alanine mutants with consistent results (Fig. 5b).

When comparing our structure with JQ1-bound structures of BD1 (PDB: 3MXF) or BD2 (PDB: 3ONI), the only notable differences are in the loop containing His437, closing onto the JQ1 pocket (Extended Data Fig. 9b,c). His437 contacts a carbonyl of JQ1-based degraders and potentially contributes weak interactions with Tyr62 of DCAF16 (Extended Data Fig. 6e). We, therefore, tested whether His437 is critical for the BD2 selectivity of covalent BRD4 molecular glue degraders. We constructed BRD4_BD1_ and BRD4_BD2_ domains, swapping the respective amino acid residues near His437. Using reporter degradation assays, we found that the BRD4_BD2_(D436G-H437D) and BRD4_BD2_(P434K-Q447E) mutants were resistant to TMX1-induced degradation, whereas the corresponding BRD4_BD1_(G143D-D144H) and BRD4_BD1_(K141P-E154Q) mutants gained susceptibility to TMX1-induced degradation compared to wild-type BRD4_BD1_ (Fig. 5c). The same amino acid swap in BRD4_BD2_ decreased TMX1-induced binding to DCAF16 and increased drug-induced BRD4_BD1_–DCAF16 binding (Fig. 5d). Given that TMX1 has similar binding affinity to both BD1 and BD2 domains of BRD4 (Extended Data Fig. 9d), the BD2 selectivity of drug-induced degradation is likely driven by differences in protein–protein interactions between BRD4_BD2_ and DCAF16 and orientation of the reactive warhead with respect to DCAF16_Cys58_. Although His437 directly contributes to binding of DCAF16, Asp436, Pro434 and Gln447 are not at the DCAF16 interface and contribute to the overall bromodomain conformation.

### Template-assisted covalent modification of GAK

**Fig. 6:**
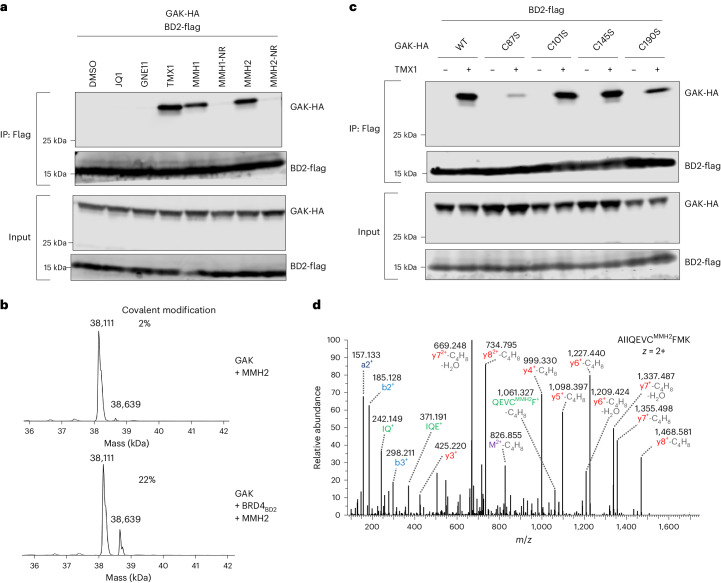
Template-assisted covalent modification of GAK. **a**, Flag IP followed by western blots in the presence of DMSO, JQ1, GNE11, TMX1, MMH1, MMH1-NR, MMH2 or MMH2-NR at 1 μM from 293T cells transfected with GAK_14–351_-HA and BRD4_BD2_-Flag constructs. **b**, Deconvoluted spectra for intact protein mass spectrometry experiments of GAK_14–351_ co-incubated with MMH2 at 4 °C for 16 h or GAK_14–351_ co-incubated with MMH2 and BRD4_BD2_ at 4 °C for 16 h. **c**, Flag IP followed by western blots in the presence of DMSO or TMX1 at 1 μM from 293T cells transfected with BRD4_BD2_-Flag and indicated cysteine mutant of GAK_14–351_-HA constructs. **d**, MS/MS spectrum of GAK peptide containing MMH2-modified cysteine (amino acids 81–90). All western blot data are representative of two independent measurements. WT, wild-type. Source data

**Extended Data Fig. 10:**
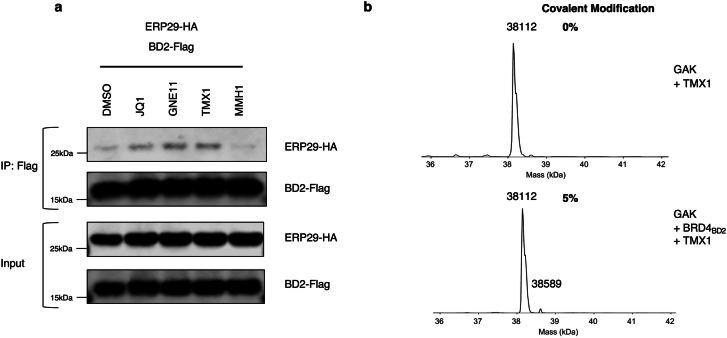
Template-assisted covalent modification of GAK. **a**. Flag immunoprecipitation (IP) followed by Western blots in the presence of DMSO, JQ1, GNE11, TMX1 or MMH1 at 1 μM from 293 T cells transfected with ERP29-HA and BRD4_BD2_-Flag constructs. **b**. Deconvoluted spectra for intact protein mass spectrometry experiments of GAK_14-351_ co-incubated with TMX1 at 4 °C for 16 hours or GAK_14-351_ co-incubated with TMX1 and BRD4_BD2_ at 4 °C for 16 hours. All western blot data are representative of two independent measurements. Source data

Finally, we sought to determine whether template-assisted covalent modification can be extended beyond DCAF16. We took advantage of the off-target activity of some JQ1-derived covalent compounds to identify candidate protein–protein interactions that are potentially mediated by this *trans*-labeling mechanism. Using IP-MS experiments, we found two additional proteins, GAK and ERP29, that bound BRD4_BD2_ in the presence of TMX1 (Fig. 1f). Next, we performed co-immunoprecipitation experiments with JQ1 covalent and non-covalent analogs. We found that GAK–BRD4_BD2_ interaction is induced by strong covalent analogs (TMX1, MMH1 and MMH2) but not weak (GNE11) or non-covalent (JQ1, MMH1-NR and MMH2-NR) analogs (Fig. 6a). This covalent dependency was absent in the case of ERP29 (Extended Data Fig. 10a). These findings suggest that GAK, but not ERP29, is covalently modified by JQ1-derived molecule glue compounds.

To examine whether the drug-induced GAK–BRD4_BD2_ interaction is mediated by template-assisted covalent modification, we conducted intact mass spectrometry experiments to examine the modification of GAK by MMH2 in the absence or presence of BRD4_BD2_. Mirroring our findings with DCAF16, we observed only approximately 2% of MMH2 adduct on GAK in the absence of BRD4_BD2_. However, in the presence of BRD4_BD2_, we detected approximately 22% of MMH2 adduct on GAK (Fig. 6b). Likewise, without BRD4_BD2_, no detectable TMX1 adduct was observed on GAK, but, with its presence, we detected approximately 5% of TMX1 adduct on GAK (Extended Data Fig. 10b). These results establish the role of BRD4_BD2_ as a structural template in facilitating labeling of GAK with MMH2 and TMX1. To pinpoint the specific cysteine residue on GAK targeted by template-assisted covalent modification, we conducted co-immunoprecipitation experiments using GAK cysteine mutants. We identified Cys87 as the crucial cysteine residue required for drug-induced GAK–BRD4_BD2_ binding (Fig. 6c), which was further confirmed by mass spectrometry (Fig. 6d).

Collectively, our findings underscore that the MMH2-induced and TMX1-induced GAK–BRD4_BD2_ interaction represents another example of template-assisted covalent modification, distinct from the DCAF16–BRD4_BD2_ pair that we previously elucidated. These data suggest that the *trans*-labeling mechanism that we discovered is generalizable and can be leveraged to identify neosubstrate–ligase pairs by testing compound libraries targeting other non-BRD4 proteins.

## Discussion

Our studies reveal a class of molecular glue degraders that act through template-assisted covalent modification and establish a mechanism for how *trans*-labeling can stabilize a molecular glue-induced neo-protein–protein interface, informing future discovery and design of molecular glues. Combining cellular, biochemical and structural studies, we found that the BD2 domain of BRD4, in complex with DCAF16, serves as a structural scaffold to orient the reactive moiety of a small molecule for covalent modification of DCAF16 and degradation of BRD4. This templated reactivity has the potential to increase the affinity of other complementary protein surfaces, such as observed for the drug-induced GAK–BRD4_BD2_ interaction, resulting in novel molecular glues to drive protein degradation or other biological processes. Similarly, kinase-catalyzed transfer of the electrophilic terminal phosphate group of ATP to substrate proteins can be viewed as a template (kinase)-assisted covalent modification. More broadly, it is likely that many sites observed in chemo-proteomics covalent fragment screens^34,35^, and especially those observed in unstructured regions, may be the result of a similar template-assisted mechanism in which the primary binding energy is derived from a binding partner.

The covalent property of the BRD4 degraders leads to a hook effect and more durable degradation, distinguishing covalent from non-covalent molecular glue degraders. Our studies also reveal that modulating the reactivity of the electrophilic warhead can tune the activity and specificity of covalent molecular glue degraders. In the case of the JQ1-derived molecules, non-reactive molecules did not induce protein degradation, and highly reactive molecules may lack specificity. Both orientation of the covalent warhead and degree of reactivity optimizes activity and specificity. Moreover, we discovered that the inherent structural compatibility between the two proteins flanking the molecular glue compound profoundly enhances the compound’s covalent reactivity toward a specific amino acid. The comparison between the templated covalent degrader, TMX1, and the non-templated covalent degrader, KB02-JQ1, revealed that TMX1 achieves a more selective covalent modification of DCAF16 while effectively degrading BRD4. Our MMH2-Biotin pulldown experiment also supports that the covalent degraders that we developed exhibit relatively limited off-target activities. These findings underscore the potential of template-assisted covalent modification as a promising avenue for engineering covalent molecular glue degraders with enhanced efficiency and specificity compared to their non-templated counterparts.

DCAF16 is well suited for template-assisted covalent modification because its cysteine-rich substrate binding surface is readily targeted for covalent modification, as demonstrated by our work and the prior identification of heterobifunctional degraders targeting DCAF16 (ref. ^25^). We determined the cryo-EM structure of the DDB1–DCAF16 ligase complex bound to BRD4_BD2_ and MMH2, providing definite proof of this molecular glue interaction. Unlike most other DCAF proteins, DCAF16 does not contain a canonical WD40 propeller^36^ and, instead, is a relatively unstable protein predicted to be largely unstructured. It is noteworthy that our structural studies suggest a high degree of conformational flexibility, similar to findings from studies of CRBN^37–39^, and we speculate that such structural plasticity in a ligase can facilitate glue activity.

Consistent with recent work regarding the mechanism of action for GNE11 (ref. ^40^), we showed that the activity of GNE11 is dependent on DCAF16. Furthermore, we present experimental data demonstrating that GNE11 works as a covalent molecular glue degrader for BRD4. In a separate manuscript, the authors reported IBG1, a PROTAC-like degrader that stabilizes an intrinsic interaction between BRD4_tandem_ and DCAF16 (ref. ^32^). We investigated IBG1 and confirmed that it is non-covalent and causes BRD4 degradation through a distinct mechanism.

A central challenge for the development of molecular glue degraders is the need for approaches for rational drug design and discovery^41^. In our current study, we demonstrate that the addition of electrophiles to the solvent-exposed side of JQ1 results in DCAF16-dependent covalent molecular glue degraders. The addition of electrophilic warheads to protein binders could become an effective strategy to stabilize a ternary complex and enable protein degradation when similar non-covalent molecules do not^42^. We anticipate that the judicious integration of solvent-exposed electrophiles into protein binders, coupled with template-assisted covalent modification strategies, will empower the design of novel and superior degraders than is currently possible.

## Methods

### Mammalian cell culture

The human HEK293T and HEK293T–Cas9 cell lines were provided by the Genetic Perturbation Platform, Broad Institute. The K562–Cas9 cell line was provided by Zuzana Tothova (Dana-Farber Cancer Institute). All cell lines were authenticated with STR profiling. HEK293T and HEK293T–Cas9 cells were cultured in DMEM (Gibco), and K562–Cas9 cell lines were cultured in RPMI (Gibco), with 10% FBS (Invitrogen), 5% glutamine and penicillin–streptomycin (Invitrogen) at 37 °C and 5% CO_2_.

### Antibodies

The following antibodies were used: anti-BRD4 (Bethyl Laboratories, A301-985A100, polyclonal, 1:1,000 dilution), anti-β-actin (Cell Signaling Technology, 3700, clone 8H10D10, 1:10,000 dilution), anti-Flag (Sigma-Aldrich, F1804, clone M2, 1:1,000 dilution), anti-HA (Cell Signaling Technology, 3724, clone C29F4, 1:1,000 dilution), IRDye 800CW goat anti-mouse IgG secondary antibody (LI-COR Biosciences, 926-32210, 1:10,000 dilution) and IRDye 680LT goat anti-rabbit IgG secondary antibody (LI-COR Biosciences, 926-68021, 1:10,000 dilution).

### Compounds

JQ1 (HY-13030), dBET6 (HY-112588), MZ1 (HY-107425), KB02-JQ1 (HY-129917) and MLN4924 (HY-70062) were obtained from MedChemExpress. MLN7243 (CT-M7243) was obtained from ChemieTek. MG132 (S2619) was obtained from Selleck Chemicals. IBG1 was kindly provided by Alessio Ciulli’s group. GNE11, TMX1, MMH1, MMH2, MMH1-NR, MMH2-NR and MMH2-Biotin were synthesized in-house (for synthetic chemistry methods, see Supplementary Note: Synthesis of compounds, characterization and spectra).

### Plasmids

The following plasmids were used in this study: Cilantro (PGK.BsmBICloneSite.FlexibleLinker.eGFP.IRES.mCherry.cppt.EF1α.PuroR, Addgene, 74450) and Lavender (PGK.eGFP.FlexibleLinker.BsmBICloneSite.IRES.mCherry.cppt.EF1α.PuroR) for degradation characterization, reporter CRISPR screen, BRD4_BD2_ alanine scan and DCAF16 alanine scan; sgBFP (U6.sgRNA.cppt.SFFV.tBFP) and sgRFP (U6.sgRNA.cppt.EF1α.RFP657) for validation of DCAF16-knockout phenotypes; Mint-Flag/HA (SFFV.BsmBICloneSite.Flag/HA.cppt.EF1α.PuroR) and Ivy-Flag/HA (SFFV.Flag/HA.BsmBICloneSite.cppt.EF1α.PuroR) for co-immunoprecipitation and DCAF16 mutant transduction; pAC8-derived plasmids, E.Coli pET100/D-TOPO and pNIC-Bio2 for protein purification.

### Immunoblots

Cells were washed with PBS and lysed in RIPA lysis buffer (Thermo Fisher Scientific) with Halt Protease Inhibitor Cocktail (Thermo Fisher Scientific) and Benzonase (Sigma-Aldrich) for 20 min on ice. The insoluble fraction was removed by centrifugation; the protein concentration was quantified using a BCA protein assay kit (Thermo Fisher Scientific); and an equal amount of lysate was run on SDS-PAGE 4–12% Bis-Tris protein gels (Thermo Fisher Scientific) and then transferred to nitrocellulose membrane with an XCell II Blot Module Wet Tank Transfer System (Thermo Fisher Scientific). Membranes were blocked in Intercept (PBS) Blocking Buffer (LI-COR Biosciences) and incubated with primary antibodies overnight at 4 °C. The membranes were then washed in Tris-buffered saline with Tween 20 (TBS-T), incubated for 1 h with secondary IRDye-conjugated antibodies (LI-COR Biosciences) and washed three times in TBS-T for 5 min before near-infrared western blot detection on an Odyssey Imaging System with Image Studio software (LI-COR Biosciences).

### Co-immunoprecipitation

A total of 3 × 10^6^ HEK293T cells were plated into 10-cm dishes, cultured for 1 d and transfected with 9 μg of HA-tagged and 9 μg of Flag-tagged constructs using TransIT-LT1 transfection reagents (Mirus). The transfected cells were cultured for another 2 d, treated with 1 μM MLN4924 and co-treated with either degrader or DMSO for 4 h before collection. The cells were collected and lysed in Pierce IP Lysis Buffer (Thermo Fisher Scientific) with Halt Protease Inhibitor Cocktail (Thermo Fisher Scientific) for 20 min on ice and centrifuged for 15 min to remove the insoluble fraction. Degrader was infused to all buffers used for the degrader-treated arm. For IP, 25 μl of pre-cleaned anti-Flag magnetic beads (Sigma-Aldrich) was added to the lysates. The beads–lysate mix was incubated at 4 °C for 2 h on a rotator. Beads were magnetically removed and washed five times with Pierce IP Lysis Buffer before boiling in 1× NuPAGE LDS Sample Buffer (Thermo Fisher Scientific). Immunoblotting was done as described above.

### Reporter cell line generation

Reporter constructs were generated by BsmBI (New England Biolabs) digestion of Cilantro or Lavender reporter vector and the insert containing protein of interest coding sequence, followed by ligation with T4 DNA Ligase (New England Biolabs). Constructs were transformed into Stbl3 *E. coli* and purified using a MiniPrep Kit (Qiagen), and sequences were confirmed by Sanger sequencing (Quintara Biosciences). Lentiviruses for reporters were packaged into lentivirus as follows. First, 0.5 × 10^6^ HEK293T cells were seeded in 2 ml of DMEM media. The next day, a packaging mix including 1.5 μg of psPAX2, 0.15 μg of pVSV-G and 1.5 μg of transgene plasmid was prepared in 37.5 µl of OptiMEM (Thermo Fisher Scientific). This mix was combined with 9 μl of TransIT-LT1 (Mirus) and 15 µl of OptiMEM, incubated for 30 min at room temperature and then applied dropwise to cells. Cells were allowed to incubate for another 48 h. Lentivirus was collected by 0.4 μM filters and then transduced to 2 × 10^6^ of K562–Cas9 or 293T–Cas9 cells at 50% volume ratio by spin infection. One day after infection, reporter cells were selected with puromycin at a concentration of 2 μg ml^−1^.

### Pooled and single-clone knockout cell line generation

sgRNAs targeting DCAF16 (sgDCAF16) or control (sgNTC) were cloned into the sgBFP or sgRFP vector using BsmBI cloning. In brief, vectors were linearized with BsmBI (New England Biolabs) and gel purified with a QIAquick Gel Extraction Kit (Qiagen). Annealed oligos containing sgRNA sequences were phosphorylated with T4 polynucleotide kinase (New England Biolabs) and ligated into linearized vector backbone. sgRNA constructs were transformed, purified and verified, and lentivirus was generated as described above. Lentivirus containing sgRNA was transduced to 2 × 10^6^ K562–Cas9 cells at 10% volume ratio by spin infection. Fluorescence-activated cell sorting (FACS) was performed to enrich BFP^+^ or RFP^+^ cells 1 week after infection. For the generation of single-clone DCAF16-knockout cells, pooled K562–Cas9 cells stably expressing sgRNA targeting DCAF16 were seeded in 384-well plates at the density of 0.25 cells per well. Clonal sgDCAF16-expressing K562–Cas9 cells were isolated after 1 month of expansion, and the genomic sequences were validated via deep sequencing of PCR amplicons targeting sgDCAF16 cutting sites (Massachusetts General Hospital Center for Computational and Integrative Biology DNA Core Service).

### Reporter degradation assays

K562 cells stably expressing degradation reporter were dosed with DMSO or degraders at various times and concentrations using D300e Digital Dispenser (HP). The fluorescent signal was quantified by flow cytometry (LSRFortessa flow cytometer with BD FACSDiva 8.0 software, BD Biosciences) and analyzed using FlowJo version 10 (flow cytometry analysis software, BD Biosciences). The geometric mean of the eGFP and mCherry fluorescent signal for round and mCherry^+^ cells was calculated. GFP expression was normalized to mCherry signal, and drug treatments were compared to DMSO controls. The dose-dependent degradation curve was generated using locally estimated scatterplot smoothing (LOESS) regression in R. The DC_50_ values of MMH1 and MMH2 were derived using standard four-parameter log-logistic curves fitted with the ‘dr4pl (version 1.1.11)’ R package.

### Competition growth assays

K562–Cas9 cells stably expressing relevant sgRNA with BFP or RFP were mixed with wild-type control cells at a 1:9 ratio and plated at 2 × 10^5^ cells per well in a 96-well plate. Cells were dosed with DMSO, 0.1 μM JQ1, 0.33 μM TMX1 or 0.33 μM GNE11 every 3–4 d. On the same day of drug treatment, cells were split at a 1:3 ratio for maintenance and analyzed by flow cytometry to determine the percentage of BFP^+^ or RFP^+^ cells.

### UPS-targeted BRD4_BD2_ reporter CRISPR screen

The UPS-targeted CRISPR library (BISON sgRNA library; Addgene, 169942 (ref. ^13^)) targeting 713 E1, E2 and E3 ubiquitin ligases, deubiquitinases and control genes with a total of 2,852 sgRNAs was cloned into the pXPR003 vector. Viruses were produced in a T-175 format as previously described^13^. In total, 2 × 10^6^ K562–Cas9 or 293T–Cas9 BRD4_BD2_ reporter cell lines were spin infected with BISON virus at a 10% volume ratio. Transduced cells were allowed to recover and expand for 9 d and then treated with DMSO or degraders. Top (stable gate) and bottom (unstable gate) 5% of cells by eGFP/mCherry fluorescence ratios were sorted for three replicates with at least 1 × 10^5^ cells per replicate. Sorted cells were pelleted and lysed, and sgRNAs were amplified, quantified by next-generation sequencing and analyzed for enrichment in stable gate over unstable gate, representing degradation rescue.

### Genome-scale and UPS-targeted resistance CRISPR screen

The resistance screen was performed similarly to the BRD4_BD2_ reporter screen, with the following modifications. For genome-scale screens, 40 × 10^6^ K562–Cas9 cells were transduced with viruses generated from genome-wide CRISPR KO Brunello library (Addgene, 73179 (ref. ^44^)) at a 10% volume ratio. One day after infection, cells were selected with puromycin at a concentration of 2 μg ml^−1^. Seven days after infection, cells were treated with different compounds or DMSO. The cells were then cultured for 14 more days until collection, with one split every 3–4 d, at which point fresh drug was added. Cells were collected in three replicates, with 2 × 10^6^ cells per replicate, and sgRNAs were isolated and quantified as described above. Results were analyzed by comparing enrichment in the drug-treated arm over the DMSO arm, representing toxicity rescue.

### Data analysis of CRISPR screen

The CRISPR screen data analysis was performed as previously described^13^ and includes the following steps. (1) Reads per sgRNA were normalized to the total number of reads of each sample. (2) For each sgRNA, the enrichment ratio of reads in the stable versus the unstable sorted gate was calculated (for resistance screen, use drug-treated versus DMSO-treated arm), which was then used to rank sgRNAs. (3) The median enrichment ratio of each sgRNA across all sorting or treatment replicates (sgRNA media ratio) was calculated, and the fold change for each gene was determined as the median of sgRNA median ratio of the four sgRNAs targeting the gene. (4) The ranks for each sgRNA were summed for all its replicates, and the gene rank was determined as the median rank of the four sgRNAs targeting the gene. (5) The *P* values were calculated by simulating a distribution with sgRNAs that had randomly assigned ranks over 100 iterations (two-sided empirical rank-sum test statistics).

### Construction of the BRD4_BD2_ and DCAF16 alanine-scanning library

The BRD4_BD2_ and DCAF16 alanine-scanning library constructs were synthesized by GenScript. For the BRD4_BD2_ library, each amino acid of BRD4 between positions 349 and 461 was individually mutated to alanine, and each alanine was mutated to arginine. The mutant library was divided into two sub-libraries (BD2_AlaScan_1/2) and introduced into the Cilantro reporter vector. For the DCAF16 library, each amino acid of DCAF16 from positions 1 and 216 was individually mutated to alanine, and each alanine was mutated to arginine. The mutant library was divided into four sub-libraries (DCAF16_AlaScan_1/2/3/4) and introduced into the Ivy-Flag vector.

### BRD4_BD2_ alanine-scanning reporter screen

A total of 2 × 10^6^ K562–Cas9 cells were transduced with BD2_AlaScan_1 or BD2_AlaScan_2 libraries and were selected with 2 μg ml^−1^ puromycin 1 d after transduction. One week later, cells stably expressing BD2 alanine variant library were treated with DMSO or different BRD4 degraders for 16 h. After treatment, cells were sorted for stable and unstable eGFP/mCherry population, pelleted and lysed using the same method as reporter CRISPR screen. Alanine variant sequences were amplified, quantified by next-generation sequencing and analyzed for enrichment in the stable gate relative to unstable gate, representing degradation rescue.

### DCAF16 alanine-scanning reporter screen

K562–Cas9 cells with complete DCAF16 knockout were prepared as described above. The DCAF16-KO K562 cells were then transduced with BRD4_BD2_ Cilantro or SPIN4 Lavender reporter constructs that do not have puromycin selection marker. After reporter construct transduction, mCherry-positive cells were sorted to enrich K562 cells stably expressing BRD4_BD2_ or SPIN4 reporters. Next, a total of 2 × 10^6^ DCAF16-KO K562 reporter cells were transduced with DCAF16_AlaScan_1, DCAF16_AlaScan_2, DCAF16_AlaScan_3 or DCAF16_AlaScan_4 libraries and selected with puromycin. One week later, cells stably expressing DCAF16 alanine variant library were treated with DMSO or different BRD4 degraders for 16 h. After treatment, cells were sorted; sequencing samples were prepared; and data were analyzed using the same method described above.

### Data analysis of alanine-scanning reporter screen

The alanine scan data analysis was performed as previously described^45^. The analysis pipeline was similar to the above CRISPR screen with the following modifications. (1) The reads of alanine variants, instead of sgRNAs, were used to calculate enrichment ratios and ranks. (2) The read data of each sub-library of the same sorting replicates were concatenated before calculating the ratios and ranks. (3) Only one codon was used for each alanine variant, so the fold change for each alanine variant was determined as the median enrichment ratio of alanine variant across all sorting replicates, and the alanine variant rank was calculated by summing up the ranks across replicates for each alanine variant. Similarly, two-sided empirical rank-sum test was applied to calculate *P* values.

### Whole-cell proteome mass spectrometry: sample preparation

K562–Cas9 cells were treated at 0.5 μM TMX1 in duplicate, 0.5 μM JQ1 in singlicate or DMSO control in triplicate for 5 h and harvested by centrifugation before subjected to TMT quantification. In a separate run, K562–Cas9 cells were treated at 0.1 μM MMH1, 0.1 μM MMH2 in duplicate or DMSO control in quadruplicate for 5 h and harvested by centrifugation before subjected to label-free quantification. Cells were lysed by addition of lysis buffer (8 M urea, 50 mM NaCl, 50 mM 4-(2-hydroxyethyl)-1-piperazineethanesulfonic acid (EPPS) pH 8.5, protease and phosphatase inhibitors) and homogenization by bead beating (BioSpec) for three repeats of 30 s at 2,400 strokes per minute. Bradford assay was used to determine the final protein concentration in the clarified cell lysate. Then, 50 µg of protein for each sample was reduced, alkylated and precipitated using methanol–chloroform as previously described^46^, and the resulting washed precipitated protein was allowed to air dry. Precipitated protein was resuspended in 4 M urea, 50 mM HEPES pH 7.4, followed by dilution to 1 M urea with the addition of 200 mM EPPS, pH 8. Proteins were first digested with LysC (1:50, enzyme:protein) for 12 h at room temperature. The LysC digestion was diluted to 0.5 M urea with 200 mM EPPS pH 8, followed by digestion with trypsin (1:50, enzyme:protein) for 6 h at 37 °C.

### Whole-cell proteome mass spectrometry with TMT quantification

Anhydrous ACN was added to each tryptic peptide sample to a final concentration of 30%, followed by addition of tandem mass tag (TMT) reagents at a labeling ratio of 1:4 peptide:TMT label. TMT labeling occurred over a 1.5-h incubation at room temperature, followed by quenching with the addition of hydroxylamine to a final concentration of 0.3%. Each of the samples was combined using adjusted volumes and dried down in a speed vacuum, followed by desalting with C18 SPE (Sep-Pak, Waters). The sample was offline fractionated into 96 fractions by high pH reverse-phase high-performance liquid chromatography (HPLC) (Agilent, LC1260) through an aeris peptide XB-C18 column (Phenomenex) with mobile phase A containing 5% acetonitrile and 10 mM NH_4_HCO_3_ in LC−MS-grade water and mobile phase B containing 90% acetonitrile and 5 mM NH_4_HCO_3_ in LC–MS-grade water (both pH 8.0). The resulting 96 fractions were recombined in a non-contiguous manner into 24 fractions and desalted using C18 solid phase extraction plates (SOLA, Thermo Fisher Scientific), followed by subsequent mass spectrometry analysis.

Data were collected using an Orbitrap Fusion Lumos mass spectrometer (Thermo Fisher Scientific) coupled with a Proxeon EASY-nLC 1200 LC lump (Thermo Fisher Scientific). Peptides were separated on a 50-cm 75-μm-inner-diameter EasySpray ES903 microcapillary column (Thermo Fisher Scientific). Peptides were separated over a 190-min gradient of 6–27% acetonitrile in 1.0% formic acid with a flow rate of 300 nl min^−1^.

Quantification was performed using an MS3-based TMT method as described previously^47^. The data were acquired using a mass range of *m*/*z* 340–1,350, resolution 120,000, AGC target 5 × 10^5^, maximum injection time 100 ms and dynamic exclusion of 120 s for the peptide measurements in the Orbitrap. Data-dependent MS2 spectra were acquired in the ion trap with a normalized collision energy (NCE) set at 35%, AGC target set to 1.8 × 10^4^ and a maximum injection time of 120 ms. MS3 scans were acquired in the Orbitrap with HCD collision energy set to 55%, AGC target set to 2 × 10^5^, maximum injection time of 150 ms, resolution at 50,000 and with maximum synchronous precursor selection (SPS) precursors set to 10.

### LC–MS data analysis for TMT quantification

Proteome Discoverer 2.4 (Thermo Fisher Scientific) was used for raw file processing; controlling peptide and protein-level FDRs; assembling proteins from peptides; and protein quantification from peptides. The MS/MS spectra were searched against a SwissProt human database (January 2021) containing both the forward and reverse sequences. Searches were performed using a 10-ppm precursor mass tolerance, 0.6-Da fragment ion mass tolerance, tryptic peptides containing a maximum of two missed cleavages, static alkylation of cysteine (57.0215 Da), static TMT labeling of lysine residues and N-termini of peptides (229.1629) and variable oxidation of methionine (15.9949 Da). TMT reporter ion intensities were measured using a 0.003-Da window around the theoretical *m*/*z* for each reporter ion in the MS3 scan. The peptide spectral matches with poor-quality MS3 spectra were excluded from quantitation (summed signal-to-noise across channels less than 100 and precursor isolation specificity less than 0.5), and the resulting data were filtered to only include proteins with a minimum of two unique peptides quantified. Reporter ion intensities were normalized and scaled using in-house scripts in the R framework (R Core Team, 2014). Significant changes comparing the relative protein abundance between samples were assessed by two-side moderated *t*-test as implemented in the limma package within the R framework^48^.

### Whole-cell proteome mass spectrometry with label-free quantification

Sample digests were acidified with formic acid to a pH of 2–3 before desalting using C18 solid phase extraction plates (SOLA, Thermo Fisher Scientific). Desalted peptides were dried in a vacuum centrifuge and reconstituted in 0.1% formic acid for LC–MS analysis.

Data were collected using a TimsTOF Pro2 (Bruker Daltonics) coupled to a nanoElute LC pump (Bruker Daltonics) via a CaptiveSpray nano-electrospray source. Peptides were separated on a reverse-phase C18 column (25 cm × 75 μM ID, 1.6 μM, IonOpticks) containing an integrated captive spray emitter. Peptides were separated using a 50-min gradient of 2–30% buffer B (acetonitrile in 0.1% formic acid) with a flow rate of 250 nl min^−1^ and column temperature maintained at 50 °C.

DDA was performed in parallel accumulation–serial fragmentation (PASEF) mode to determine effective ion mobility windows for downstream diaPASEF data collection^49^. The ddaPASEF parameters included: 100% duty cycle using accumulation and ramp times of 50 ms each, one TIMS-MS scan and 10 PASEF ramps per acquisition cycle. The TIMS-MS survey scan was acquired between 100 *m*/*z* and 1,700 *m*/*z* and 1/k0 of 0.7–1.3 V.s/cm^2^. Precursors with 1–5 charges were selected, and those that reached an intensity threshold of 20,000 arbitrary units were actively excluded for 0.4 min. The quadrupole isolation width was set to 2 *m*/*z* for *m*/*z* < 700 and 3 *m*/*z* for *m*/*z* > 800, with the *m*/*z* between 700 *m*/*z* and 800 *m*/*z* being interpolated linearly. The TIMS elution voltages were calibrated linearly with three points (Agilent ESI-L Tuning Mix Ions; 622 *m*/*z*, 922 *m*/*z* and 1,222 *m*/*z*) to determine the reduced ion mobility coefficients (1/k0). To perform diaPASEF, the precursor distribution in the DDA *m*/*z* ion mobility plane was used to design an acquisition scheme for DIA data collection, which included two windows in each 50-ms diaPASEF scan. Data were acquired using 16 of these 25-Da precursor double window scans (creating 32 windows), which covered the diagonal scan line for doubly and triply charged precursors, with singly charged precursors able to be excluded by their position in the *m*/*z* ion mobility plane. These precursor isolation windows were defined between 400 *m*/*z* and 1,200 *m*/*z* and 1/k0 of 0.7–1.3 V.s/cm^2^.

### LC–MS data analysis for label-free quantification

The diaPASEF raw file processing and controlling peptide and protein-level FDRs, assembling proteins from peptides and protein quantification from peptides were performed using library-free analysis in DIA-NN 1.8 (ref. ^50^). Library-free mode performs an in silico digestion of a given protein sequence database alongside deep-learning-based predictions to extract the DIA precursor data into a collection of MS2 spectra. The search results are then used to generate a spectral library, which is then employed for the targeted analysis of the DIA data searched against a SwissProt human database (January 2021). Database search criteria largely followed the default settings for directDIA including tryptic with two missed cleavages, carbamidomethylation of cysteine and oxidation of methionine and precursor *q* value (FDR) cutoff of 0.01. Precursor quantification strategy was set to Robust LC (high accuracy) with RT-dependent cross-run normalization. Proteins with poor-quality data were excluded from further analysis (mean number of precursors used for quantification <2 and summed abundance across channels of <100), and proteins with missing values were imputed by random selection from a Gaussian distribution centered around the mean of the existing data and with the mean relative standard deviation of the dataset. Protein abundances were scaled using in-house scripts in the R framework (R Development Core Team, 2014). Significant changes comparing the relative protein abundance between samples were assessed by two-sided moderated *t*-test as implemented in the limma package within the R framework^48^.

### IP-MS

For IP-MS experiments, IP was performed as described above. After the washing step, samples were eluted using glycine-HCl buffer (0.2 M, pH 2.4). The IP eluates were reduced with 10 mM TCEP for 30 min at room temperature and then alkylated with 15 mM iodoacetamide for 45 min at room temperature in the dark. Alkylation was quenched by the addition of 10 mM DTT. Proteins were isolated by methanol–chloroform precipitation. The protein pellets were dried and then resuspended in 50 μl of 200 mM EPPS pH 8.0. The resuspended protein samples were digested with 2 μg of LysC overnight at room temperature, followed by the addition of 0.5 μg of trypsin for 6 h at 37 °C. Protein digests were dried, resuspended in 100 μl of 1% formic acid and desalted using 10-layer C18 stage tips before being analyzed by LC–MS.

Data were collected using an Orbitrap Exploris 480 mass spectrometer (Thermo Fisher Scientific) equipped with a FAIMS Pro Interface and coupled with a UltiMate 3000 RSLCnano System. Peptides were separated on an Aurora 25 cm × 75 μm inner diameter microcapillary column (IonOpticks) and using a 60-min gradient of 5–25% acetonitrile in 1.0% formic acid with a flow rate of 250 nl min^–1^.

Each analysis used a TopN data-dependent method. The FAIMS Pro Interface compensation voltages were set to −50 and −70. The data were acquired using a mass range of *m*/*z* 350–1,200, resolution 60,000, AGC target 3 × 10^6^, auto maximum injection time, dynamic exclusion of 15 s and charge states of 2–6. TopN 20 data-dependent MS2 spectra were acquired with a scan range starting at *m*/*z* 110, resolution 15,000, isolation window of 1.4 *m*/*z*, NCE set at 30%, AGC target 1 × 10^5^ and the automatic maximum injection time.

### LC–MS data analysis for IP-MS

Proteome Discoverer 2.4 (Thermo Fisher Scientific) was used for raw file processing; controlling peptide and protein-level FDRs; assembling proteins from peptides; and protein quantification from peptides. MS/MS spectra were searched against a UniProt human database (January 2021) with both the forward and reverse sequences as well as known contaminants, such as human keratins. Database search criteria were as follows: tryptic with two missed cleavages, a precursor mass tolerance of 10 ppm, fragment ion mass tolerance of 0.6 Da, static alkylation of cysteine (57.02146 Da) and variable oxidation of methionine (15.99491 Da). Peptides were quantified using the MS1 Intensity, and peptide abundance values were summed to yield the protein abundance values.

Resulting data were filtered to only include proteins that had a minimum of two abundance counts in at least two runs. Abundances were normalized and scaled using in-house scripts in the R framework. Missing values in the dataset were imputed by random selection from a Gaussian distribution centered around the mean of the existing data and with the mean relative standard deviation of the dataset. Significant changes comparing the relative protein abundance between samples were assessed by two-sided moderated *t*-test as implemented in the limma package within the R framework^48^. A protein was considered a ‘hit’ if it met our predetermined ‘hit’ threshold of *P* < 0.01 and fold change > 2.

### MMH2-Biotin pulldown mass spectrometry

K562–Cas9 cells (10^7^ per replicate) were lysed in 400 μl of RIPA buffer (150 mM NaCl, 5 mM EDTA, 50 mM Tris pH 7.5, 1% NP-40, 0.1% SDS, 1 cOmplete tablet (Roche Diagnostics)) with sonication (5 ×2 s at 25% amplitude). Clarified lysate was incubated with 1 μM compound or DMSO and 50 μl of pre-washed Mag-Strep ‘type3’ Strep-Tactin beads (IBA Lifesciences) for 6 h at 4 °C on a rotator. Beads were magnetically removed and washed three times with RIPA buffer and three times with 50 mM Tris (pH 7.5). Proteins were eluted with two 20-min incubations in 50 μl of 0.5 M NaOH and neutralized with 1 M Tris.

Reduction, alkylation and methanol–chloroform precipitation were performed as described above. Proteins were digested overnight at 37 °C with LysC (2 μg) and trypsin (0.5 μg). Digests were acidified with formic acid and desalted over C18 solid phase extraction plates.

Peptides were analyzed on an UltiMate 3000 RSLCnano system coupled to an Eclipse mass spectrometer (Thermo Fisher Scientific). Peptides were separated across a 65-min gradient of 6–30% acetonitrile in 0.1% formic acid over a 50-cm C18 column (ES903, Thermo Fisher Scientific) and electrosprayed (1.9 kV, 300 °C) into the mass spectrometer with an EasySpray ion source (Thermo Fisher Scientific). Precursor ion scans (375–1,325 *m*/*z*) were obtained in the Orbitrap at 120,000 resolution in profile (RF lens % = 30, Max IT = 200 ms). MS2 scans were acquired in the Orbitrap after HCD fragmentation (35% NCE) in data-dependent mode with the following acquisition parameters: 0.7 *m/z* isolation window, 30,000 resolution, standard AGC target, dynamic Max IT, dynamic exclusion (*n* = 2 in 15 s, 30-s exclusion) and cycle time =3 s.

### LC–MS data analysis for MMH2-Biotin pulldown mass spectrometry

Raw file processing, FDR filtering and protein quantification from peptides were conducted with Proteome Discoverer 2.4 (Thermo Fisher Scientific), as described above. Resulting data were filtered to include proteins that had a minimum of three abundance counts in at least three replicates. Normalization, imputation and statistical analysis were performed using in-house R scripts, as described above.

### Protein expression and purification

The human wild-type and mutant versions of DCAF16 (UniProt entry Q9NXF7, full length), DDB1ΔB (UniProt entry Q16531, residues 396–705 replaced with GNGNSG linker) and DDA1 (UniProt entry Q9BW61, full length) were cloned in pAC-derived vectors^51^, and recombinant proteins were expressed as N-terminal His_6_ (DDA1), StrepII-Avi (DCAF16) or His6-3C-Spy (DDB1ΔB) fusions in *Trichoplusia ni* High Five insect cells (Thermo Fisher Scientific, 85502) using the baculovirus expression system (Gibco). In brief, expression plasmids were transfected into *Spodoptera frugiperda* (Sf9) cells (Expression Systems, 94-001F) at a density of 0.9 × 10^6^ cells per milliliter grown in ESF 921 medium (Expression Systems) to generate baculovirus, and this was followed by two rounds of infection in Sf9 cells to increase viral titer. For recombinant protein expression, High Five cells grown in Sf-900 II SFM media (Gibco) at a density of 2.0 × 10^6^ cells per milliliter were infected with baculovirus at 1.5% v/v ratio. After 40 h of expression at 27 °C, High Five cells were collected by centrifugation for 15 min at 3,500*g*. For purification of StrepII or His_6_-tagged proteins, pelleted cells were resuspended in lysis buffer containing 50 mM Tris (hydroxymethyl) aminomethane hydrochloride (Tris-HCl) pH 8.0, 200 mM NaCl, 2 mM Tris (2-carboxyethyl) phosphine (TCEP), 1 mM phenylmethylsulfonyl fluoride (PMSF) and protease inhibitors, and the cell pellets were lysed by sonication. After ultracentrifugation (1 h, 185,511*g*), the soluble fraction was passed over the appropriate affinity resin of Strep-Tactin XT Superflow (IBA) or Ni Sepharose 6 Fast Flow affinity resin (GE Healthcare), eluted with wash buffer (50 mM Tris-HCl pH 8.0, 200 mM NaCl, 1 mM TCEP) supplemented with 50 mM d-Biotin (IBA) or increasing imidazole concentrations (100–500 mM) (Fisher Chemical), respectively. The affinity-purified proteins were then applied to an ion exchange column (POROS 50HQ, Thermo Fisher Scientific) and eluted in 50 mM Tris-HCl pH 8.5 and 2 mM TCEP by a linear salt gradient (from 50 mM to 800 mM NaCl). Purified DCAF16 was dephosphorylated with lambda-phosphatase (New England Biolabs) at 4 °C overnight. The DCAF16 complex and BRD4_BD2_ were cleaved with tobacco etch virus (TEV) protease (1:25) at 4 °C overnight. All proteins were then subjected to SEC on a Superdex 200 Increase 10/300 (GE Healthcare) in 50 mM 4-(2-hydroxyethyl)-1-piperazineethanesulfonic acid (HEPES) pH 7.4 or pH 8.0, 200 mM NaCl and 2 mM TCEP. Peak fractions were pooled, concentrated, flash frozen in liquid nitrogen and stored at −80 °C.

The human wild-type BRD4_BD1_ and BRD4_BD2_ (UniProt entry O60885, residues 75–147 and 368–440) were subcloned into *E. coli* pET100/D-TOPO vector with N-terminal His_6_-Avi fusions and expressed in *E. coli* BL21-DE3 Rosetta cells using standard protocols. Biotinylation of BRD4_BD1_ and BRD4_BD2_ was done as previously described^47^. The human wild-type BRD4_tandem_ (UniProt entry O60885, residues 75–440) and human wild-type GAK kinase domain (UniProt entry O14976, residues 14–351) were subcloned into a pNIC-Bio2 vector with N-terminal His10 fusion and expressed in *E. coli* LOBSTR (Kerafast, EC1002) cells using standard protocols. Cultures were grown at 37 °C to OD_600_ = ~0.6 and induced with 0.35 mM isopropyl ß-d-1-thiogalactopyranoside (IPTG). Temperature was decreased to 18 °C, and the protein was expressed overnight. Cell pellets were resuspended in lysis buffer (50 mM HEPES/KOH pH 7.4, 500 mM NaCl, 20 mM imidazole, 5% glycerol, 1 mM TCEP) and lysed using sonication, followed by clearance using ultracentrifugation (1 h, 180,000*g*). Cleared lysates were applied to high-affinity Ni-charged resin (GenScript, L00223) and eluted with increasing imidazole concentrations (50–750 mM). Protein-containing fractions were pooled, diluted to approximately 300 mM NaCl and applied to a POROS 50HQ. Impurities bound to the ion exchange column, and GAK was collected from the flow-through. The sample was buffer exchanged using PD10 columns (Thermo Fisher Scientific), and the His tag was cleaved using TEV protease (1:25) overnight at 4 °C. The sample was passed over a Ni column to remove TEV protease and cleaved His-tags, and polishing was performed by SEC on a Superdex75 (Cytivia) column. The final protein was concentrated using 30,000 molecular weight cutoff (MWCO) centrifugal concentrators.

### BODIPY-FL-Spycatcher labeling of DCAF16–DDB1ΔB

Purified StrepII-Avi-DCAF16 + His_6_-3C-Spy-DDB1ΔB was incubated overnight at 4 °C with BODIPY-FL-labeled SpyCatcherS50C protein at stoichiometric ratio. Protein was concentrated and loaded on the Enrich SEC 650 10/300 (Bio-Rad) size exclusion column, and the labeling was monitored with absorption at 280 nm and 490 nm. The protein peak corresponding to the labeled protein was pooled, concentrated by ultrafiltration (Millipore) and flash frozen.

### DDB1–DCAF16–BRD4_BD_ TR-FRET

Titrations of compounds to induce the DCAF16–BRD4_BD_ complex were carried out by mixing 100 nM biotinylated BRD4_BD1_ or BRD4_BD2_, 500 nM BODIPY-FL-labeled DDB1ΔB–DCAF16 variants and 2 nM terbium-coupled streptavidin (Invitrogen) in an assay buffer containing 50 mM HEPES pH 8.0, 200 mM NaCl, 0.1% Pluronic F-68 solution (Sigma-Aldrich), 0.5% BSA (w/v) and 1 mM TCEP. After dispensing the assay mixture (15 μl volume), increasing concentrations of compounds were dispensed in a 384-well microplate (Corning, 4514) using a D300e Digital Dispenser (HP) normalized to 1% DMSO. After excitation of terbium fluorescence at 337 nm, emission at 490 nm (terbium) and 520 nm (BODIPY FL) were recorded with a 70-μs delay over 600 μs to reduce background fluorescence, and the reaction was followed over 60 cycles of each data point using a PHERAstar FS microplate reader (BMG Labtech). The TR-FRET signal of each data point was extracted by calculating the 520/490-nm ratio. The dose-dependent TR-FRET curve was generated using LOESS regression in R.

### Intact protein mass spectrometry

Before intact mass analysis, 15 µM recombinant human DDB1ΔB–DCAF16 or GAK was incubated with 50 µM DMSO or compounds (TMX1, KB02-JQ1, MMH1 and MMH2 for DDB1ΔB-DCAF16 and TMX1 and MMH2 for GAK), with and without the presence of 25 µM recombinant human BRD4_BD2_ for 16 h at 4 °C. For GNE11, recombinant proteins were incubated with drug at room temperature for 16 h. For IBG1, recombinant proteins were incubated with drug with and without the presence of recombinant human BRD4_tandem_ for 16 h at 4 °C. For the DDB1ΔB–DCAF16 samples, intact mass analysis was performed similarly to a previously described protocol^52^ with modifications. In brief, drug-treated proteins were injected on a self-packed column (6 cm POROS 50R2 packed in 0.5 mm I.D. tubing), desalted for 4 min and then eluted to an LTQ ion trap mass spectrometer (Thermo Fisher Scientific) using an HPLC gradient (0–100% B in 20 min, A = 0.1 M acetic acid, B = 0.1 M acetic acid in acetonitrile, ESI spray voltage = 5 kV). The mass spectrometer acquired full scan mass spectra (*m*/*z* 300–2,000) in profile mode. Mass spectra were deconvoluted using MagTran (version 1.03 b2)^53^. For the GAK samples and IBG1-treated BRD4_tandem_ samples, the drug-treated proteins were desalted over C4 resin and injected on a U3000 RSLC fitted with a MAbPac RP column (Thermo Fisher Scientific, ES907; 150 mm × 15 cm column packed with 4 mm, 1,500 Å resin). Proteins were eluted with a 5–50% gradient of acetonitrile in 1% formic acid over 15 min and electrosprayed (1.9 kV) into an Orbitrap Eclipse mass spectrometer (Thermo Fisher Scientific). Full scan mass spectra (*m*/*z* 600–2,000) were acquired in profile mode. Mass spectra were deconvoluted using UniDec (version 6.0.4)^54^. Labeling efficiency for all intact mass analysis was calculated from zero charge mass spectra using peak heights according to [peak height labeled protein] / [peak height labeled protein + peak height unlabeled protein] × 100%.

### LC–MS/MS analysis for GAK covalent labeling site identification

Proteins in the compound labeling reaction were denatured in 8 M urea and reduced in 10 mM TCEP for 30 min at room temperature. Cysteine residues were alkylated with 15 mM iodoacetamide for 45 min in the dark and quenched with 10 mM DTT. Urea was diluted to 2 M before proteolytic digest with LysC and trypsin at 37 °C overnight. Digests were quenched to 1% formic acid and desalted over SOLAμ HRP elution plates (Thermo Fisher Scientific).

Peptides were analyzed on an UltiMate 3000 RSLCnano system coupled to a Fusion Lumos mass spectrometer (Thermo Fisher Scientific). Peptides were separated across a 70-min gradient of 6–30% acetonitrile in 1% formic acid over a 50-cm C18 column (ES803A, Thermo Fisher Scientific) and electrosprayed (1.9 kV, 305 °C) into the mass spectrometer with an EasySpray ion source (Thermo Fisher Scientific). Precursor ion scans (375–1,325 *m*/*z*) were obtained in the Orbitrap at 120,000 resolution in profile. Fragment ion scans of peptide ions in a targeted inclusion list consisting of modified and unmodified peptides containing GAK C87 with 0–2 missed cleavages were acquired in the Orbitrap after HCD fragmentation (30% NCE, 0.5 *m/z* isolation, 30,000 resolution). LC–MS/MS data were also acquired in data-dependent mode for an unbiased acquisition. Raw file processing and FDR filtering (target/decoy) were achieved with Proteome Discoverer 2.5 (Thermo Fisher Scientific). Raw data were searched against the GAK engineered sequence and an *E. coli* protein database (UniProt) using SEQUEST, permitting a mass tolerance of ±10 ppm, two missed cleavages and the following modifications: methionine oxidation, serine/threonine/tyrosine phosphorylation, cysteine carbamidomethylation and cysteine-bound MMH2. Spectra of the MMH2-bound peptide were manually confirmed and annotated.

### EM sample preparation and data collection

The ternary complex was incubated at room temperature for 30 min at 15 μM DCAF16–DDB1ΔB–DDA1 complex, 25 μM BRD4_BD2_ and 50 μM MMH2 before loading on a Superdex 200 Increase 10/300 SEC column. After SEC, the purified DCAF16 complex was incubated with an extra 1.2× molar excess of purified BRD4_BD2_ for 30 min at 4 °C and then mixed with 0.011% LMNG right before preparation of cryo-EM grids. Glow-discharged Quantifoil UltrAuFoil 0.6/1.0 grids were prepared using a Leica EM-GP, operated at 10 °C and 90% relative humidity. Then, 4 μl of sample (1.25 mg ml^−1^) was applied, incubated on the grid for 10 s and blotted for 3 s before vitrification. Grids were imaged in a Titan Krios equipped with a Gatan Quantum Image filter (20 eV slit width) and a post-GIF Gatan K3 direct electron detector. Next, 17,118 movies were acquired at 300 kV at a nominal magnification of ×105,000 in counting mode with a pixel size of 0.83 Å per pixel using SerialEM version 4.0.5 (ref. ^55^). One movie (40 frames each) was acquired per hole with nine holes per stage position (resulting in nine image acquisition groups), in a defocus range from −0.8 μm to −2.0 μm over an exposure time of 2.30 s and a total dose of 50.27 e/Å^2^.

### EM data processing and model building

All processing was performed in cryoSPARC version 3.3.2 (ref. ^56^). Then, 17,118 movies were corrected for beam-induced motion, and contrast transfer function was estimated on-the-fly in cryoSPARC live. Next, 14,452,363 particles were extracted (at 1.58 Å per pixel) from 15,448 curated micrographs after Topaz version 0.2.5a particle picking^57^. The extracted particles were split into two batches and sent through two rounds of two-dimensional (2D) classification to remove mispicks and DDB1-only classes. The resulting particles were combined and further cleaned by an additional round of 2D classification. The remaining 4,795,088 particles were classified by three-dimensional (3D) variability^58^ in clustering mode (eight clusters). Particles from three clusters (1,433,050) with most pronounced density for BRD4_BD2_ were combined and re-extracted at 0.89 Å per pixel, and a final homogeneous refinement was followed by local refinement using a mask encompassing the whole particle. The final reconstruction reached a resolution of 2.2 Å, based on the Fourier shell correlation (FSC) 0.143 threshold criterion^59,60^. This map, sharpened with a *B* value of −74.4 Å^2^, as well as a map post-processed using DeepEMhancer version 0.16 (ref. ^43^), were used for model building in Coot version 0.9.8 (ref. ^61^). Models for DDB1, DDA1 (PDB: 6Q0R (ref. ^62^)) and BRD4_BD2_ (PDB: 6VIX (ref. ^63^)) were first fit as rigid bodies in ChimeraX version 1.4 (ref. ^64^), relaxed into the density using ISOLDE version 1.3 (ref. ^65^) and then adjusted manually in Coot. The model for DCAF16 was built de novo. A component dictionary for the MMH2 compound and a link dictionary for MMH2 linked to cysteine were generated using AceDRG version 5.8.0091 (refs. ^66,67^). The compound was linked to Cys58, and the model was refined iteratively in Refmac5 version 5.8.0091 (ref. ^68^) and phenix.real_space_refine version 1.19.2-4158 (refs. ^69,70^). The resulting model was deposited in the PDB under accession code 8G46. The final map was deposited as main map in the Electron Microscopy Data Bank (EMDB) (EMD-29714) with the map from DeepEMhancer as an additional map. Interface areas were calculated using PDBePisa version 1.52 (ref. ^30^); structural similarity searches were conducted using PDBeFold^71^; and all figures with models and density were generated in ChimeraX. The map and model resolution ranges are given based on the 0–75% percentile in local resolution histograms^72^. Directional resolution was calculated using 3DFSC version 3.0 (ref. ^73^). Structural biology applications used in this project were compiled and configured by SBGrid^74^.

### BRD4_BD1_ and BRD4_BD2_ AlphaScreen assays

The AlphaScreen assays were performed with minor modifications from the manufacturer’s protocol (PerkinElmer). All reagents were diluted in AlphaScreen buffer (50 mM HEPES, 150 mM NaCl, 0.01 % v/v Tween 20, 0.1% w/v BSA, pH 7.4). After addition of the Alpha beads to the master solutions, all subsequent steps were performed under low-light conditions. A 2× solution of components with final concentrations of His-BRD4_BD1_, His-BRD4_BD2_ at 20 nM, Ni-coated acceptor bead at 10 μg ml^−1^ and biotinylated-JQ1 at 10 nM was added in 10 μl to 384-well plates (AlphaPlate-384, PerkinElmer). Plates were spun down at 1,000 r.p.m. A 10-point serial dilution of compounds in DMSO was prepared at 200× of the final concentration. Compound (100 nl) from these stock plates was added by pin transfer using a Janus Workstation (PerkinElmer). A 2× solution of streptavidin-coated donor beads with a final concentration of 10 μg ml^−1^ was added in a 10-μl volume. The plates were spun down again at 1,000 r.p.m. and sealed with foil to prevent light exposure and evaporation. The plates were then incubated at room temperature for 1 h and read on an EnVision 2104 Multilabel Plate Reader (PerkinElmer) using the manufacturer’s protocol. After normalization to DMSO-treated negative control wells, the dose-dependent activity inhibition curve was generated using standard four-parameter log-logistic curves fitted with the ‘dr4pl (version 1.1.11)’ R package.

### Reporting summary

Further information on research design is available in the Nature Portfolio Reporting Summary linked to this article.

## Online content

Any methods, additional references, Nature Portfolio reporting summaries, source data, extended data, supplementary information, acknowledgements, peer review information; details of author contributions and competing interests; and statements of data and code availability are available at 10.1038/s41589-024-01668-4.

## Supplementary information

Supplementary Information Supplementary Figs. 1–9, Supplementary Table 1 and Supplementary Notes 1-2.

Reporting Summary

Supplementary Code 1.

Supplementary Table 1 Coding sequences of DNA constructs used in this study.

Supplementary Table 2 Mammalian cell line authentication results.

Supplementary Data 1 Source Data Supplementary Fig. 1 Raw data for CRISPR screens.

Supplementary Data 2 Source Data Supplementary Fig. 2 Raw data for CRISPR screens and flow.

Supplementary Data 3 Source Data Supplementary Fig. 4 Raw data for alanine-scanning screens.

Supplementary Data 4 Source Data Supplementary Fig. 6 Raw data for TR-FRET.

Supplementary Data 5 Source Data Supplementary Fig. 7 Raw data for alanine-scanning screens.

## Source data

Source Data Fig. 1 Uncropped western blot.

Source Data Fig. 1 (Table) Raw data for proteomics and CRISPR screens.

Source Data Fig. 2 Uncropped western blot.

Source Data Fig. 2 (Table) Raw data for TR-FRET.

Source Data Fig. 4 Uncropped western blot.

Source Data Fig. 4 (Table) Raw data for alanine-scanning screens and TR-FRET.

Source Data Fig. 5 Uncropped western blot.

Source Data Fig. 5 (Table) Raw data for alanine-scanning screens and flow.

Source Data Fig. 6 Uncropped western blot and raw data for intact MS.

Source Data Extended Data Fig. 1 Uncropped western blot.

Source Data Extended Data Fig. 1 (Table) Raw data for flow and proteomics.

Source Data Extended Data Fig. 2 (Table) Raw data for proteomics and TR-FRET.

Source Data Extended Data Fig. 3 Uncropped western blot.

Source Data Extended Data Fig. 3 (Table) Raw data for flow and TR-FRET.

Source Data Extended Data Fig. 4 (Table) Raw data for flow and proteomics.

Source Data Extended Data Fig. 5 (Table) Raw data for proteomics, flow and TR-FRET.

Source Data Extended Data Fig. 7 (Table) Uncropped western blot.

Source Data Extended Data Fig. 7 Raw data for flow.

Source Data Extended Data Fig. 8 Uncropped western blot and raw data for intact MS.

Source Data Extended Data Fig. 8 (Table) Raw Data for flow.

Source Data Extended Data Fig. 9 (Table) Raw data for alanine-scanning screens and AlphaScreen.

Source Data Extended Data Fig. 10 Uncropped western blot and raw data for intact MS.

## Data Availability

Cryo-EM maps and coordinates have been deposited in the Electron Microscopy Data Bank and the Protein Data Bank, under accession codes EMD-29714 and 8G46, respectively. Raw data files of whole-cell proteome mass spectrometry, IP-MS and Biotin pulldown mass spectrometry in this study have been deposited in the PRIDE Archive, including PXD047137, PXD047138, PXD047141 and PXD051457. Intact mass spectrometry raw data related to Figs. 2b and 4e and Extended Data Figs. 2d,e, 4e,f and 7f are available for free download at ftp://massive.ucsd.edu/MSV000093731. Synthetic procedures of JQ1-derived compounds, schematics of sorting strategies and deep sequencing results for DCAF16-knockout clones are provided in the Supplementary Information. Coding sequences of the DNA constructs used in this study and mammalian cell line authentication results are provided as supplementary tables. Source data are provided with this paper.

## Code availability

Codes used to identify hits and generate volcano plots in screens, as well as codes used to generate dose−response curves and correlation plots, are provided as Supplementary Code.
