# Supplementary material for: Template-assisted covalent modification underlies activity of covalent molecular glues
Source: Nat Chem Biol. 2024 Jul 29;20(12):1640–9. doi: 10.1038/s41589-024-01668-4 (PMC11582070; doi:10.1038/s41589-024-01668-4)
Supplement: Supplementary file 1 — Supplementary Figs. 1–9, Supplementary Table 1 and Supplementary Notes 1-2. [file 41589_2024_1668_MOESM1_ESM.pdf]

nature chemical biology

Supplementary information https://doi.org/10.1038/s41589-024-01668-4

Template-assisted covalent modification underlies activity of covalent molecular glues

In the format provided by the authors and unedited

Supplementary Information includes the following sections:

- Supplementary Fig. 1-9
- Supplementary Table 1
- Supplementary Note: Synthesis of compounds, characterization, and spectra
- Supplementary Note: Deep sequencing results for DCAF16 knockout clones

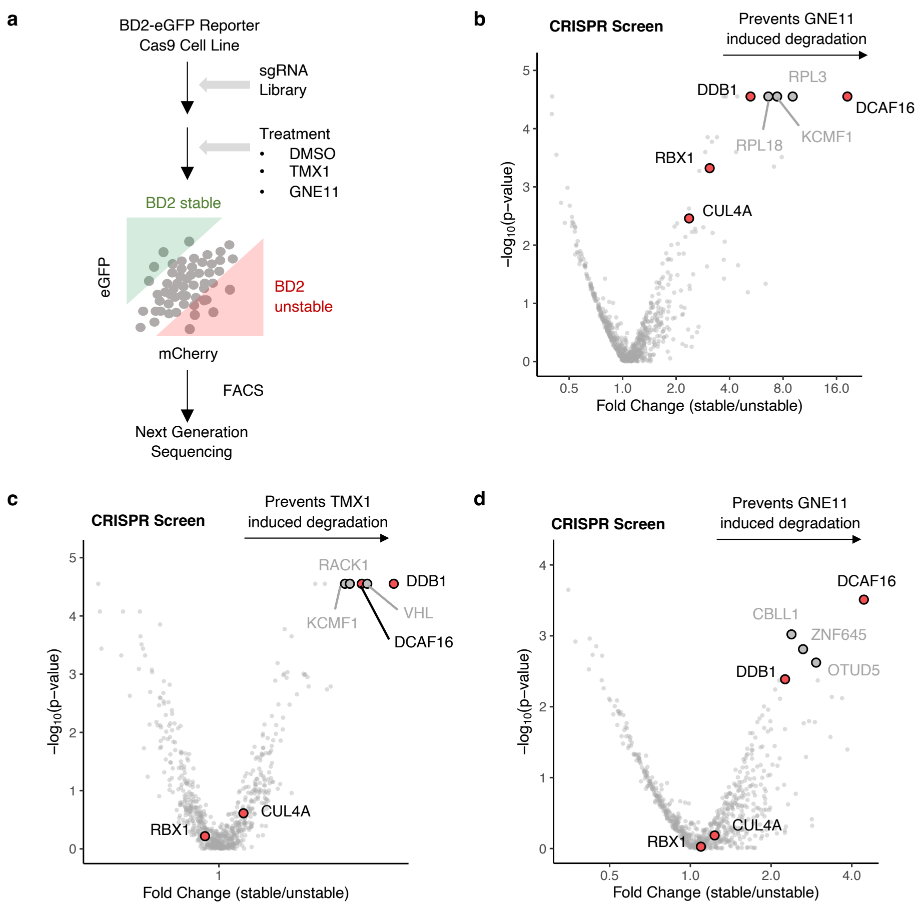

Supplementary Fig. 1: UPS-targeted BRD4$_{BD2}$ reporter CRISPR screen for JQ1-derived compounds. a. Schematic of the CRISPR degradation screen for BRD4$_{BD2}$ stability. **b.** UPS-focused CRISPR degradation screen for BRD4$_{BD2}$-eGFP stability in K562-Cas9 cells treated with GNE11 at 1 μM for 16 h (n=2). Statistical analysis was performed using a two-sided empirical rank-sum test. **c.** UPS-focused CRISPR degradation screen for BRD4$_{BD2}$-eGFP stability in 293T-Cas9 cells treated with TMX1 at 1 μM for 16 h (n=2). Statistical analysis was performed using a two-sided empirical rank-sum test. **d.** UPS-focused CRISPR degradation screen for BRD4$_{BD2}$-eGFP stability in 293T-Cas9 cells treated with GNE11 at 1 μM for 16 h (n=2). Statistical analysis was performed using a two-sided empirical rank-sum test.

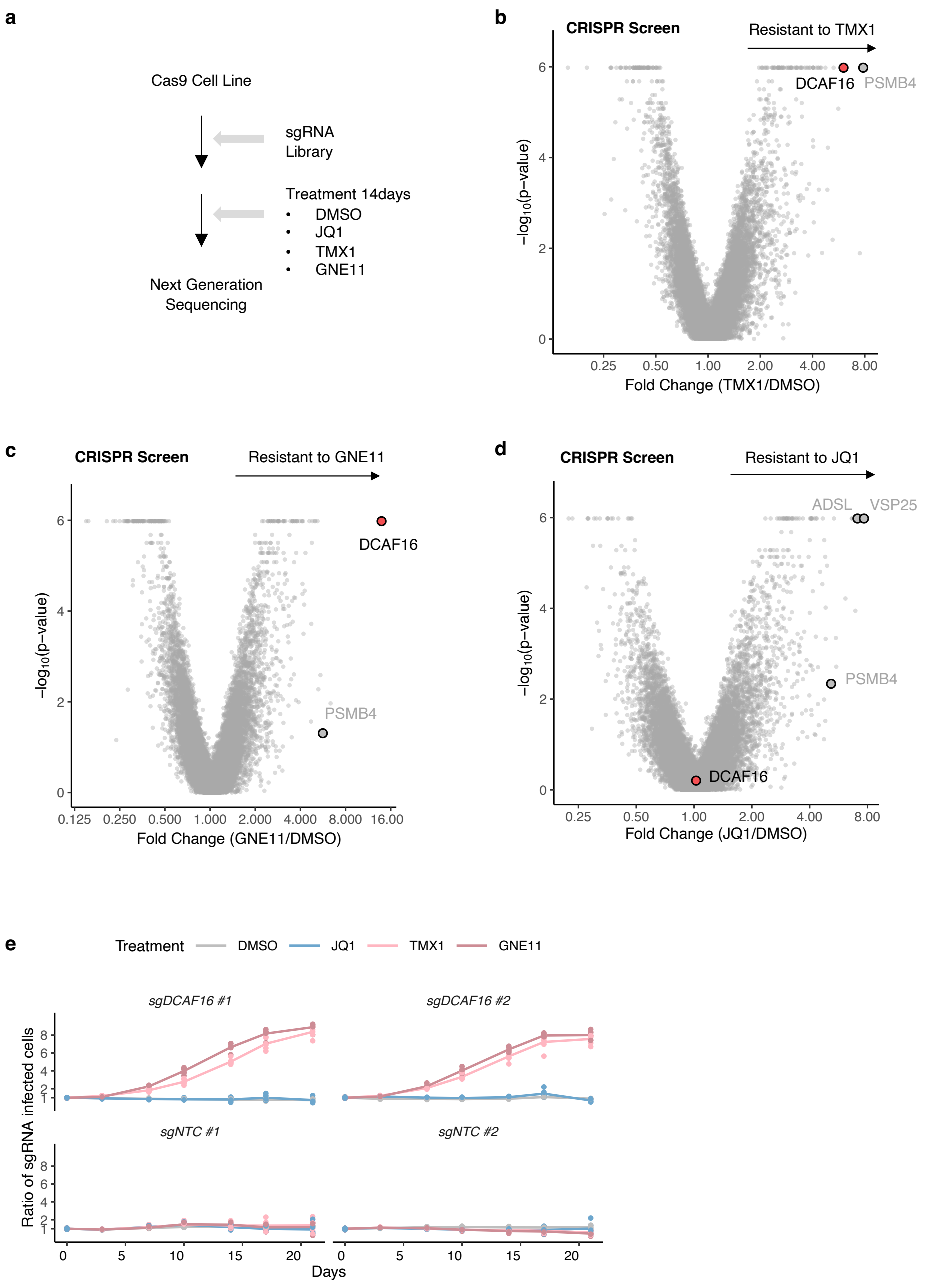

Supplementary Fig. 2: Genome-scale resistance CRISPR screen for JQ1-derived compounds. a. Schematic of the CRISPR resistance screen. **b.** Genome-wide CRISPR resistance screen in K562-Cas9 cells treated with TMX1 at 0.1 µM (n=3) or DMSO (n=3) for 14 days. Statistical analysis was performed using a two-sided empirical rank-sum test. **c.** Genome-wide CRISPR resistance screen in K562-Cas9 cells treated with GNE11 at 0.1 µM (n=3) or DMSO (n=3) for 14 days. Statistical analysis was performed using a two-sided empirical rank-sum test. **d.** Genome-wide CRISPR resistance screen in K562-Cas9 cells treated with JQ1 at 0.1 µM (n=3) or DMSO (n=3) for 14 days. Statistical analysis was performed using a two-sided empirical rank-sum test. **e.** Flow cytometry-based competitive growth assay of K562-Cas9 cells expressing BFP- or RFP-tagged sgRNAs against DCAF16 and non-targeting control (NTC) treated with DMSO, JQ1 at 0.1 µM, TMX1 at 0.1 µM, or GNE11 at 0.1 µM for increasing time points (n=6).

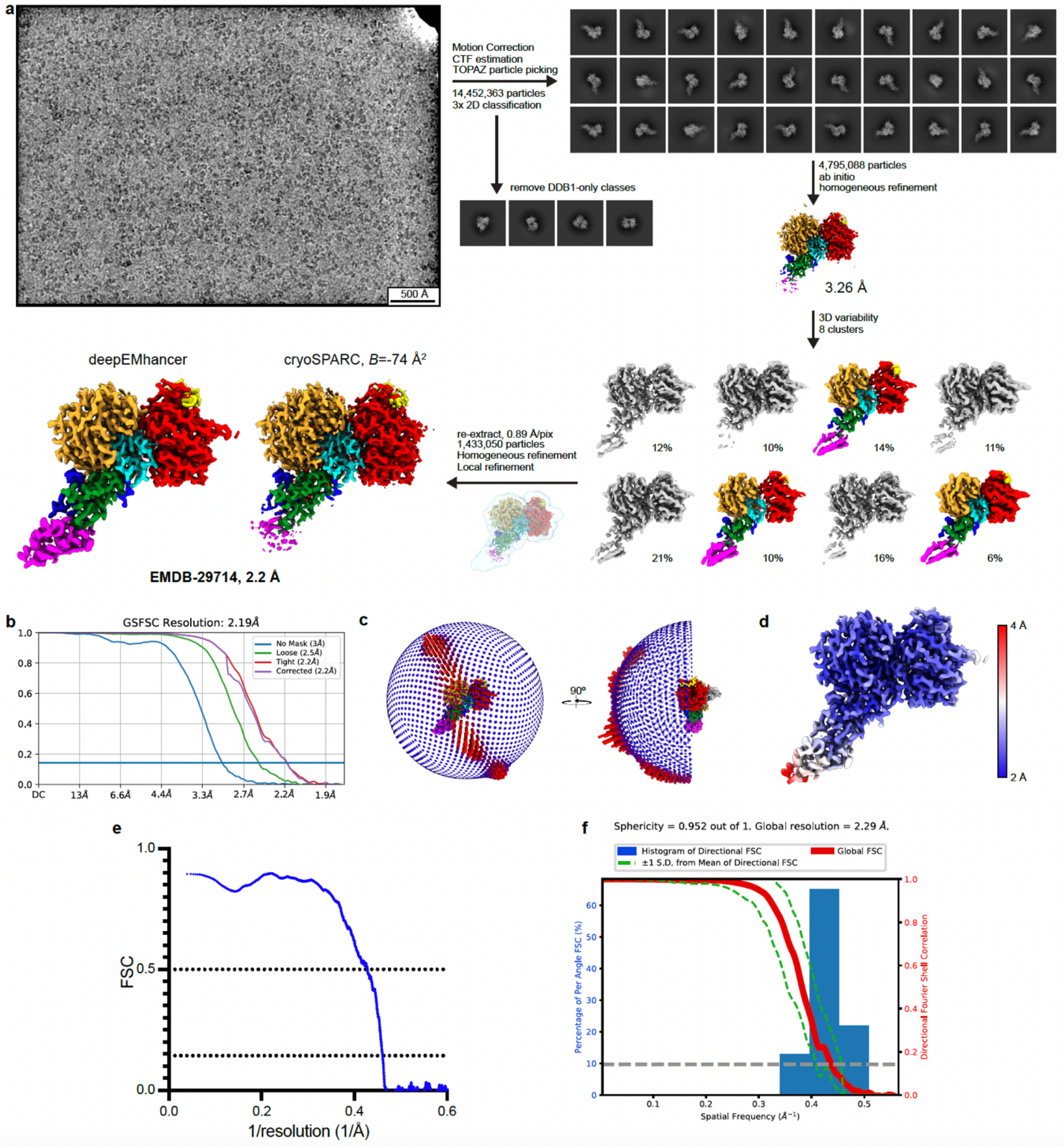

Supplementary Fig. 3: Cryo-EM processing workflow of DDB1ΔB-DDA1-DCAF16 in complex with BRD4$_{BD2}$ and MMH2. a. Overview of processing workflow for the DDB1ΔB-DDA1-DCAF16-BRD4$_{BD2}$-MMH2 dataset, from raw micrographs (low pass-filtered to 5 Å) to final maps. All steps performed in cryoSPARC[56]. Particles belonging to colored volumes were taken into the subsequent steps. Maps here and in following panels (unless noted otherwise) are contoured at 0.3 (clusters), 0.743 (initial consensus), 0.6 (final from cryoSPARC), 0.2 (final from deepEMhancer). **b.** FSC plot. **c.** Viewing distribution for the final reconstruction. **d.** Local resolution mapped onto final map. **e.** Model-to-map FSC, dotted lines indicate FSC=0.5 and FSC=0.143. **f.** 3D FSC plot and directional resolution histogram.

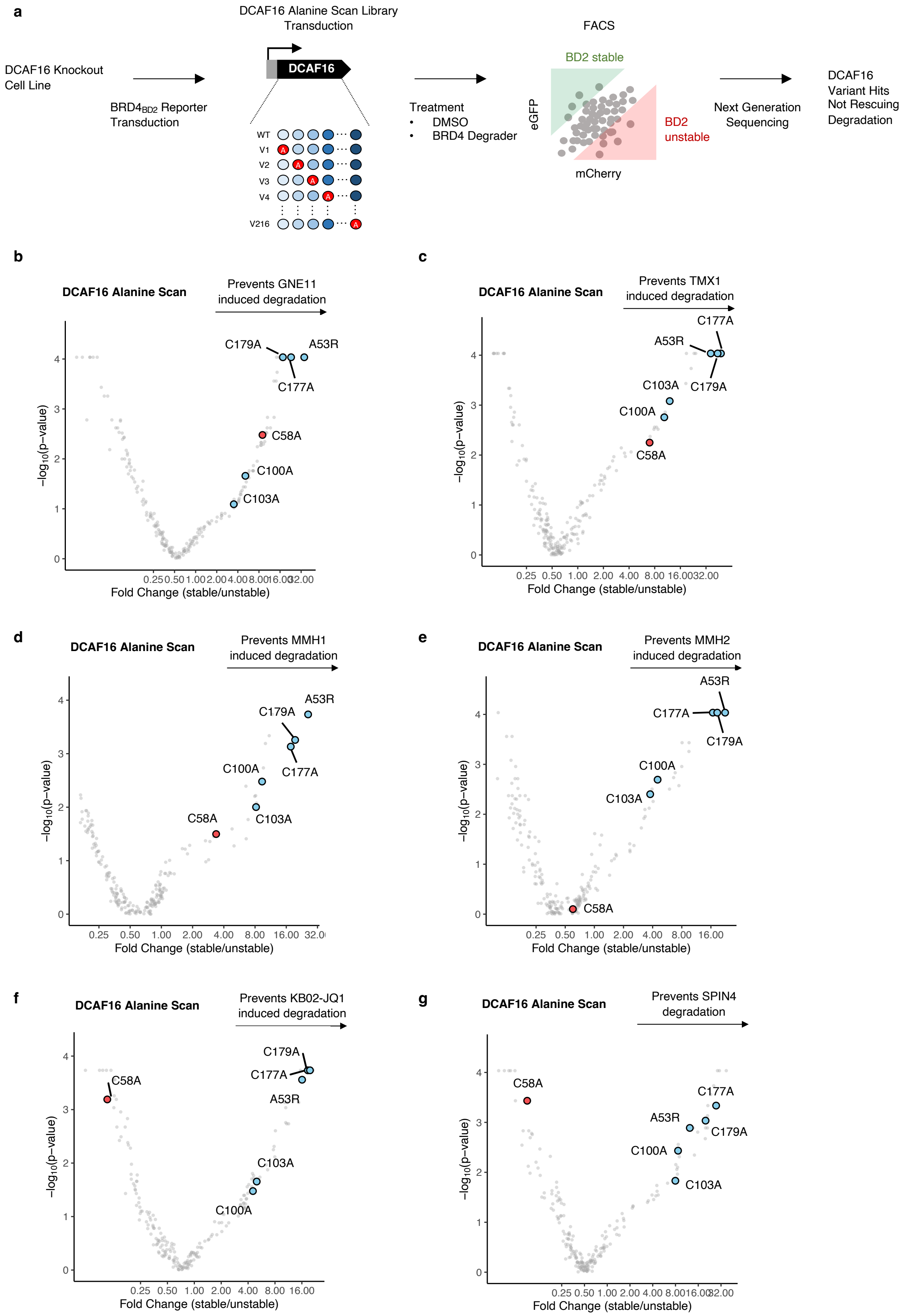

Supplementary Fig. 4: DCAF16 alanine-scanning reporter screens. a. Schematic of DCAF16 alanine mutagenesis screen for $BRD4_{BD2}$-eGFP degradation in DCAF16 knockout K562 cells. **b.** DCAF16 alanine mutagenesis screen for $BRD4_{BD2}$-eGFP stability in K562

cells treated with GNE11 at 1 µM for 16 h (n=3). Statistical analysis was performed using a two-sided empirical rank-sum test. **c.** DCAF16 alanine mutagenesis screen for $BRD4_{BD2}$-eGFP stability in K562 cells treated with TMX1 at 1 µM for 16 h (n=3). Statistical analysis was performed using a two-sided empirical rank-sum test. **d.** DCAF16 alanine mutagenesis screen for $BRD4_{BD2}$-eGFP stability in K562 cells treated with MMH1 at 0.1 µM for 16 h (n=3). Statistical analysis was performed using a two-sided empirical rank-sum test. **e.** DCAF16 alanine mutagenesis screen for $BRD4_{BD2}$-eGFP stability in K562 cells treated with MMH2 at 0.1 µM for 16 h (n=3). Statistical analysis was performed using a two-sided empirical rank-sum test. **f.** DCAF16 alanine mutagenesis screen for $BRD4_{BD2}$-eGFP stability in K562 cells treated with KB02-JQ1 at 10 µM for 16 h (n=3). Statistical analysis was performed using a two-sided empirical rank-sum test. **g.** DCAF16 alanine mutagenesis screen for eGFP-SPIN4 stability in K562 cells (n=3). Statistical analysis was performed using a two-sided empirical rank-sum test.

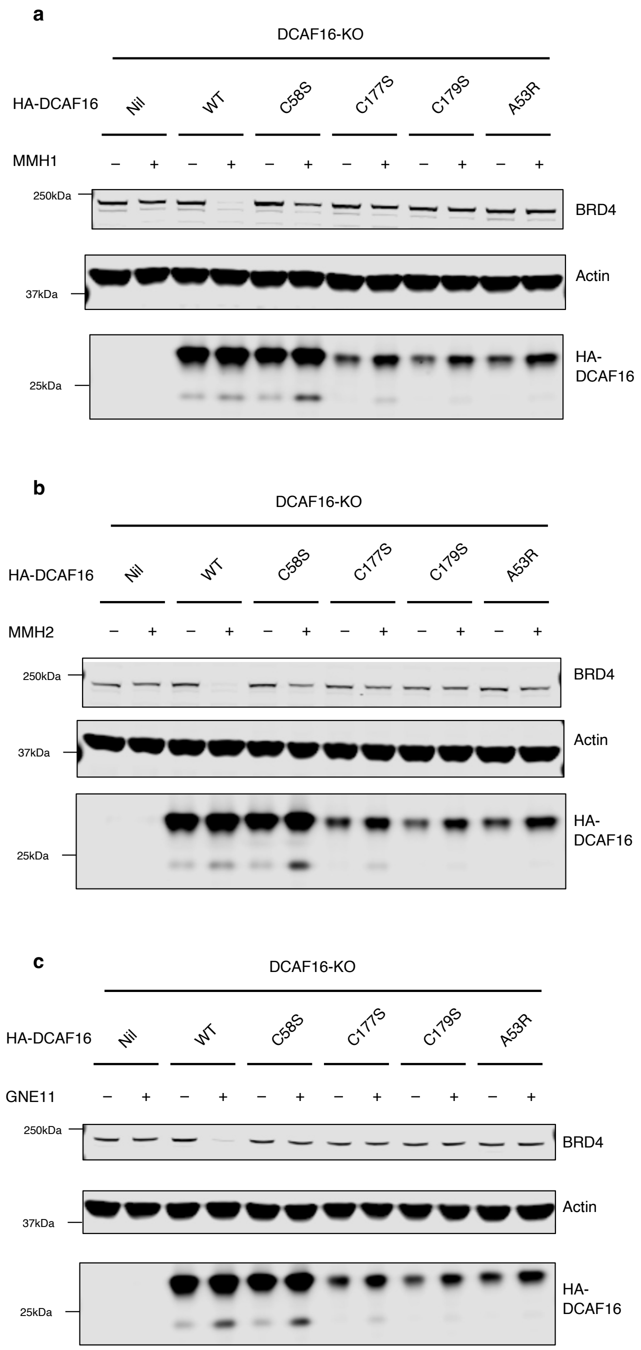

Supplementary Fig. 5: Western blot validation of representative DCAF16 mutants. a. Western blots of BRD4 degradation in DCAF16 knockout K562 cells that were transduced with indicated HA-DCAF16 mutants and treated with DMSO or MMH1 at 0.1 µM for 16 h. **b.** Western blots of BRD4 degradation in DCAF16 knockout K562 cells that were transduced with indicated HA-DCAF16 mutants and treated with DMSO or MMH2 at 0.1 µM for 16 h. **c.** Western blots of BRD4 degradation in DCAF16 knockout K562 cells that were transduced with indicated HA-DCAF16 mutants and treated with DMSO or GNE11 at 1 µM for 16 h. All western blot data are representative of two independent measurements (For uncropped western blots, see Supplementary Fig. 8).

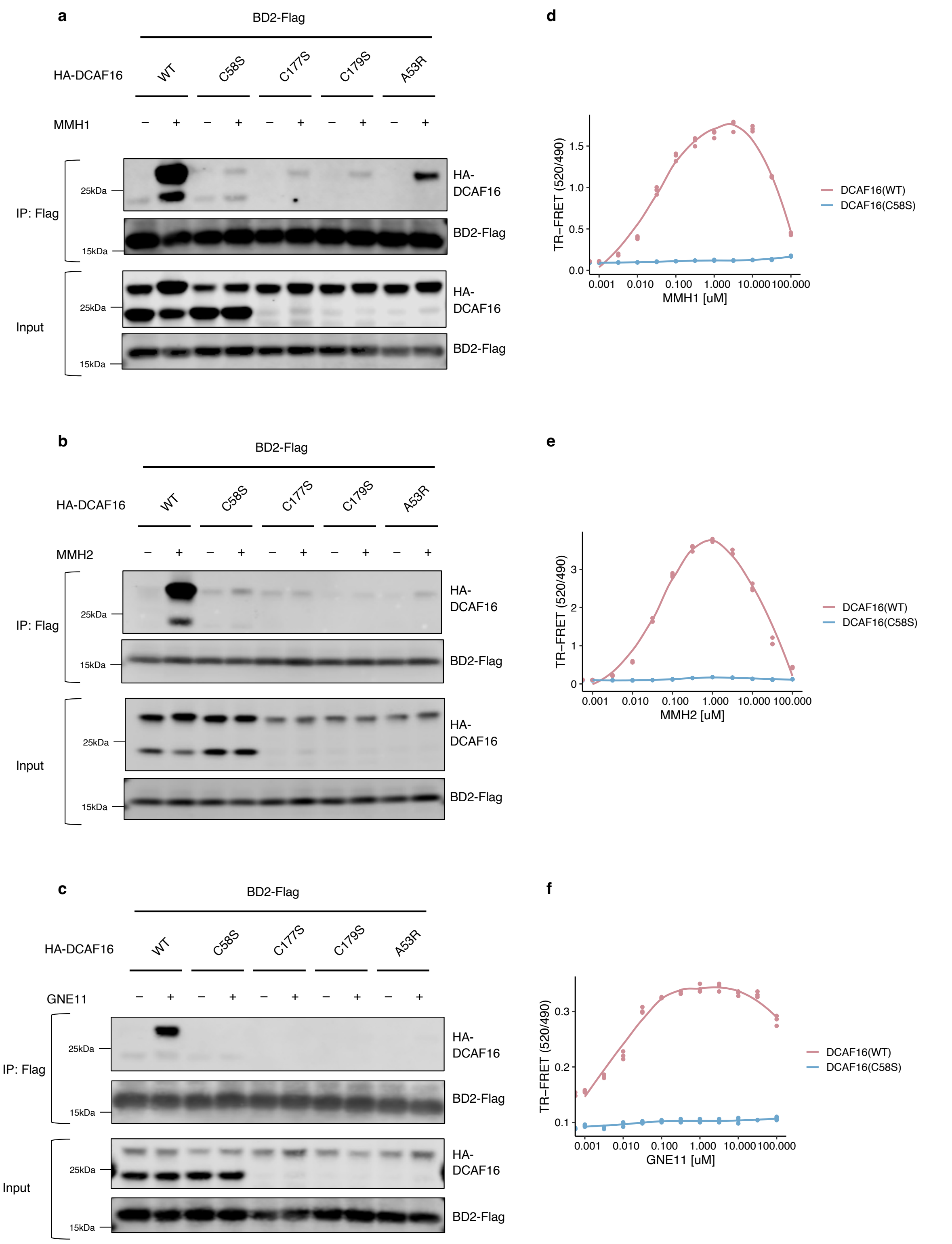

Supplementary Fig. 6: Co-immunoprecipitation and TR-FRET validation of representative DCAF16 mutants. a. Flag immunoprecipitation (IP) followed by Western blots in the presence of DMSO or MMH1 at 0.1 µM from 293T cells transfected with indicated HA-DCAF16 mutants and $BRD4_{BD2}$-Flag constructs. **b.** Flag immunoprecipitation (IP) followed by Western blots in the presence of DMSO or MMH2 at 0.1 µM from 293T cells transfected with indicated HA-DCAF16 mutants and $BRD4_{BD2}$-Flag constructs. **c.** Flag immunoprecipitation (IP) followed by Western blots in the presence of DMSO or GNE11 at

1 μM from 293T cells transfected with indicated HA-DCAF16 mutants and $BRD4_{BD2}$-Flag constructs. **d.** TR-FRET signal for DDB1-DCAF16(WT)- or DDB1-DCAF16(C58S)-BODIPY to $BRD4_{BD2}$-terbium with increasing concentrations of MMH1 (n=3). **e.** TR-FRET signal for DDB1-DCAF16(WT)- or DDB1-DCAF16(C58S)-BODIPY to $BRD4_{BD2}$-terbium with increasing concentrations of MMH2 (n=3). **f.** TR-FRET signal for DDB1-DCAF16(WT)- or DDB1-DCAF16(C58S)-BODIPY to $BRD4_{BD2}$-terbium with increasing concentrations of GNE11 (n=3). All western blot data are representative of two independent measurements (For uncropped western blots, see Supplementary Fig. 8).

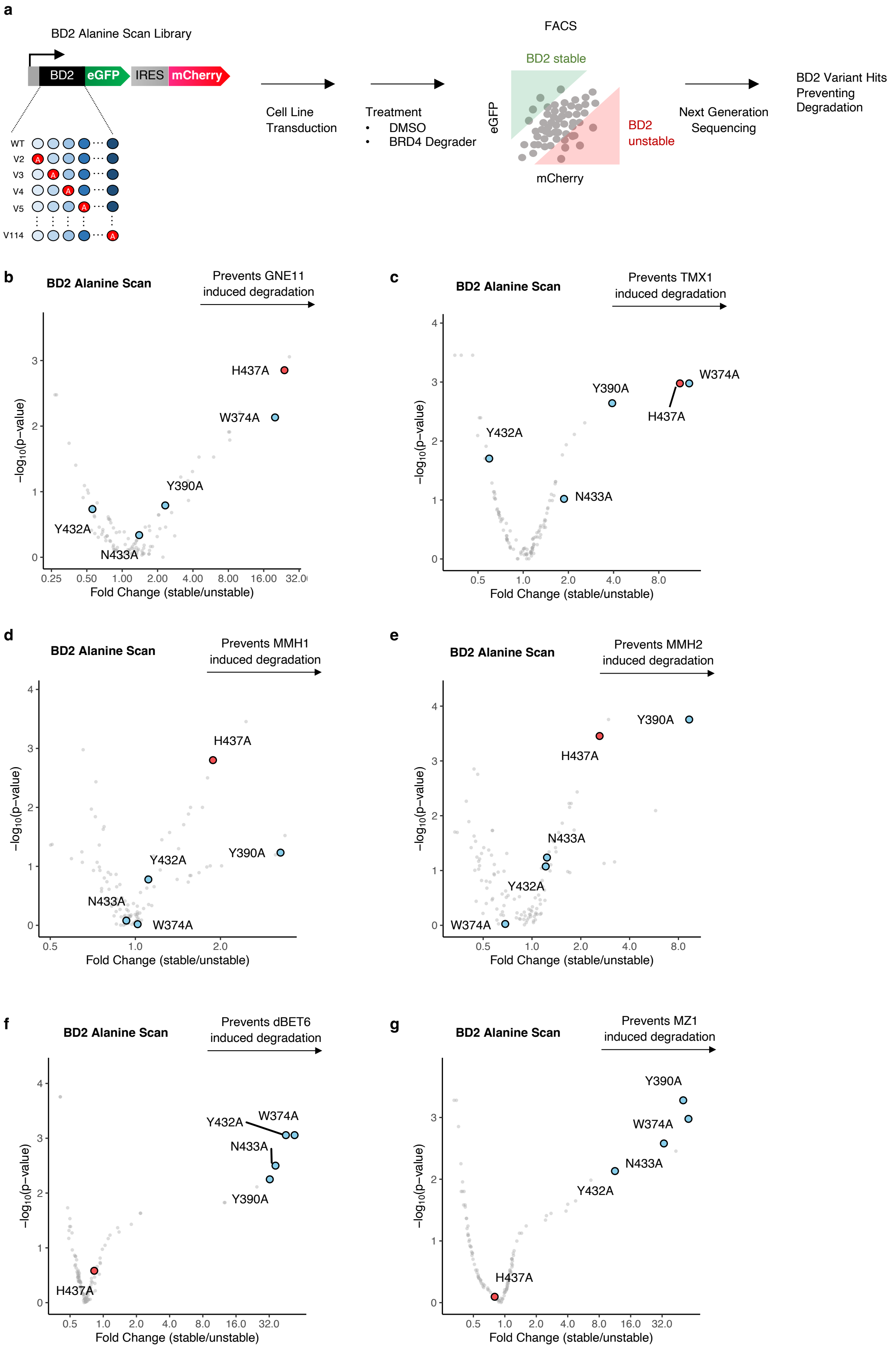

Supplementary Fig. 7: BRD4$_{BD2}$ alanine-scanning reporter screens. a. Schematic of alanine mutagenesis degradation screen of the BRD4 BD2 domain in K562 cells. **b.** BD2 alanine mutagenesis screen for BRD4$_{BD2}$-eGFP stability in K562 cells treated with GNE11 at

1 µM for 16 h (n=2). Statistical analysis was performed using a two-sided empirical rank-sum test. **c.** BD2 alanine mutagenesis screen for $BRD4_{BD2}$-eGFP stability in K562 cells treated with TMX1 at 1 µM for 16 h (n=2). Statistical analysis was performed using a two-sided empirical rank-sum test. **d.** BD2 alanine mutagenesis screen for $BRD4_{BD2}$-eGFP stability in K562 cells treated with MMH1 at 0.1 µM for 16 h (n=3). Statistical analysis was performed using a two-sided empirical rank-sum test. **e.** BD2 alanine mutagenesis screen for $BRD4_{BD2}$-eGFP stability in K562 cells treated with MMH2 at 0.1 µM for 16 h (n=3). Statistical analysis was performed using a two-sided empirical rank-sum test. **f.** BD2 alanine mutagenesis screen for $BRD4_{BD2}$-eGFP stability in K562 cells treated with dBET6 at 1 µM for 16 h (n=2). Statistical analysis was performed using a two-sided empirical rank-sum test. **g.** BD2 alanine mutagenesis screen for $BRD4_{BD2}$-eGFP stability in K562 cells treated with MZ1 at 1 µM for 16 h (n=2). Statistical analysis was performed using a two-sided empirical rank-sum test.

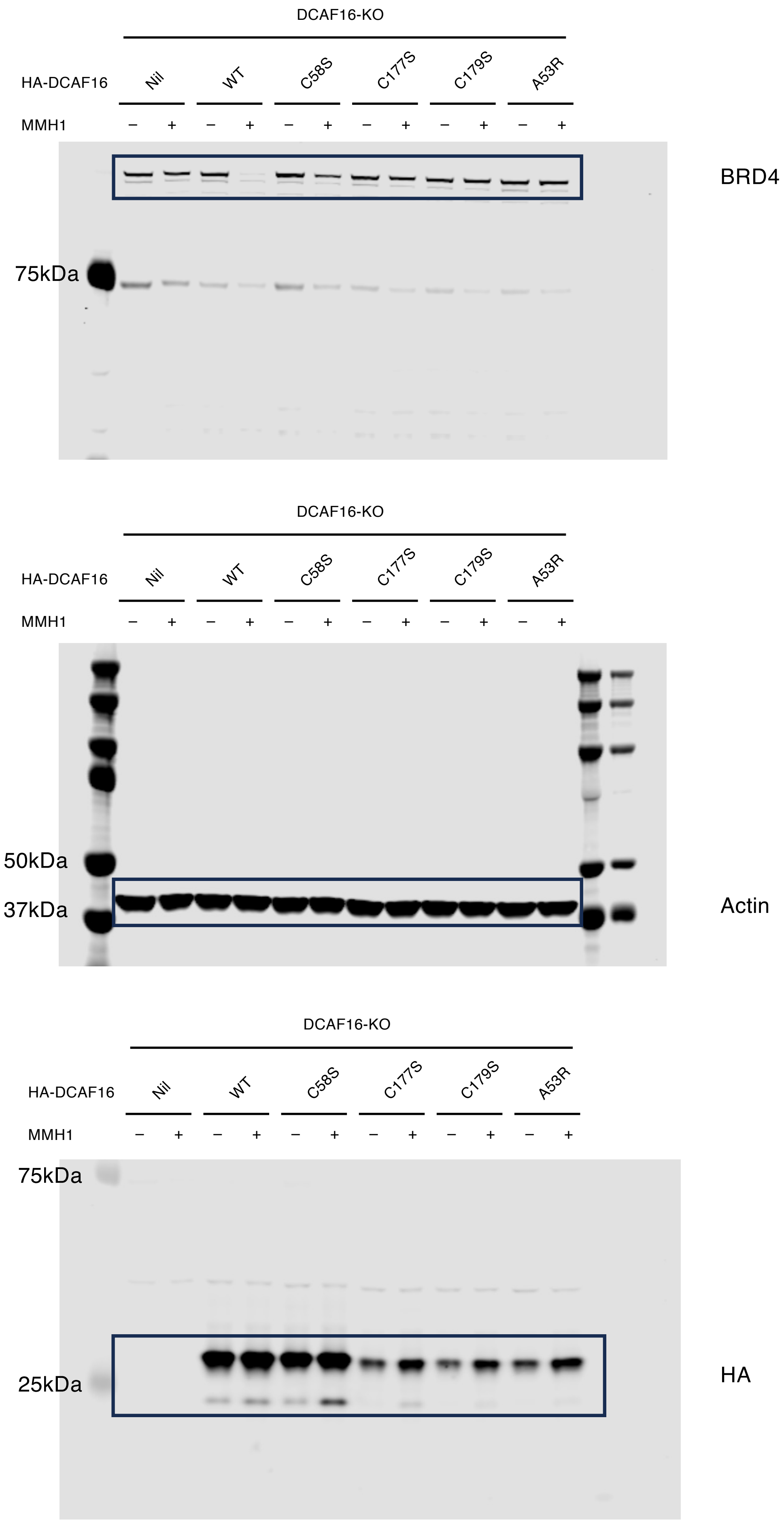

Supplementary Fig. 8: Uncropped western blots for Supplementary Fig. 5a. BRD4 and Actin blots were run on gel 1. HA blot was run on gel 2.

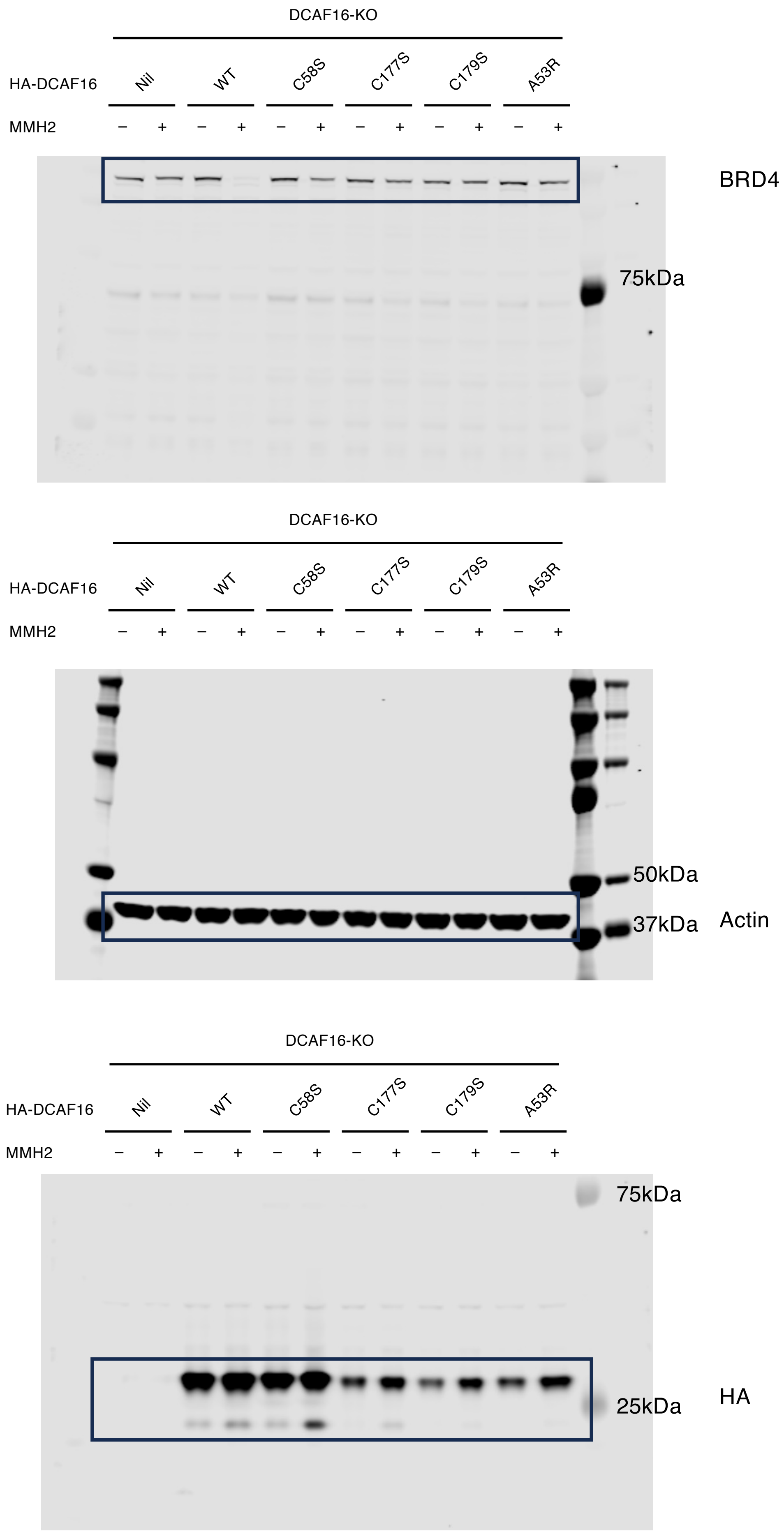

Supplementary Fig. 8 (cont.): Uncropped western blots for Supplementary Fig. 5b. BRD4 and Actin blots were run on gel 1. HA blot was run on gel 2.

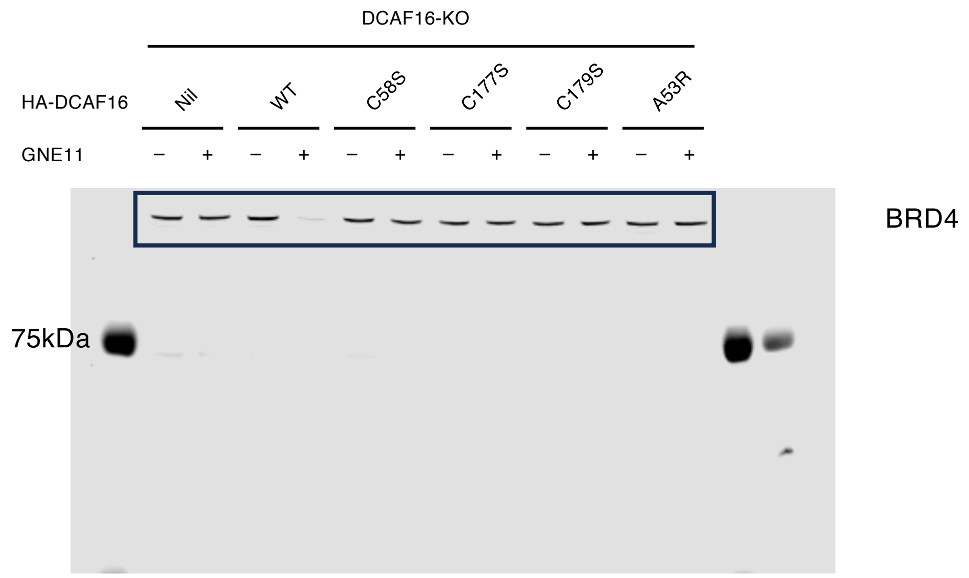

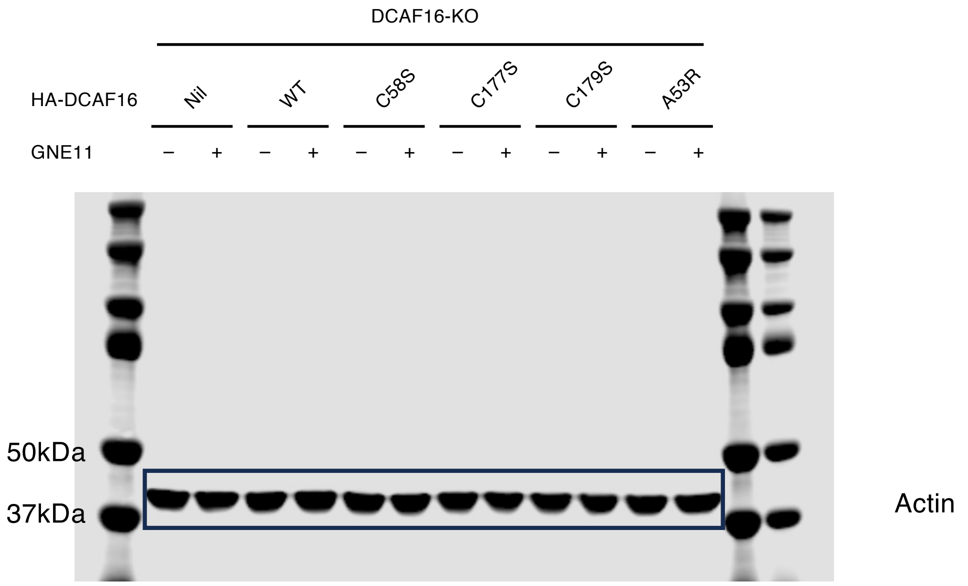

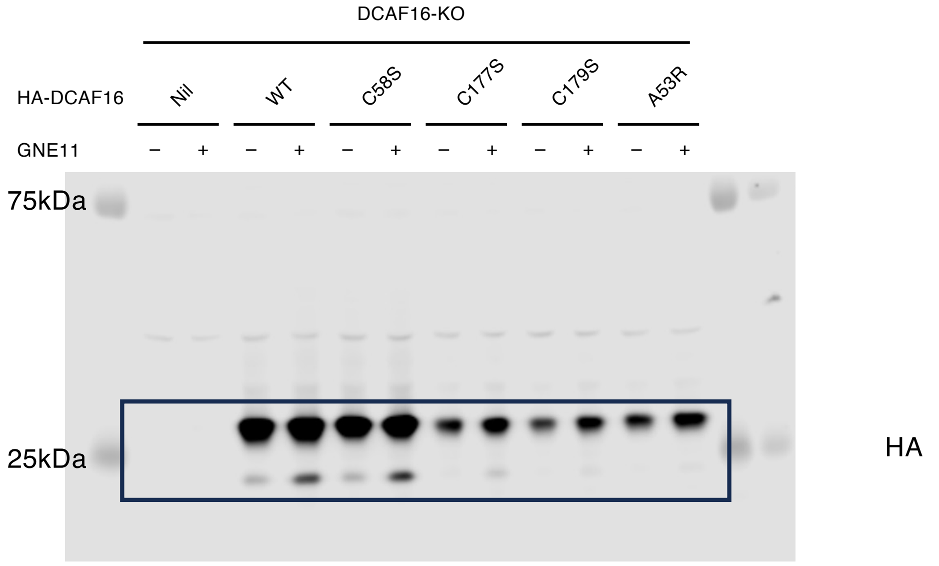

Supplementary Fig. 8 (cont.): Uncropped western blots for Supplementary Fig. 5c. BRD4 and Actin blots were run on gel 1. HA blot was run on gel 2.

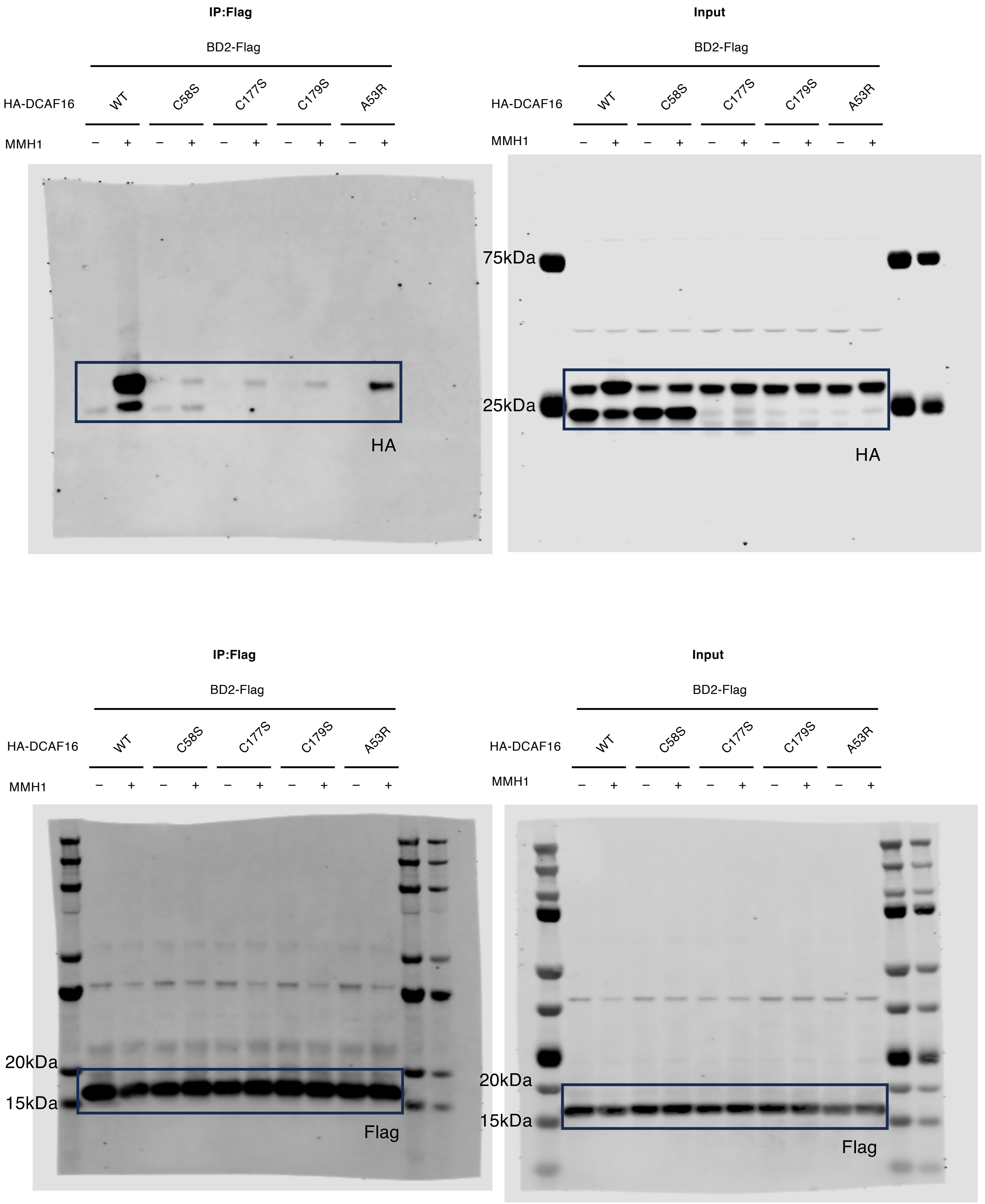

Supplementary Fig. 8 (cont.): Uncropped western blots for Supplementary Fig. 6a. IP blots were run on gel 1. Input blots were run on gel 2.

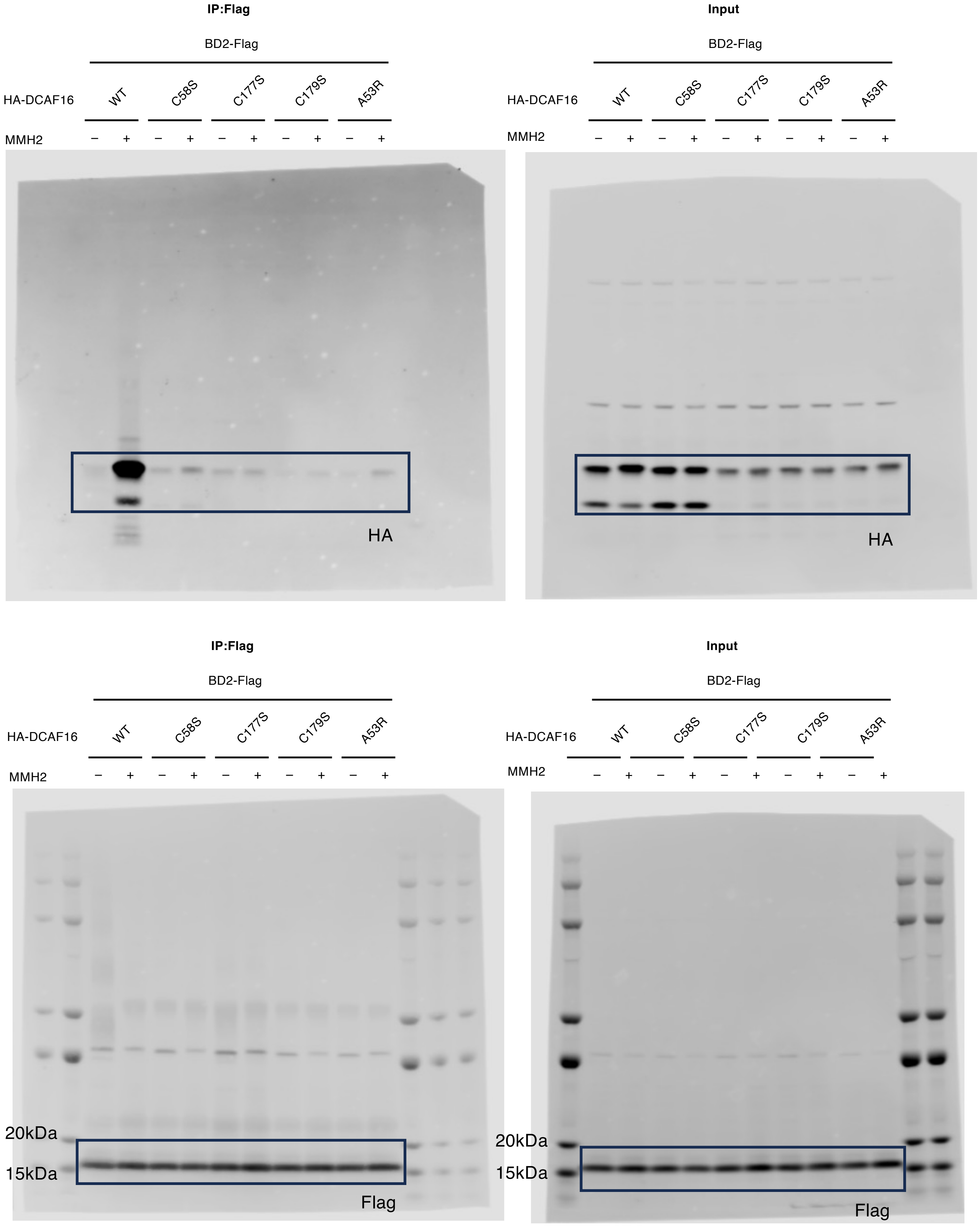

Supplementary Fig. 8 (cont.): Uncropped western blots for Supplementary Fig. 6b. IP blots were run on gel 1. Input blots were run on gel 2.

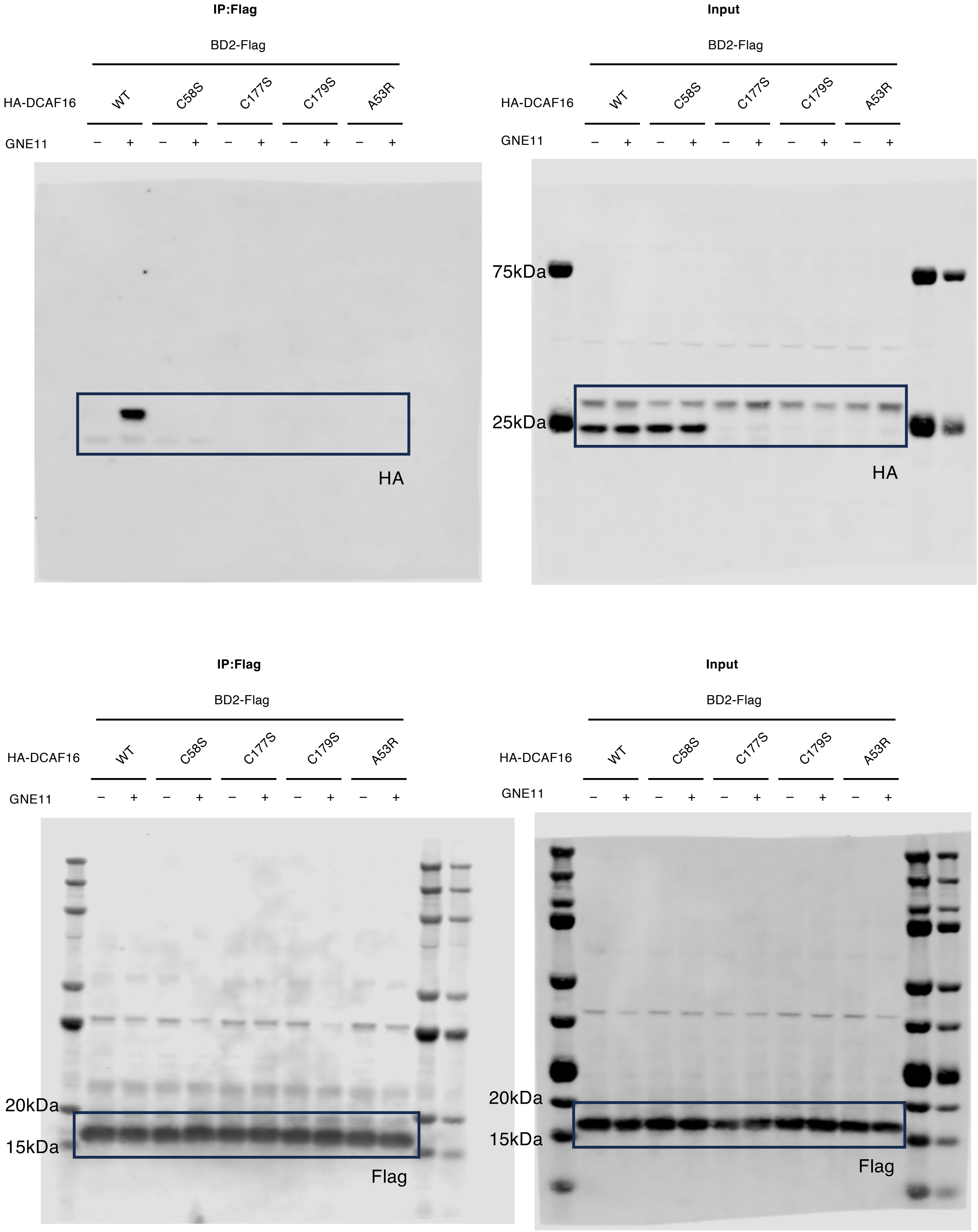

Supplementary Fig. 8 (cont.): Uncropped western blots for Supplementary Fig. 6c. IP blots were run on gel 1. Input blots were run on gel 2.

a Gating strategy for all flow cytometry experiments

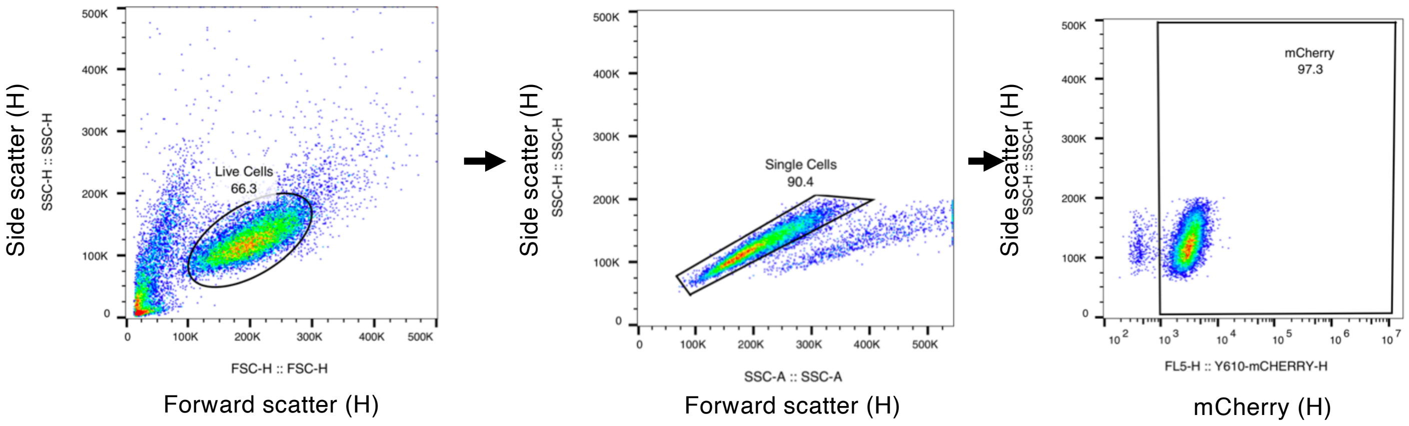

b Gating strategy for FACS sorting screens*

Sorting strategy for reporter stability:
reporter CRISPR screens
$BRD4_{BD2}$ alanine-scanning reporter screens
DCAF16 alanine-scanning reporter screens

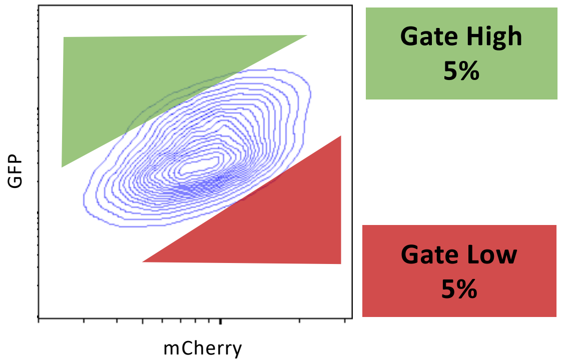

* Cells were first gated for single, mCherry positive cells as in **a**

Supplementary Fig. 9: Gating strategy for flow cytometry

Supplementary Table 1. Cryo-EM data collection, refinement, and validation statistics.

DCAF16-DDB1ΔB-DDA1-MMH2-BRD4$_{BD2}$ (EMDB-29714) (PDB 8G46)

Data collection and processing

Magnification	105,000x
Voltage (kV)	300
Electron exposure (e–/Å^2)	50.27
Defocus range (μm)	-0.8 - -2.0
Pixel size (Å)	0.83
Symmetry imposed	C1
Initial particle images (no.)	14,452,363
Final particle images (no.)	1,433,050
Map resolution (Å)<br>FSC threshold	2.2<br>0.143
Map resolution range (Å)	2-3

Refinement

Initial model used (PDB code)	6Q0R, 6VIX, de novo
Model resolution (Å)<br>FSC threshold	2.3<br>0.5
Map sharpening *B* factor (Å^2)	-74.4
Model composition<br>Non-hydrogen atoms<br>Protein residues<br>Ligands<br>Water	 8,603<br>1,064<br>2<br>110
B factors (Å^2)<br>Protein<br>Ligand<br>Water	 76.88<br>68.85<br>51.89
R.m.s. deviations<br>Bond lengths (Å)<br>Bond angles (°)	 0.007<br>1.068
Validation<br>MolProbity score<br>Clashscore<br>Poor rotamers (%)	 1.70<br>6.37<br>1.70
Ramachandran plot<br>Favored (%)<br>Allowed (%)<br>Disallowed (%)	 97.02<br>2.98<br>0.00

Supplementary Note: Synthesis of compounds, characterization, and spectra

General information

Unless otherwise noted, reagents and solvents were obtained from commercial suppliers and were used without further purification. ^{1}H NMR spectra were recorded on 500 MHz Bruker Avance III spectrometer or on 500 MHz Bruker Avance Neo equipped with carbon detect, liquid nitrogen cooled, Prodigy cryoprobe. Chemical shifts are reported in parts per million (ppm, d) downfield from tetramethylsilane (TMS). Coupling constants (J) are reported in Hz. Spin multiplicities are described as s (singlet), brs (broad singlet), d (doublet), t (triplet), q (quartet), and m (multiplet). Flash column chromatography was carried out using prepacked silica cartridges (from 4 g up to 24 g) from Redisep TM and eluted using an Isco Companion system.

UV and Mass spectrometry chromatograms were obtained from the Acquity UPLC I-Class LCMS system equipped a PDA UV-system and a QDa mass detector. Preparative HPLC was performed using two methods, each using methanol and water each containing 0.045% trifluoroacetic acid (TFA) as the solvent systems:

Method 1: Waters Sunfire C18 column (19 mm X 50 mm, 5 μm) using a gradient of 15-95% methanol in water over 60 min at a flow rate of 43 mL/min.

Method 2: Waters Sunfire C18 column (30 mm X 250 mm, 5 μm) using a gradient of 10 to 100% methanol in water containing 0.045% TFA over 40 min followed by a 5 min flush with 100% methanol, at a flow rate of 40 mL/min.

Purities of all assayed compounds were greater than 95%, as determined by LCMS.

Abbreviations used

B_2pin_2, bis(pinacolato)diboron; Pd_2dba_3, tris(dibenzylideneacetone)dipalladium(0); XPhos, dicyclohexyl[2′,4′,6′-tris(propan-2-yl)[1,1′-biphenyl]-2-yl]phosphane; KOAc, potassium acetate; NaI, sodium iodide; Chloramine-T, sodium chloro(4-methylbenzene-1-sulfonyl)azanide; THF, tetrahydrofuran; $PdCl_2(PPh_3)_2$, bis(triphenylphosphine)palladium(II) dichloride; Et_3N, triethylamine; DMF, dimethylformamide; rt, room temperature; K_2CO_3, potassium carbonate; KCl, potassium chloride; [n-Bu_4N]OAc, tetrabutylammonium acetate; $Pd(OAc)_2$, palladium(II) acetate; Xphos-PdG2, Chloro(2-dicyclohexylphosphino-2′,4′,6′-triisopropyl-1,1′-biphenyl)[2-(2′-amino-1,1′-biphenyl)]palladium(II); DCM, dichloromethane; DIPEA, diisopropylethylamine; prep, preparative.

Synthesis of Intermediates

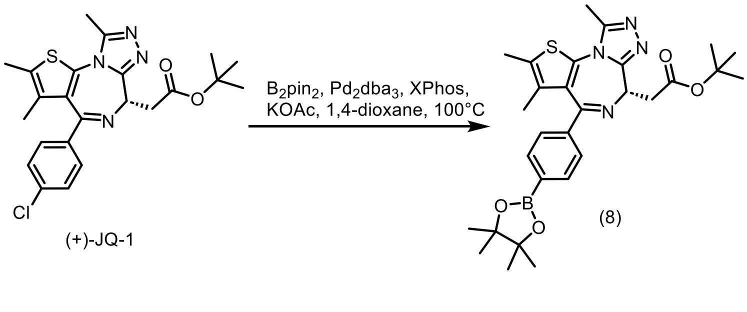

tert-butyl (S)-2-(2,3,9-trimethyl-4-(4-(4,4,5,5-tetramethyl-1,3,2-dioxaborolan-2-yl)phenyl)-6H-thieno[3,2-f][1,2,4]triazolo[4,3-a][1,4]diazepin-6-yl)acetate (8)
To a solution of (+)-JQ-1 (600.0 mg, 1.31 mmol) and B_2pin_2 (332.0 mg, 1.31 mmol) in 1,4-dixoane (5.0 mL) was added XPhos (31.0 mg, 0.065 mmol), Pd_2dba_3 (30.0 mg, 0.033 mmol), and KOAc (386 mg, 3.93 mmol). The reaction mixture was stirred at 100 °C for 17 hours. The reaction mixture was purified via column chromatography (silica gel, eluted with 0% to 15% methanol in dichloromethane) to give **8** (395 mg, 64% yield) as a yellow oil. For **8**, MS (ESI) for $C_{29}H_{38}BN_4O_4S$ $[M+H]^+$: m/z calcd, 549.51; found, 549.49.

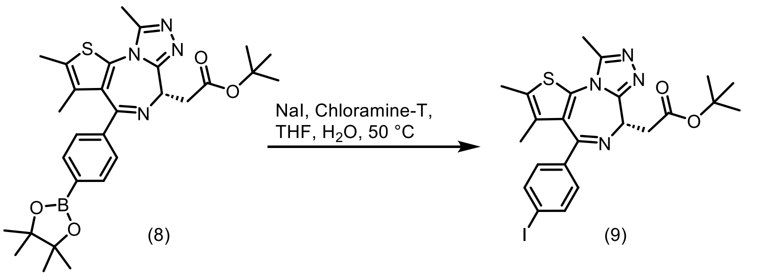

tert-butyl (S)-2-(4-(4-iodophenyl)-2,3,9-trimethyl-6H-thieno[3,2-f][1,2,4]triazolo[4,3-a][1,4]diazepin-6-yl)acetate (9)
To a solution of **8** (200.0 mg, 0.43 mmol) in 1,4-dixoane/H_2O (v/v=2/1, 3.0 mL) was added NaI (129.0 mg, 0.86 mmol) and Chloramine-T 294.0 mg, 1.29 mmol). The reaction mixture was stirred at 50 °C for 4 hours. The reaction mixture was purified directly via prep HPLC (method 1) to give **9** (142 mg, 60% yield) as a yellow solid. ^1H NMR (500 MHz, DMSO-d_6) δ 7.81 (d, J = 8.5 Hz, 2H), 7.20 (d, J = 8.0 Hz, 2H), 4.40 (dd, J = 8.0, 6.5 Hz, 1H), 3.35-3.29 (m, 2H), 2.60 (s, 3H), 2.41 (s, 3H), 1.63 (s, 3H), 1.42 (s, 9H). MS (ESI) for $C_{23}H_{26}IN_4O_2S$ $[M+H]^+$: m/z calcd, 549.45; found, 549.38.

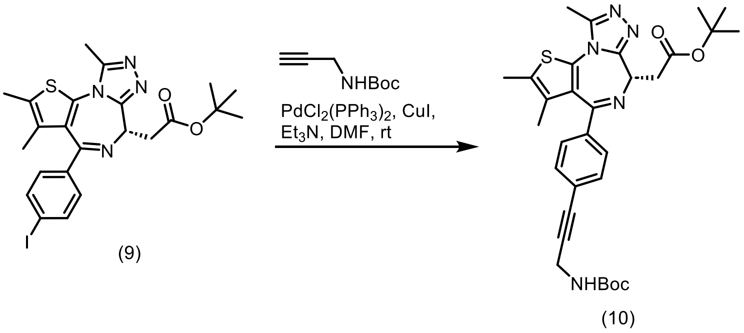

tert-butyl (S)-2-(4-(4-(3-((tert-butoxycarbonyl)amino)prop-1-yn-1-yl)phenyl)-2,3,9-trimethyl-6H-thieno[3,2-f][1,2,4]triazolo[4,3-a][1,4]diazepin-6-yl)acetate (10)
To a solution of **9** (40.0 mg, 0.073 mmol) and N-Boc-propargylamine (23.0 mg, 0.15 mmol) in Et_3N/DMF (v/v=1/1, 1.2 mL) was added $PdCl_2(PPh_3)_2$ (5.1 mg, 7.3 μmol), and CuI (2.8 mg, 14.6 μmol). The reaction mixture was stirred at rt for 3 hours. The reaction mixture was purified directly via prep HPLC (method 1) to give **10** (33.1 mg, 79% yield) as a yellow oil. MS (ESI) for $C_{31}H_{38}N_5O_4S$ $[M+H]^+$: m/z calcd, 576.26; found, 576.18.

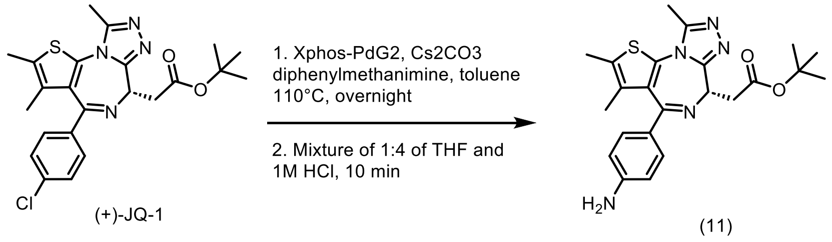

tert-butyl (S)-2-(4-(4-aminophenyl)-2,3,9-trimethyl-6H-thieno[3,2-f][1,2,4]triazolo[4,3-a][1,4]diazepin-6-yl)acetate (NH_2-JQ1, 11)

The NH_2-JQ1 intermediate was prepared according to the procedure previously described by Dragovich *et al.* with slight modifications[1]. Briefly, a mixture of JQ1 (1.40 g, 3.06 mmol), diphenylmethanimine (1.055 g, 5.82 mmol), Xphos-PdG2 (0.241 g, 0.306 mmol), and cesium carbonate (2.994 g, 9.19 mmol) in toluene (20 mL) was heated at 110 °C and left to stir overnight. The next day, the mixture was cooled to room temperature, filtered, and the filtrate was concentrated under vacuum. The residue was purified by flash column chromatography (EtOAc:hexane) and the benzophenone-imine protected product identity was confirmed by LCMS. MS (ESI) for $C_{36}H_{36}N_5O_2S$ $[M+H]^+$: m/z calcd, 602.77; found 602.40. The imine was hydrolyzed by the addition of 20 mL THF, and adding 5 mL of 1 M HCl, followed by stirring for approximately 10 minutes at room temperature. The mixture was dried under vacuum to yield **11** (927 mg, 2.12 mmol, overall yield 69.2 %). MS (ESI) for $C_{23}H_{28}N_5O_2S$ $[M+H]^+$: m/z calcd, 438.56; found 438.35.

Synthesis and Characterization of Final Compounds

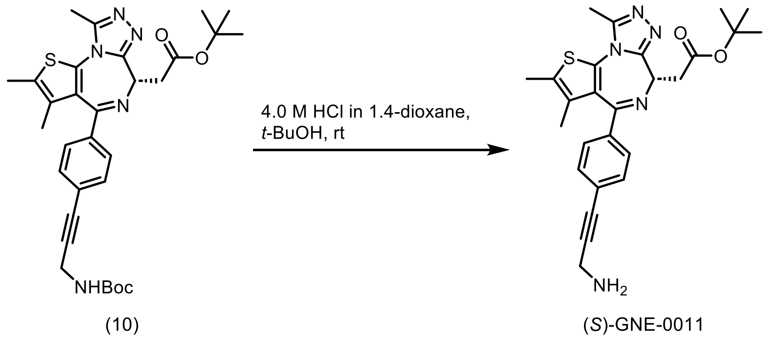

tert-butyl (S)-2-(4-(4-(3-aminoprop-1-yn-1-yl)phenyl)-2,3,9-trimethyl-6H-thieno[3,2-f][1,2,4]triazolo[4,3-a][1,4]diazepin-6-yl)acetate ((S)-GNE-0011, 1)

A solution of **10** (45.0 mg, 0.078 mmol) in *t*-BuOH/4.0 M HCl solution in 1,4-dixoane (v/v=1/1, 1.0 mL) was stirred at rt for 1.5 hours. The reaction mixture was purified directly via prep HPLC to give **(*S*)-GNE-0011 as a TFA salt** (20.0 mg, 41% yield) as a yellow oil. ^1H NMR (500 MHz, DMSO) δ 8.35 (brs, 3H), 7.53 (d, J = 8.5 Hz, 2H), 7.47 (d, J = 8.3 Hz, 2H), 4.45 (dd, J = 8.2, 6.2 Hz, 1H), 4.02 (q, J = 5.5 Hz, 2H), 3.39-3.28 (m, 2H), 2.61 (s, 3H), 2.42 (s, 3H), 1.63 (s, 3H), 1.43 (s, 9H). MS (ESI) for $C_{26}H_{30}N_5O_2S$ $[M+H]^+$: m/z calcd, 476.21; found, 476.31.

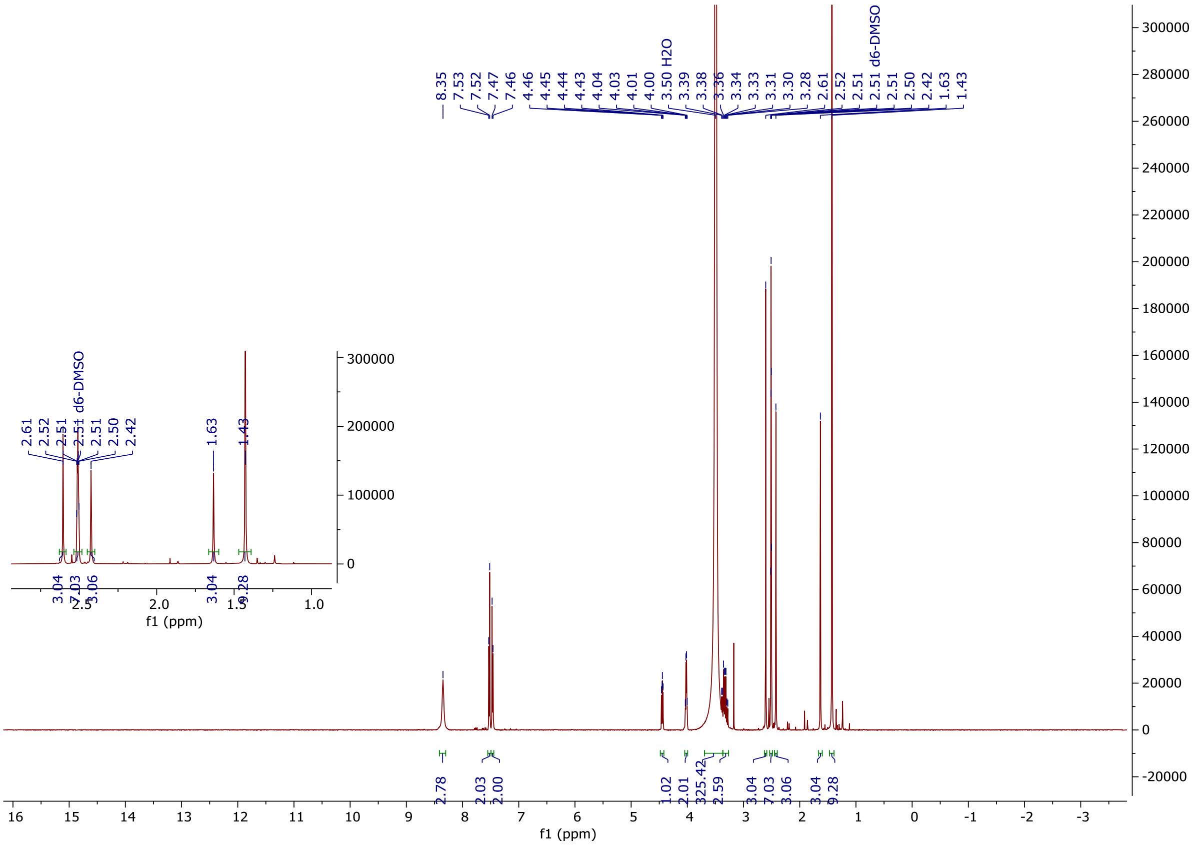

S-GNE-0011 Sm (Mn, 2x3)

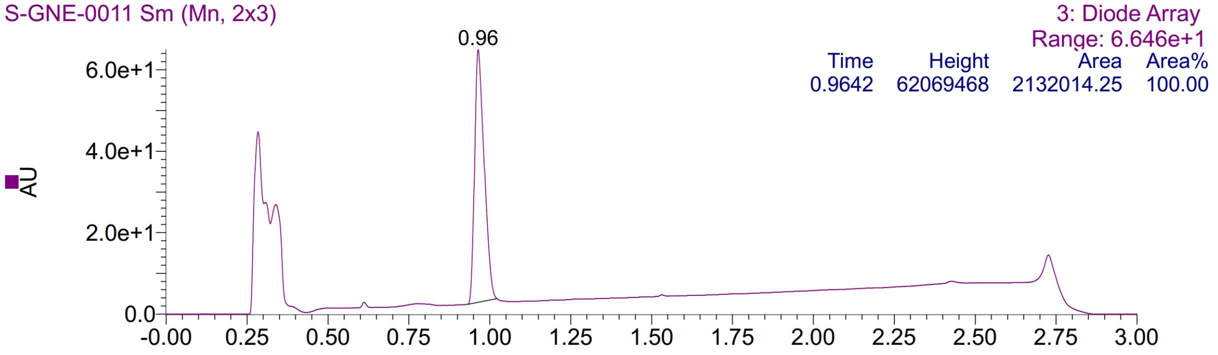

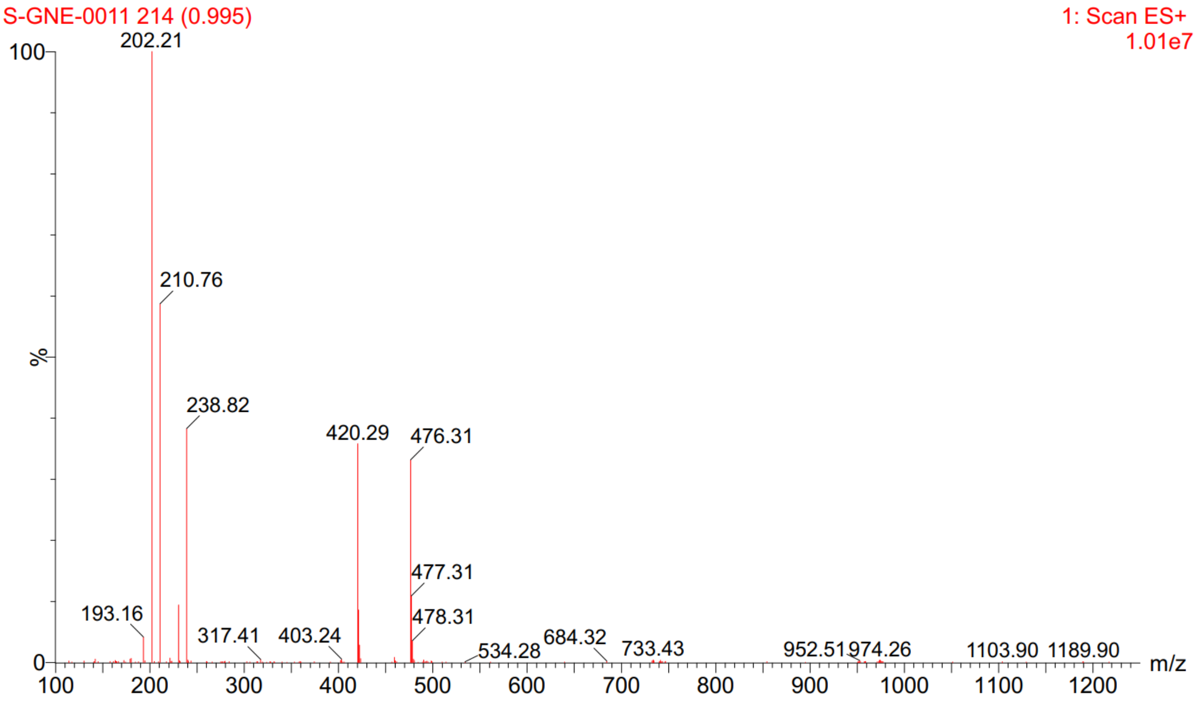

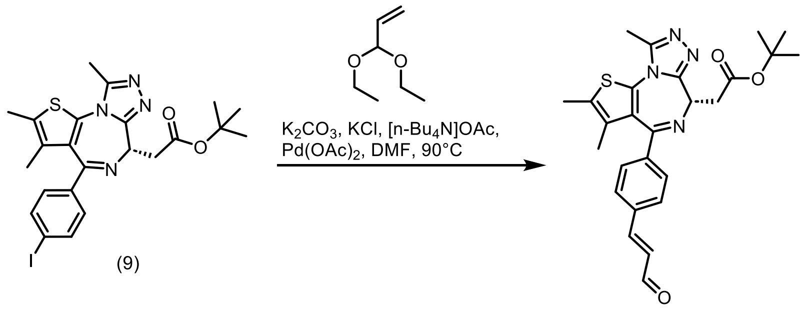

TMX1

tert-butyl (S,E)-2-(2,3,9-trimethyl-4-(4-(3-oxoprop-1-en-1-yl)phenyl)-6H-thieno[3,2-f][1,2,4]triazolo[4,3-a][1,4]diazepin-6-yl)acetate (TMX1, 2)

To a solution of **9** (35.0 mg, 0.064 mmol) and 3,3-diethoxyprop-1-ene (13.0 mg, 0.096 mmol) in DMF (0.6 mL) was added K_2CO_3 (18.0 mg, 0.13 mmol), KCl (5.0 mg, 0.064 mmol), [n-Bu_4N]OAc (39.0 mg, 0.13 mmol), and $Pd(OAc)_2$ (4.0 mg, 0.019 mmol). The reaction mixture was stirred at 90 °C for 8 hours. The reaction mixture was purified directly via prep HPLC (method 1) to give **TMX1** (15.3 mg, 50% yield) as a yellow oil. ^{1}H NMR (500 MHz, DMSO-*d*$_6$) δ 9.69 (d, *J* = 7.5 Hz, 1H), 7.81 (d, *J* = 8.5 Hz, 2H), 7.76 (d, *J* = 16.5 Hz, 1H), 7.49 (d, *J* = 8.0 Hz, 2H), 6.91 (dd, *J* = 16.0, 7.5 Hz, 1H), 4.45 (dd, *J* = 8.0, 6.5 Hz, 1H), 3.40-3.29 (m, 2H), 2.61 (s, 3H), 2.42 (s, 3H), 1.64 (s, 3H), 1.44 (s, 9H). MS (ESI) for $C_{26}H_{29}N_4O_3S$ $[M+H]^+$: m/z calcd, 477.20; found, 477.50.

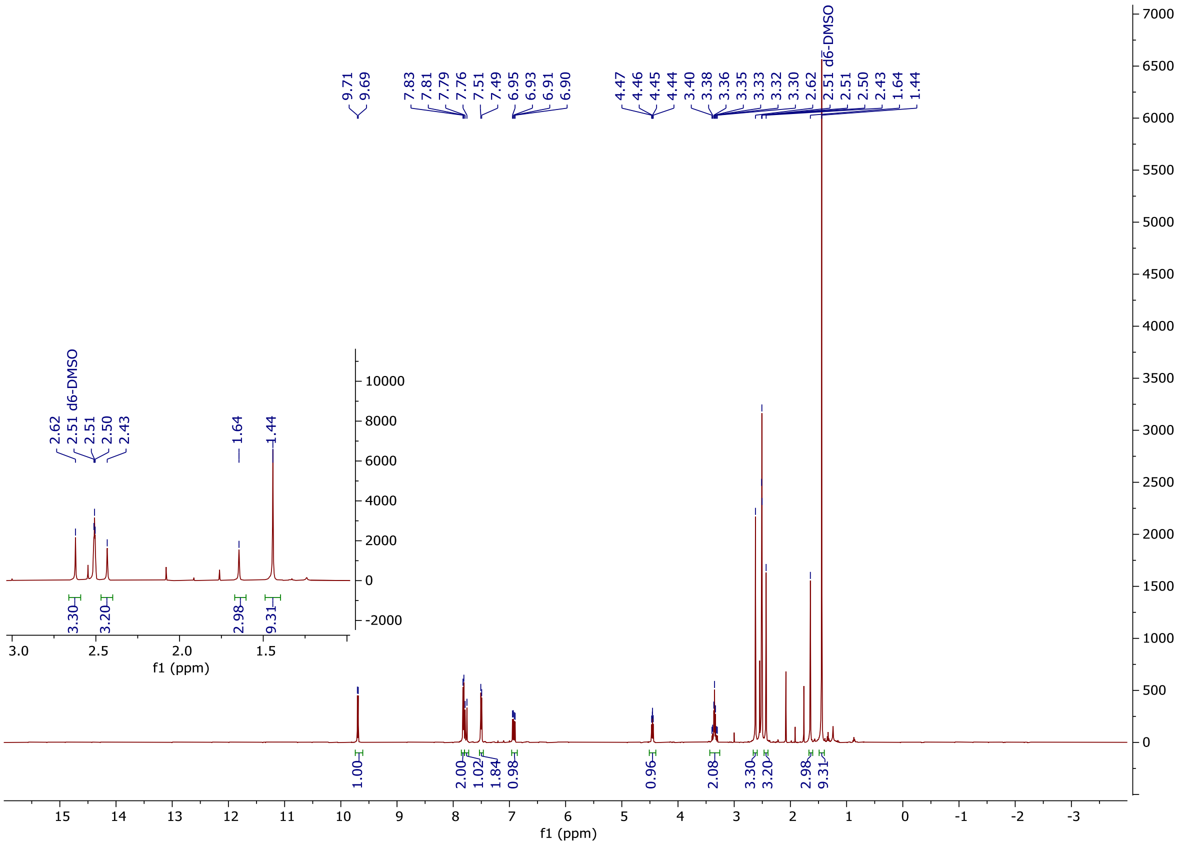

9.71
9.69
7.83
7.81
7.79
7.76
7.51
7.49
6.95
6.93
6.91
6.90
4.47
4.46
4.45
4.44
3.40
3.38
3.36
3.35
3.33
3.32
3.30
2.62
2.51 d6-DMSO
2.51
2.50
2.43
1.64
1.44
1.00
2.00
1.02
1.84
0.98
0.96
2.08
3.30
3.20
2.98
9.31
f1 (ppm)

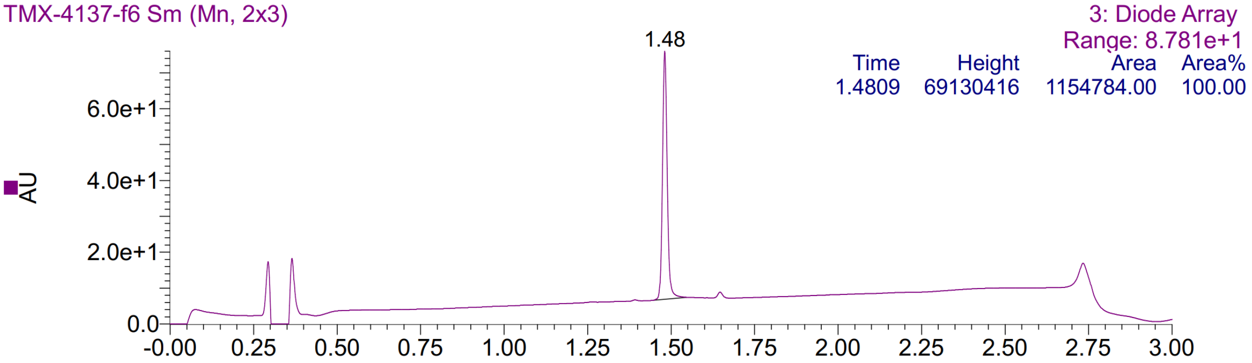

TMX-4137-f6 Sm (Mn, 2x3)
3: Diode Array
Range: 8.781e+1
1.48
Time Height Area Area%
1.4809 69130416 1154784.00 100.00
AU

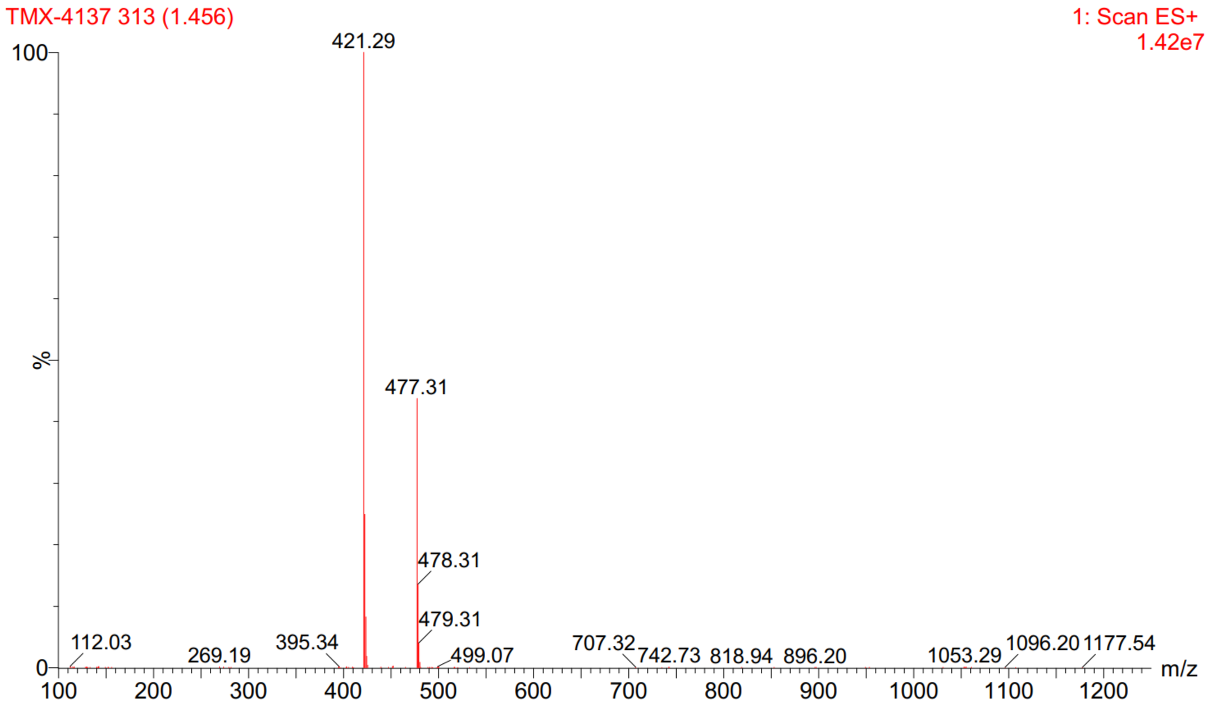

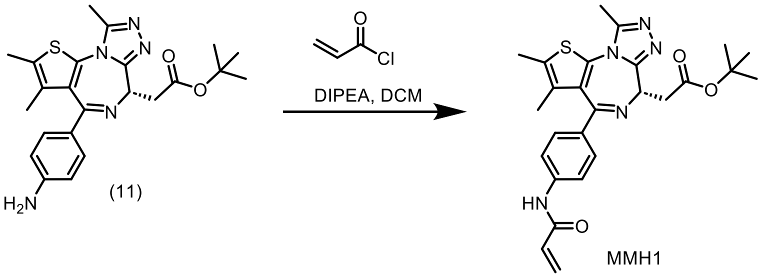

tert-butyl (S)-2-(4-(4-acrylamidophenyl)-2,3,9-trimethyl-6H-thieno[3,2-f][1,2,4]triazolo[4,3-a][1,4]diazepin-6-yl)acetate (MMH1, 3)

MMH1 was prepared by adding acryloyl chloride (4.5 µL, 0.055 mmol) to a mixture of **11** (20.0 mg, 0.046 mmol) and DIPEA (23.4 µL, 0.137 mmol) stirred in 2 mL dichloromethane. The reaction was stirred for 5 minutes and monitored by LCMS, and subsequently quenched with methanol followed by drying under vacuum. The mixture was reconstituted in methanol and purified by prep HPLC (method 2) to give **MMH1** (1.40 mg, 0.00285 mmol, 6.23% yield). ^{1}H NMR (500 MHz, DMSO) δ 10.35 (s, 1H), 7.71 (d, *J* = 9.0 Hz, 2H), 7.40 (d, *J* = 8.4 Hz, 2H), 6.44 (dd, *J* = 16.9, 10.1 Hz, 1H), 6.28 (dd, *J* = 16.9, 2.0 Hz, 1H), 5.78 (dd, *J* = 10.1, 2.0 Hz, 1H), 4.39 (dd, *J* = 8.2, 6.3 Hz, 1H), 3.35 – 3.25 (m, 2H), 2.61 (s, 3H), , 2.43 (s, 3H), 1.67 (s, 3H), 1.43 (s, 9H). MS (ESI) for $C_{26}H_{30}N_5O_3S$ $[M+H]^+$: m/z calcd, 492.61; found 492.35.

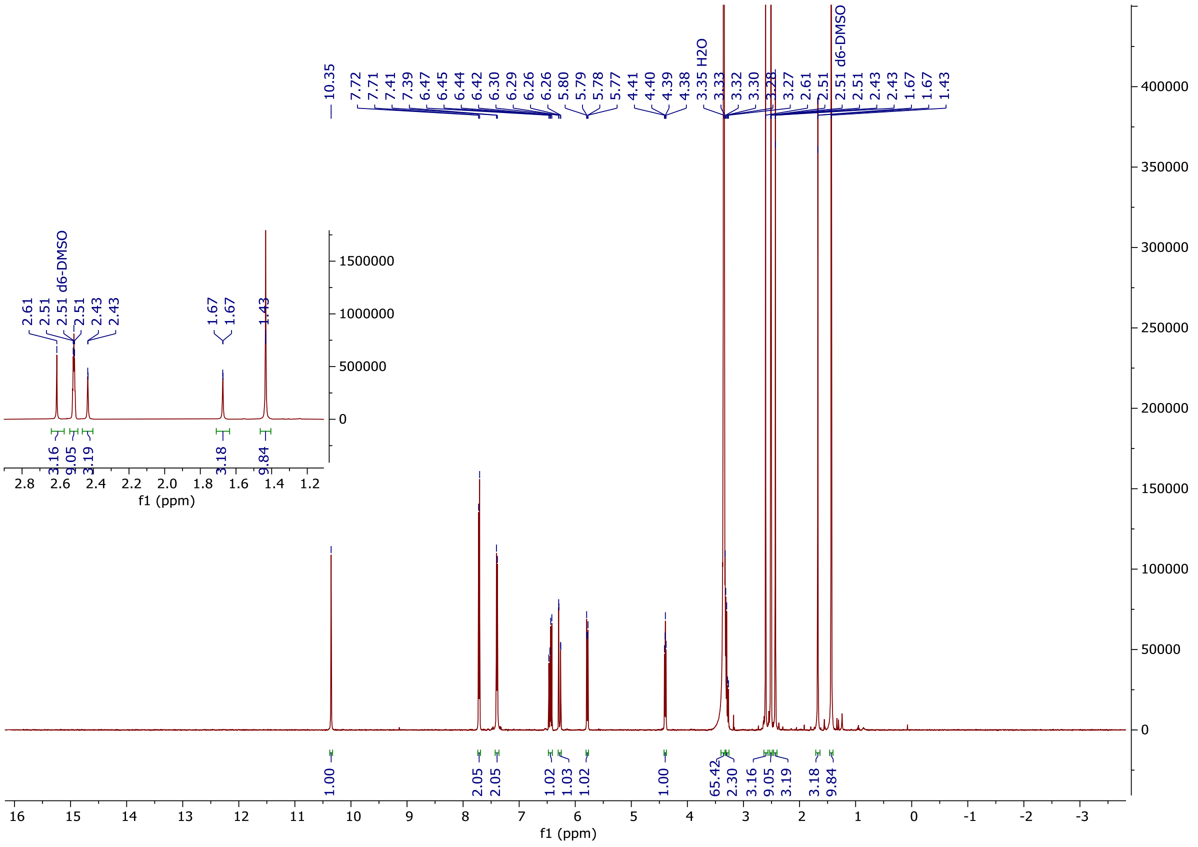
10.35
7.72
7.71
7.41
7.39
6.47
6.45
6.44
6.42
6.30
6.29
6.26
6.26
5.80
5.79
5.78
5.77
4.41
4.40
4.39
4.38
3.35 H2O
3.33
3.32
3.30
3.28
3.27
2.61
2.51
2.51 d6-DMSO
2.51
2.43
2.43
1.67
1.67
1.43
1.00
2.05
2.05
1.02
1.03
1.02
1.00
65.42
2.30
3.16
9.05
3.19
3.18
9.84
f1 (ppm)

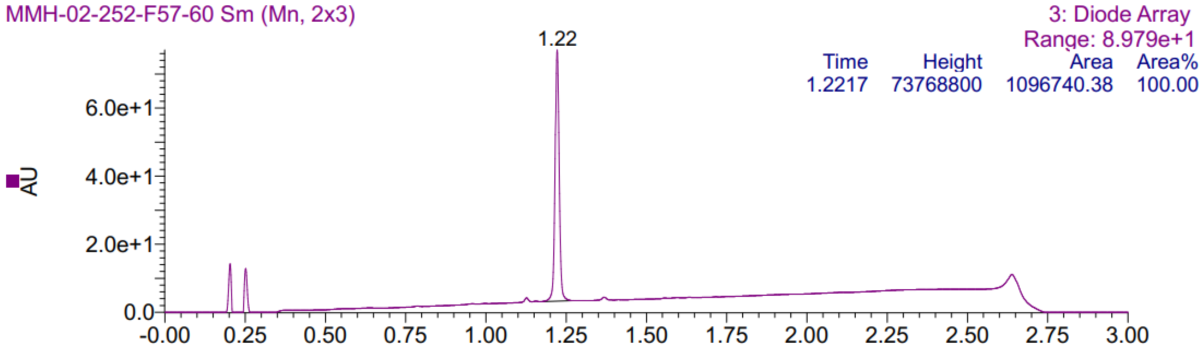
MMH-02-252-F57-60 Sm (Mn, 2x3)
3: Diode Array
Range: 8.979e+1
Time Height Area Area%
1.2217 73768800 1096740.38 100.00
1.22
AU

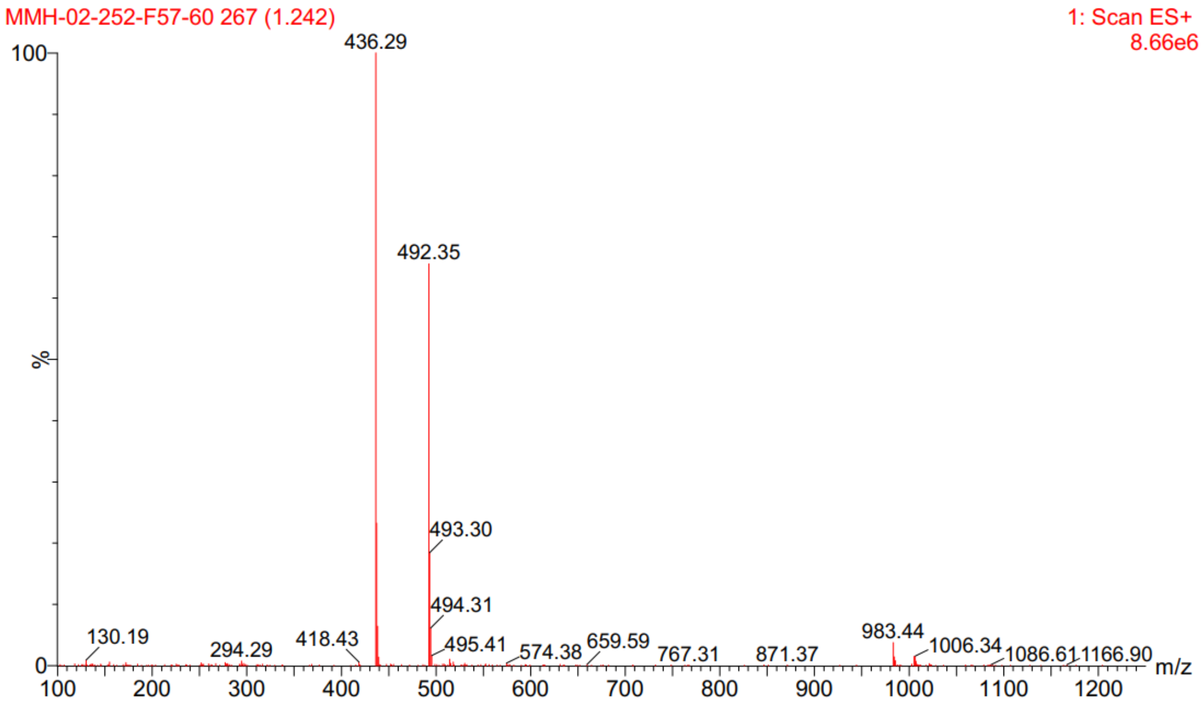

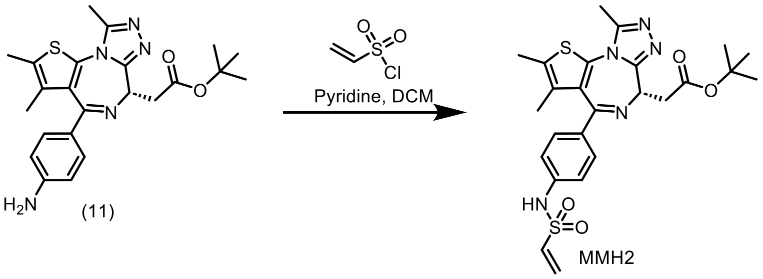

tert-butyl (S)-2-(2,3,9-trimethyl-4-(4-(vinylsulfonamido)phenyl)-6H-thieno[3,2-f][1,2,4]triazolo[4,3-a][1,4]diazepin-6-yl)acetate (MMH2, 4)

Ethenesulfonyl chloride (23.9 µL, 0.263 mmol) was added to a mixture of **11** (9.59 mg, 0.0219 mmol) and pyridine (35.5 µL, 0.438 mmol). The reaction was instantaneous as shown by LCMS. The reaction was washed once with 1 M HCl (1 mL), then the remaining DCM mixture was evaporated. The mixture was reconstituted in methanol to quench excess electrophile and was subsequently purified by prep HPLC (method 2) to give **MMH2** (5.94 mg, 0.0113 mmol, 51.4% yield). ^{1}H NMR (500 MHz, DMSO) δ 10.32 (s, 1H), 7.36 (d, *J* = 8.4 Hz, 2H), 7.17 (d, *J* = 8.85 2H), 6.79 (dd, *J* = 16.4, 10.0 Hz, 1H), 6.14 (d, *J* = 16.4 Hz, 1H), 6.05 (d, *J* = 9.9 Hz, 1H), 4.39 (dd, *J* = 8.3, 6.3 Hz, 1H), 3.38 – 3.24 (m, 2H), 2.60 (s, 3H), 2.42 (s, 3H), 1.65 (s, 1H), 1.43 (s, 9H). MS (ESI) for $C_{25}H_{30}N_5O_4S_2$ $[M+H]^+$: m/z calcd, 528.67; found 528.21.

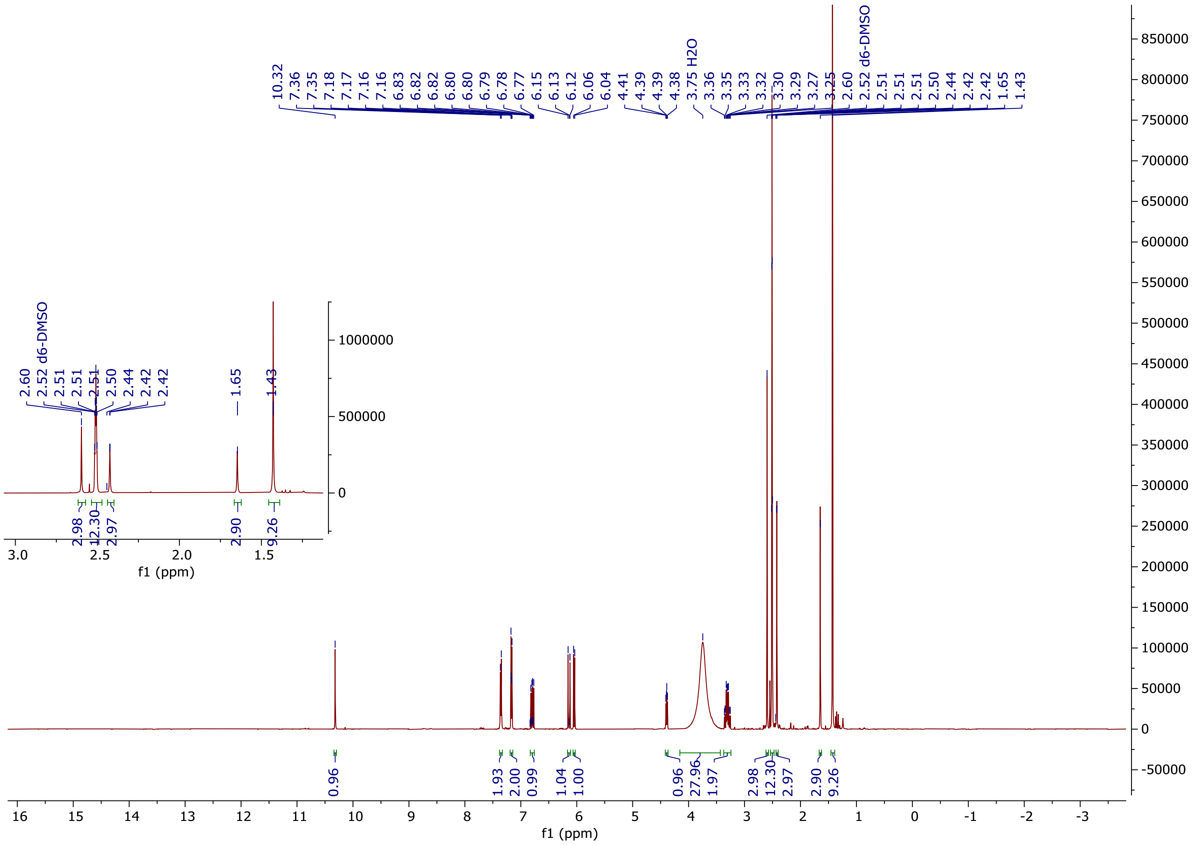
10.32
7.36
7.35
7.18
7.17
7.16
7.16
6.83
6.82
6.82
6.80
6.80
6.79
6.78
6.77
6.15
6.13
6.12
6.06
6.04
4.41
4.39
4.39
4.38
3.75 H2O
3.36
3.35
3.33
3.32
3.30
3.29
3.27
3.25
2.60
2.52 d6-DMSO
2.51
2.51
2.51
2.50
2.44
2.42
2.42
1.65
1.43
0.96
1.93
2.00
0.99
1.04
1.00
0.96
27.96
1.97
2.98
12.30
2.97
2.90
9.26
f1 (ppm)

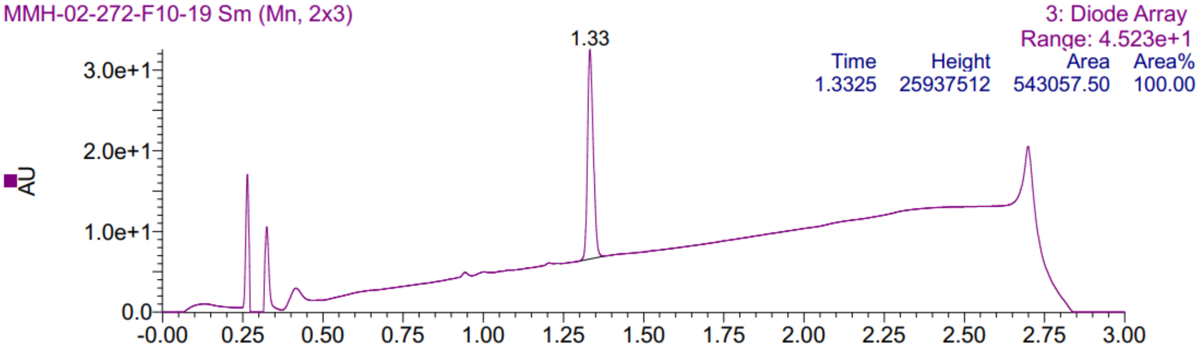
MMH-02-272-F10-19 Sm (Mn, 2x3)
3: Diode Array
Range: 4.523e+1
1.33
Time Height Area Area%
1.3325 25937512 543057.50 100.00
AU

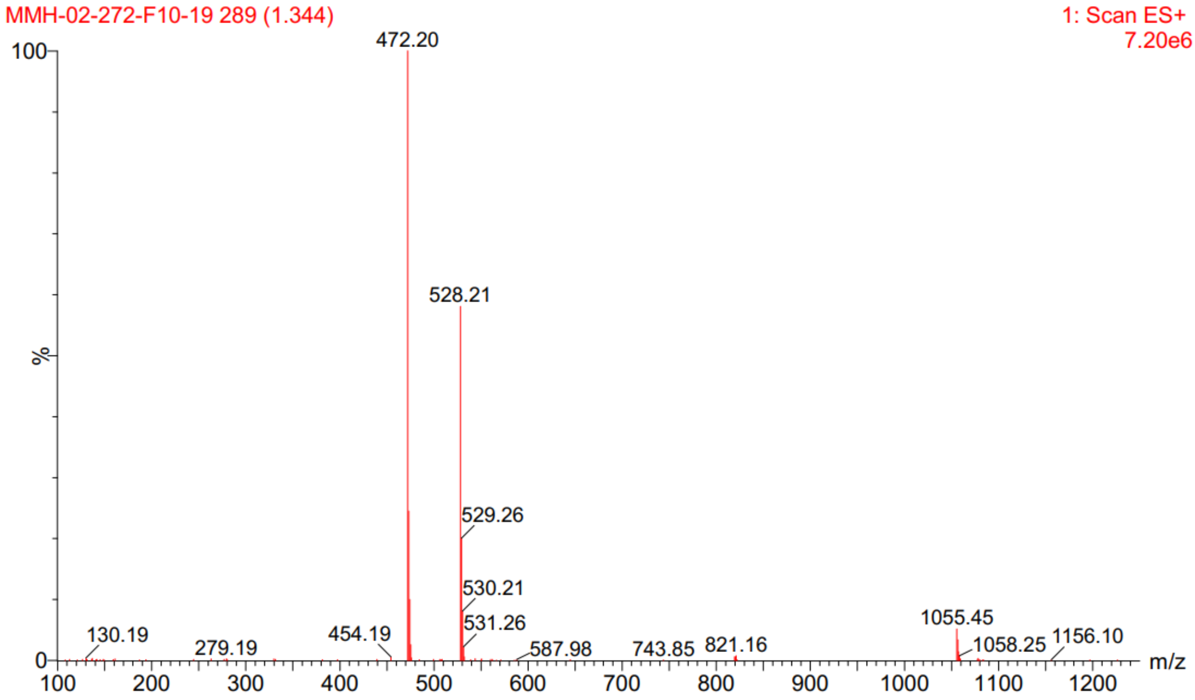

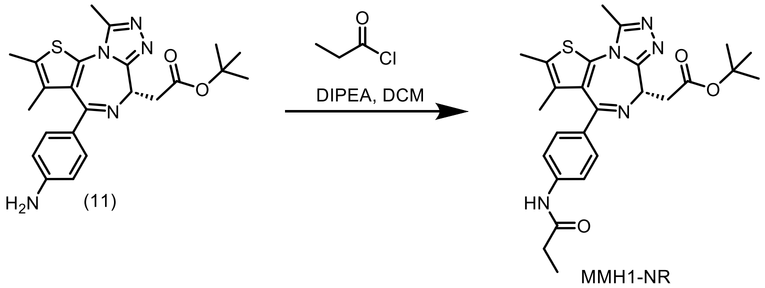

MMH1-NR

tert-butyl (S)-2-(2,3,9-trimethyl-4-(4-propionamidophenyl)-6H-thieno[3,2-f][1,2,4]triazolo[4,3-a][1,4]diazepin-6-yl)acetate (MMH1-NR, 5)

Propionyl chloride (2.5 µL, 0.0282 mmol) was added to a mixture containing **11** (10.3 mg, 0.0235 mmol) and DIPEA (8.05 µL, 0.0471 mmol) in 2 mL dichloromethane. The reaction was left to stir for 1 h, before evaporation under vacuum. The mixture was redissolved in methanol and purified by prep HPLC (method 2) to give **MMH1-NR** (7.75mg, 0.0157 mmol, 66.8% yield). ^{1}H NMR (500 MHz, DMSO) δ 10.08 (s, 1H), 7.64 (d, J = 9.1 Hz, 2H), 7.36 (d, J = 8.4 Hz, 2H), 4.39 (dd, J = 8.2, 6.3 Hz, 1H), 3.38 – 3.24 (m, 2H), 2.61 (s, 3H), 2.43 (d, J = 0.9 Hz, 3H), 2.34 (q, J = 7.6 Hz, 2H), 1.66 (d, J = 1.0 Hz, 3H), 1.43 (s, 9H), 1.08 (t, J = 7.6 Hz, 3H). MS (ESI) for $C_{26}H_{32}N_5O_3S$ $[M+H]^+$: m/z calcd, 494.63; found 494.42.

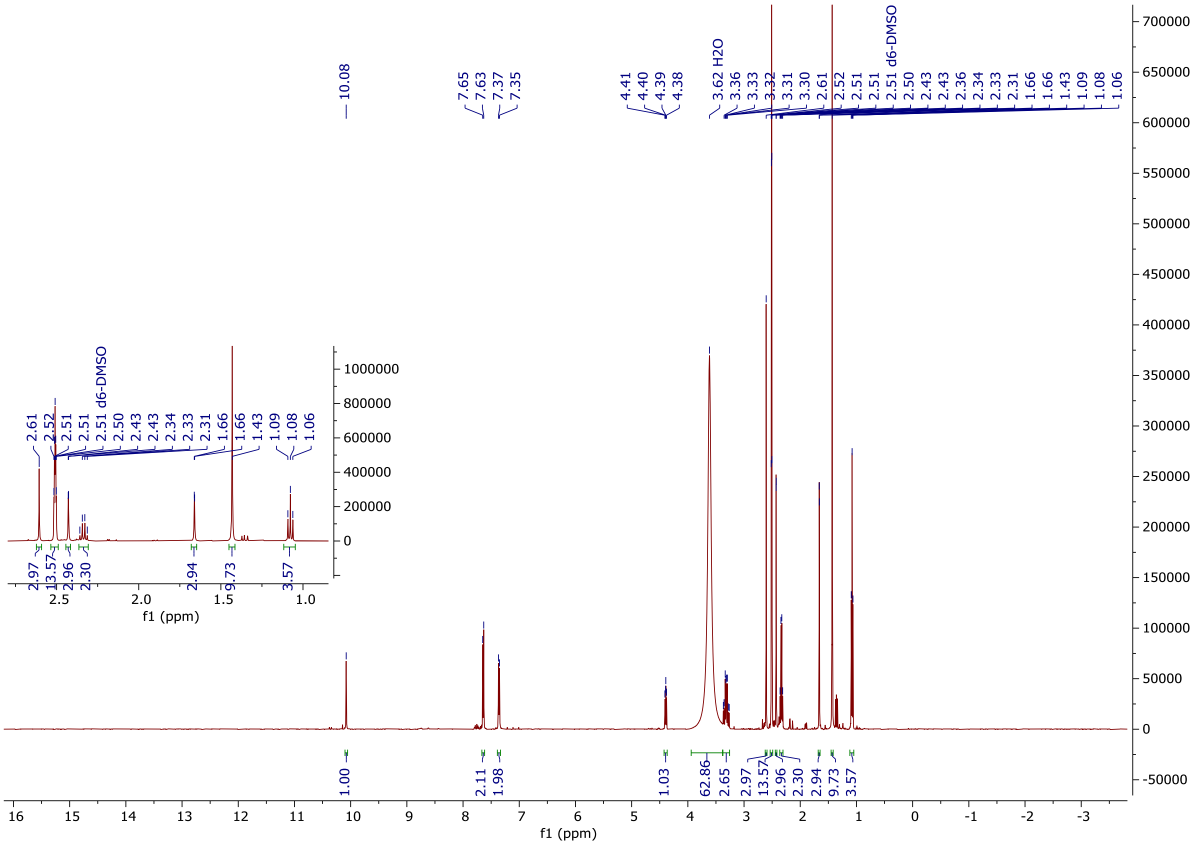

MMH-02-277-f1-8 Sm (Mn, 2x3)

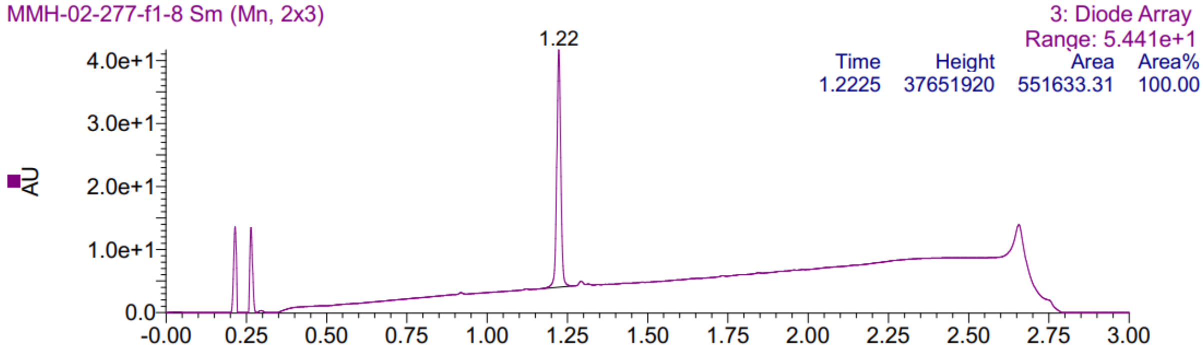

Time	Height	Area	Area%
1.2225	37651920	551633.31	100.00

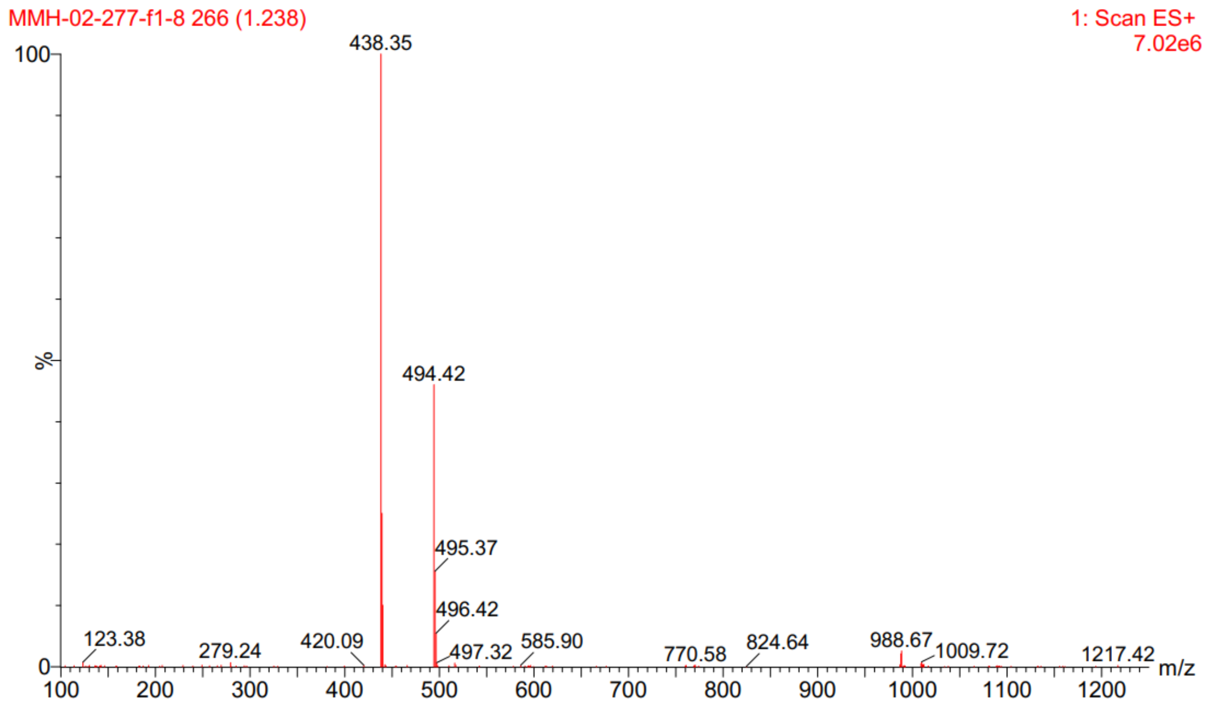

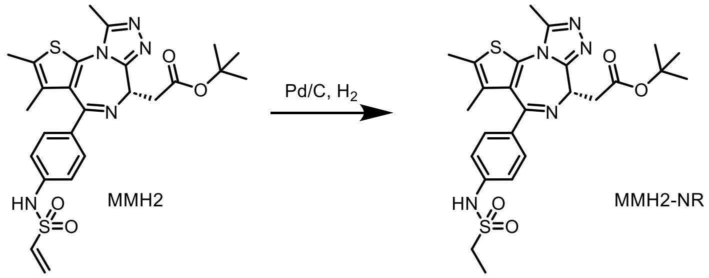

tert-butyl (S)-2-(4-(4-(ethylsulfonamido)phenyl)-2,3,9-trimethyl-6H-thieno[3,2-f][1,2,4]triazolo[4,3-a][1,4]diazepin-6-yl)acetate (MMH2-NR, 6)

To a solution of 4 mL of ethanol, was added **MMH2** (2.26 mg, 0.00428 mmol) and 10% palladium on carbon (10.5 mg, 0.00985 mmol), followed by purging with hydrogen gas via a balloon for 10 min. The reaction was subsequently stirred under hydrogen for another 10 min, flushed through celite, then evaporated and lyophilized to quantitatively yield **MMH2-NR** (2.26 mg, 0.00427 mmol, 99.7% yield). ^{1}H NMR (500 MHz, DMSO) δ 10.09 (s, 1H), 7.37 (d, J = 8.5 Hz, 2H), 7.23 (d, J = 9.0 Hz, 2H), 4.39 (dd, J = 8.3, 6.2 Hz, 1H), 3.33 – 3.24 (m, 2H), 3.12 (q, J = 7.3 Hz, 2H), 2.59 (s, 3H), 2.42 (s, 3H), 1.67 (s, 3H), 1.43 (s, 9H), 1.16 (t, J = 7.3 Hz, 3H). MS (ESI) for $C_{25}H_{32}N_5O_4S_2$ [M+H]$^+$: m/z calcd, 530.68; found 530.33.

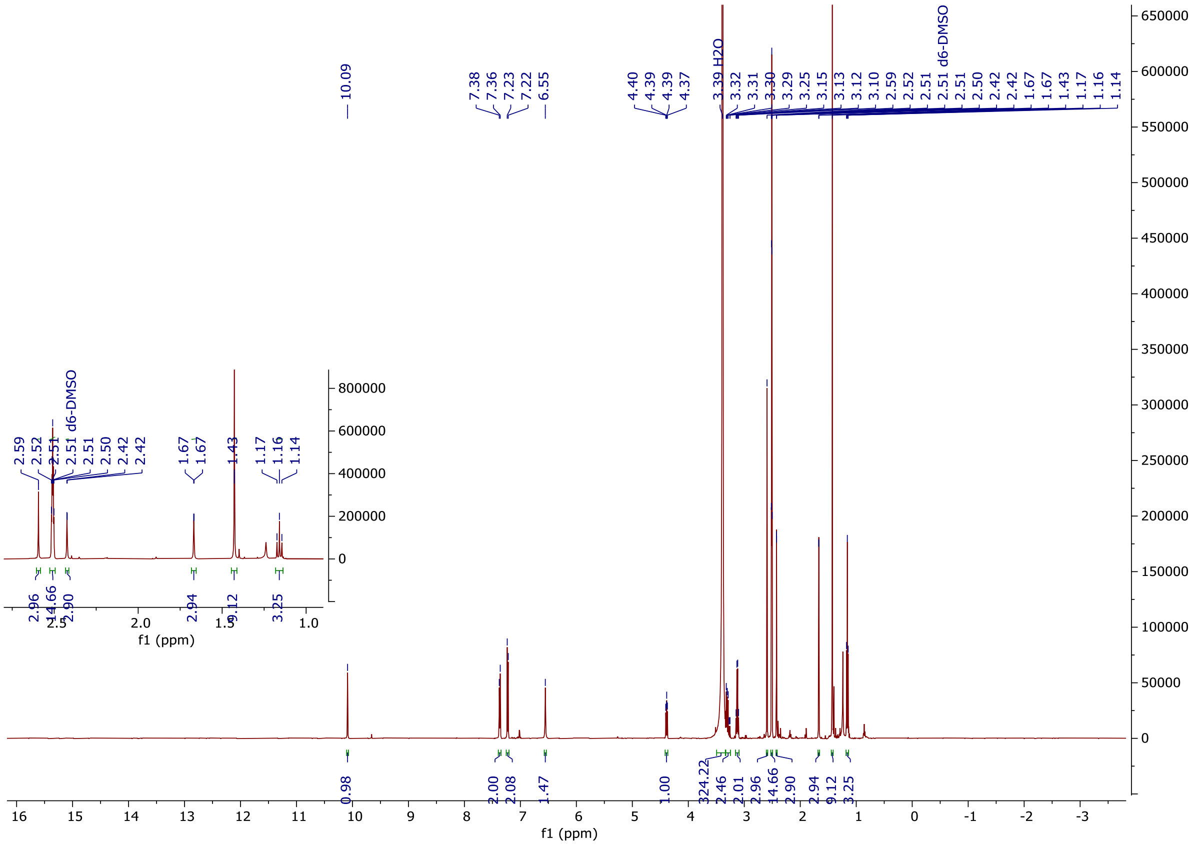
10.09
7.38
7.36
7.23
7.22
6.55
4.40
4.39
4.39
4.37
3.39 H2O
3.32
3.31
3.30
3.29
3.25
3.15
3.13
3.12
3.10
2.59
2.52
2.51
2.51 d6-DMSO
2.51
2.50
2.42
2.42
1.67
1.67
1.43
1.17
1.16
1.14
0.98
2.00
2.08
1.47
1.00
324.22
2.46
2.01
2.96
14.66
2.90
2.94
9.12
3.25
f1 (ppm)

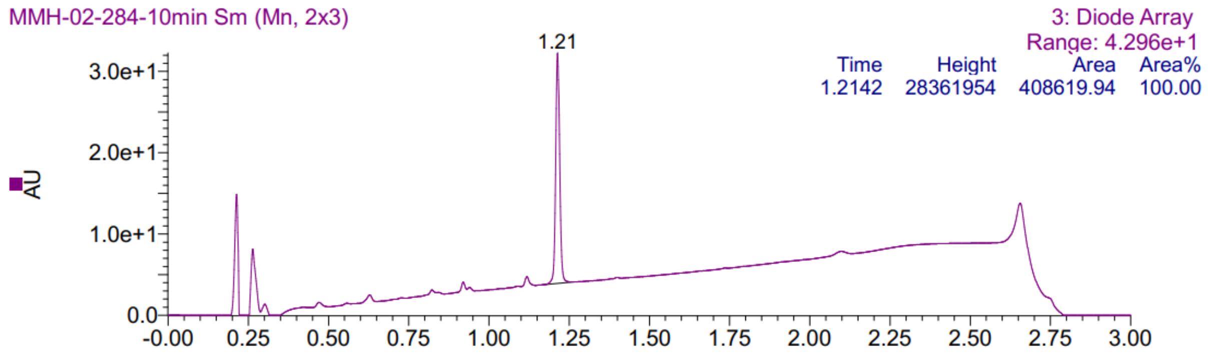
MMH-02-284-10min Sm (Mn, 2x3)
3: Diode Array
Range: 4.296e+1
1.21
Time Height Area Area%
1.2142 28361954 408619.94 100.00
AU

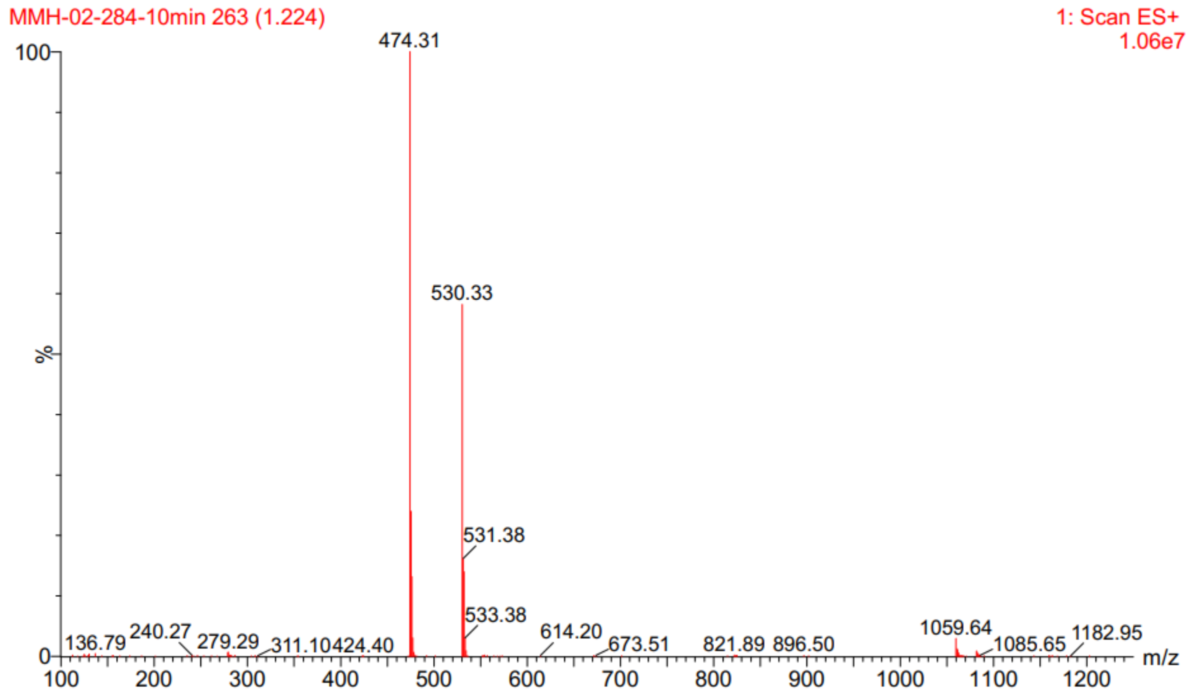

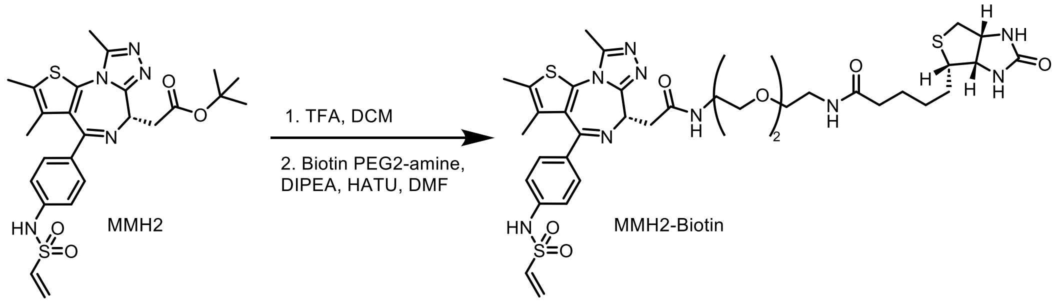

5-((3aS,4S,6aR)-2-oxohexahydro-1H-thieno[3,4-d]imidazol-4-yl)-N-(2-(2-(2-(2-((S)-2,3,9-trimethyl-4-(4-(vinylsulfonamido)phenyl)-6H-thieno[3,2-f][1,2,4]triazolo[4,3-a][1,4]diazepin-6-yl)acetamido)ethoxy)ethoxy)ethyl)pentanamide (MMH2-Biotin, 7)

To a solution of **MMH2** (217 mg, 0.411 mmol) in 10 mL dichloromethane was added 1 mL trifluoroacetic acid (12.3 mmol). The reaction mixture was left to stir overnight and subsequently dried and purified via prep HPLC (method 2). The acid intermediate was lyophilized to give a yellow powder that was then used for the second step. To a mixture of the acid intermediate (15.2 mg, 0.0323 mmol) in 2 mL DMF, was added Biotin PEG2 amine (15.7 mg, 0.042 mmol), DIPEA (8.35 mg, 0.0646 mmol), and HATU (18.4 mg, 0.0484 mmol). The mixture was directly injected for purification via prep HPLC (method 2) and subsequently lyophilized to yield **MMH2-Biotin** (14.9 mg, 0.0181 mmol, 56% yield). ^{1}H NMR (500 MHz, DMSO) δ 10.33 (s, 1H), 8.28 (t, J = 5.7 Hz, 1H), 7.84 (t, J = 5.7 Hz, 1H), 7.36 (d, J = 8.4 Hz, 2H), 7.17 (d, J = 8.7 Hz, 2H), 6.41 (br, 1H), 6.79 (dd, J = 16.4, 9.9 Hz, 1H), 6.14 (d, J = 16.4 Hz, 1H), 6.05 (d, J = 9.9 Hz, 1H), 4.51 (t, J = 7.1 Hz, 1H), 4.30 (dd, J = 7.8, 4.9 Hz, 1H), 4.12 (dd, J = 7.7, 4.4 Hz, 1H), 3.53 (m, 4H), 3.46 (t, J = 5.8 Hz, 2H), 3.41 (t, J = 5.9 Hz, 2H), 3.25 (m, 6H), 3.09 (m, 1H), 2.82 (dd, J = 12.2, 5.2 Hz, 1H), 2.60 (s, 3H), 2.59 (m, 1H), 2.55 (m, 1H), 2.42 (s, 3H), 2.07 (t, J = 7.3 Hz, 2H), 1.64 (s, 3H) , 1.60 (m, 1H), 1.48 (m, 3H), 1.29 (m, 2H). MS (ESI) for $C_{37}H_{50}N_9O_7S_3$ $[M+H]^+$: m/z calcd, 828.29; found 828.49.

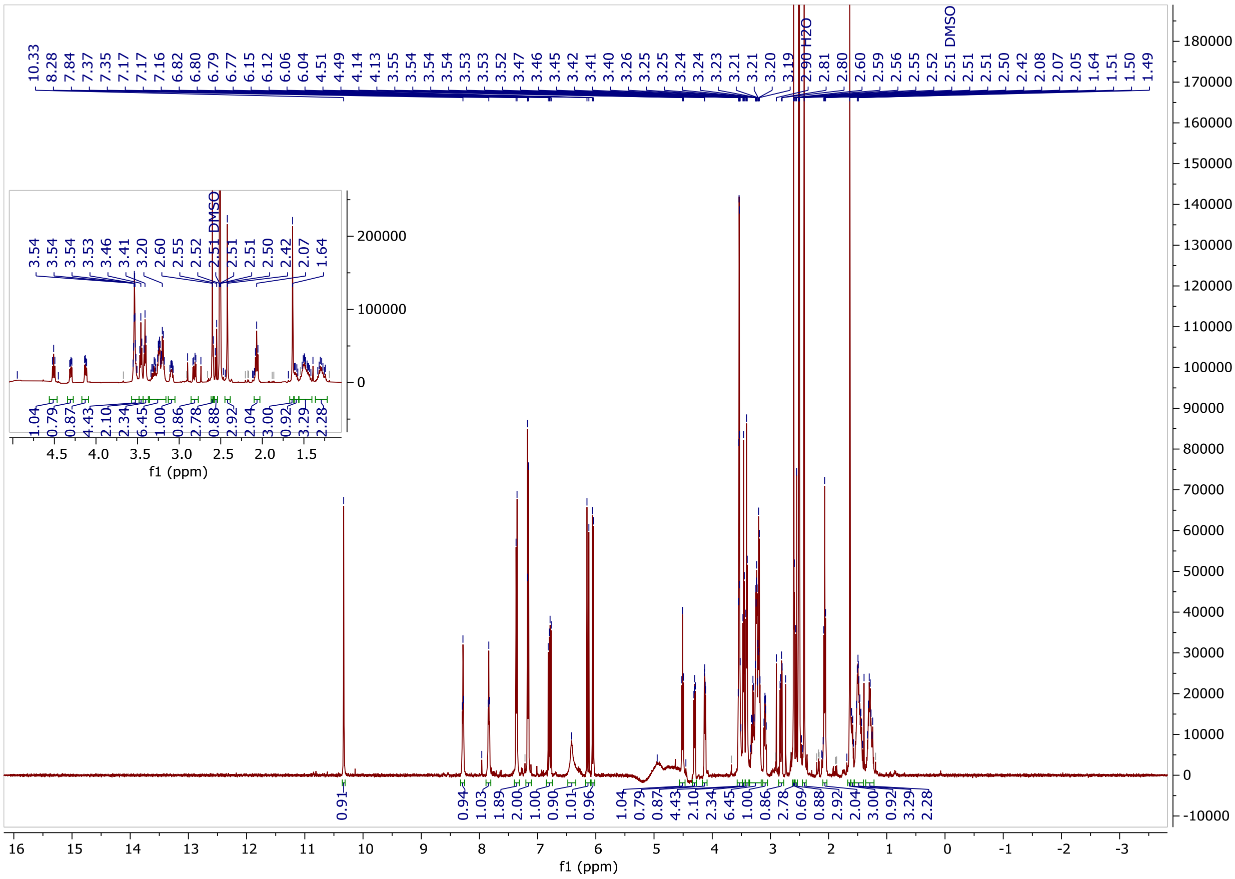
f1 (ppm)
H2O
DMSO

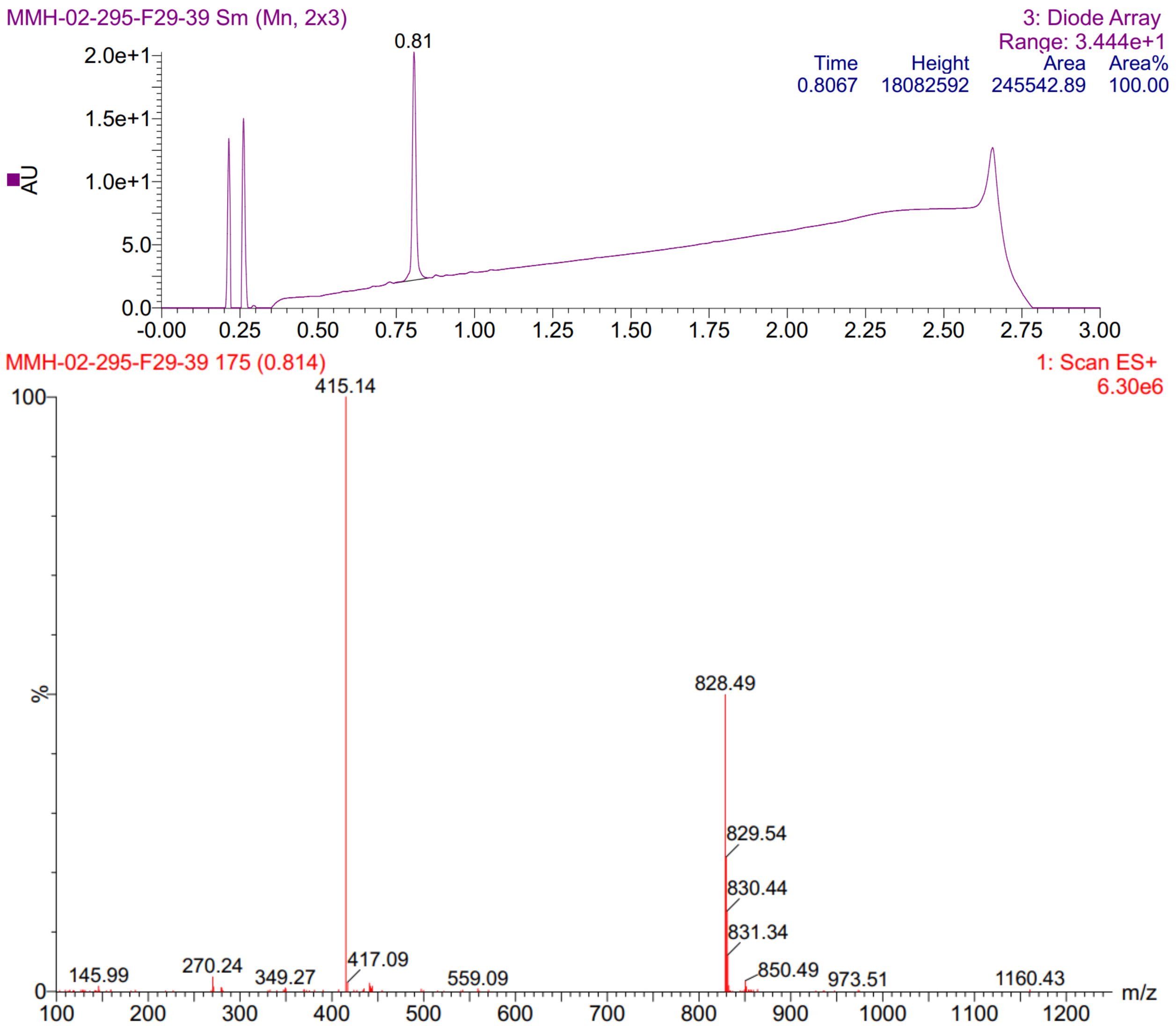

References

1. Dragovich, P. S. *et al.* Antibody-Mediated Delivery of Chimeric BRD4 Degraders. Part 1: Exploration of Antibody Linker, Payload Loading, and Payload Molecular Properties. *J. Med. Chem.* **64**, 2534–2575 (2021).

Supplementary Note: Deep sequencing results for DCAF16 knockout clones

Starting next page

CRISPResso2

Analysis of genome editing outcomes from deep sequencing data

CRISPResso2 run information

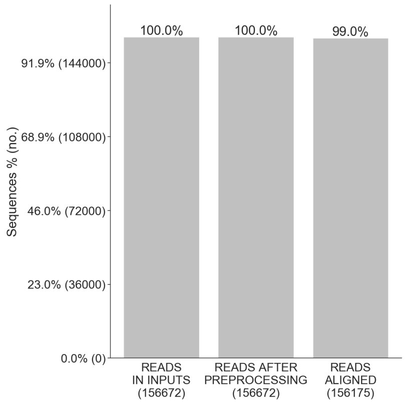

Figure 1a: The number of reads in input fastqs, after preprocessing, and after alignment to amplicons.

Data: [Mapping statistics]

CRISPResso version: 2.0.45

Run completed: 2021-04-03 19:51:04

Amplicon sequence:

```
TGAGGAAGAAGAAAATATTAGTTACCTAAATGAGAGTTCTGGGGAAGAGTGGGATTCCTCTGAAGAAGAGGACTCTATGGTGCCCAACT
TATCGCCTCTTGAGAGTCTTGCCTGGCAGGTTAAGTGCCTTTTAAAATATTCCACAACTTGGAAACCTTTAAATCCTAATTCCTGGTTG
TATCATGCTAAACTGTTGGATC
```

Guide sequence:

```
TGCCAGGCAAGACTCTCAAG
```

Command used:

```
CRISPResso -r1 CRISPResso_Input_Reads_47209e6f-e115-4e4b-bcd6-83b66e584b9b.fastq -a
TGAGGAAGAAGAAAATATTAGTTACCTAAATGAGAGTTCTGGGGAAGAGTGGGATTCCTCTGAAGAAGAGGACTCTATGGTGCCCAACT
TATCGCCTCTTGAGAGTCTTGCCTGGCAGGTTAAGTGCCTTTTAAAATATTCCACAACTTGGAAACCTTTAAATCCTAATTCCTGGTTG
TATCATGCTAAACTGTTGGATC -g TGCCAGGCAAGACTCTCAAG --name fQtMGr --output_folder
CRISPRessoRunfQtMGr --write_cleaned_report --place_report_in_output_folder --
default_min_aln_score 60 -q 0 -s 0 --plot_window_size 20 --min_bp_quality_or_N 0 --
exclude_bp_from_left 15 --exclude_bp_from_right 15 --conversion_nuc_from C --
conversion_nuc_to T --prime_editing_pegRNA_extension_quantification_window_size 5 -w 1 -
wc -3
```

Parameters:

```
allele_plot_pcts_only_for_assigned_reference: False
aln_seed_count: 5
aln_seed_len: 10
aln_seed_min: 2
amplicon_min_alignment_score:
amplicon_name: Reference
amplicon_seq:
TGAGGAAGAAGAAAATATTAGTTACCTAAATGAGAGTTCTGGGGAAGAGTGGGATTCCTCTGAAGAAGAGGACTCTATGGTGCCCAACT
TATCGCCTCTTGAGAGTCTTGCCTGGCAGGTTAAGTGCCTTTTAAAATATTCCACAACTTGGAAACCTTTAAATCCTAATTCCTGGTTG
TATCATGCTAAACTGTTGGATC
annotate_wildtype_allele:
auto: False
bam_chr_loc:
bam_input:
base_editor_output: False
coding_seq:
```

Running log

Allele assignments

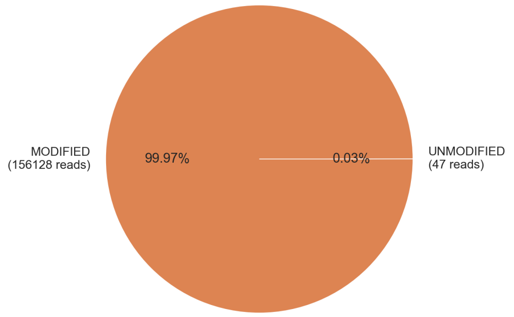

Figure 1b: Alignment and editing frequency of reads as determined by the percentage and number of sequence reads showing unmodified and modified alleles.

Data: Quantification of editing

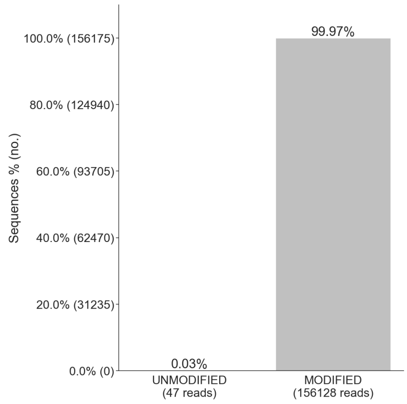

Figure 1c: Alignment and editing frequency of reads as determined by the percentage and number of sequence reads showing unmodified and modified alleles.

Data: [Quantification of editing]

Nucleotide composition

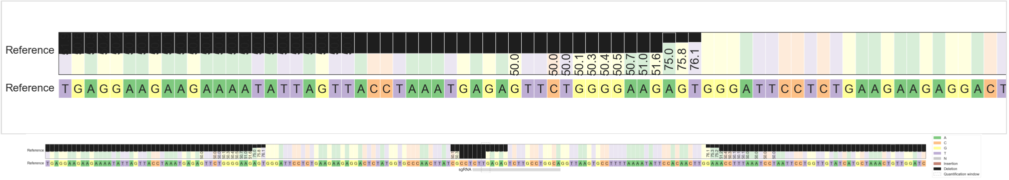

Hover your mouse over the bottom image to zoom in on a specific region.

Figure 2a: Nucleotide distribution across amplicon. At each base in the reference amplicon, the percentage of each base as observed in sequencing reads is shown (A = green; C = orange; G = yellow; T = purple). Black bars show the percentage of reads for which that base was deleted. Brown bars between bases show the percentage of reads having an insertion at that position.

Data: [Nucleotide frequency table]

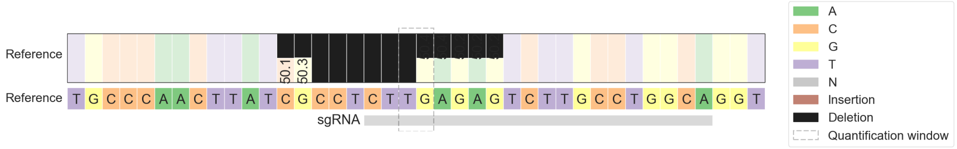

Figure 2b: Nucleotide distribution around the sgRNA TGCCAGGCAAGACTCTCAAG.

Data: [Nucleotide frequency in quantification window]

Modification lengths

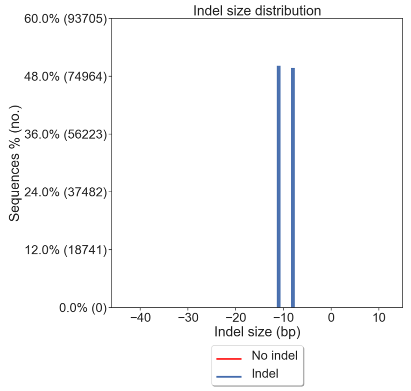

Figure 3a: Frequency distribution of alleles with indels (blue) and without indels (red). Note that histograms are clipped to show 99% of the data. To show all data, run using the parameter '--plot_histogram_outliers'. (Minimum -69 not shown)

Data: Indel histogram

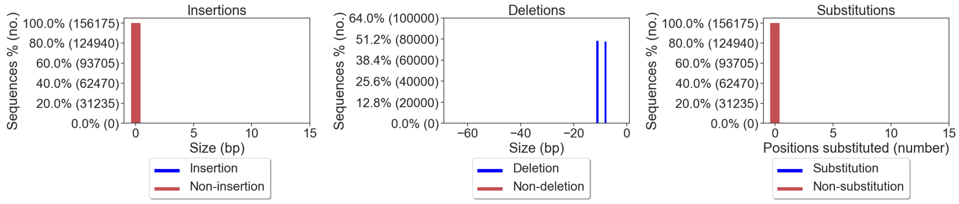

Figure 3b: Left panel, frequency distribution of sequence modifications that increase read length with respect to the reference amplicon, classified as insertions (positive indel size). Middle panel, frequency distribution of sequence modifications that reduce read length with respect to the reference amplicon, classified as deletions (negative indel size). Right panel, frequency distribution of sequence modifications that do not alter read length with respect to the reference amplicon, which are classified as substitutions (number of substituted positions shown).

Data: Insertions frequency

Data: Deletions Frequency

Data: Substitutions Frequency

Indel characterization

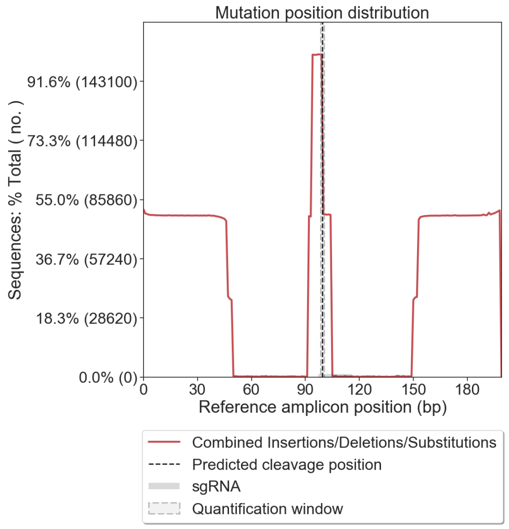

Figure 4a: Combined frequency of any modification across the amplicon. Modifications outside of the quantification window are also shown.

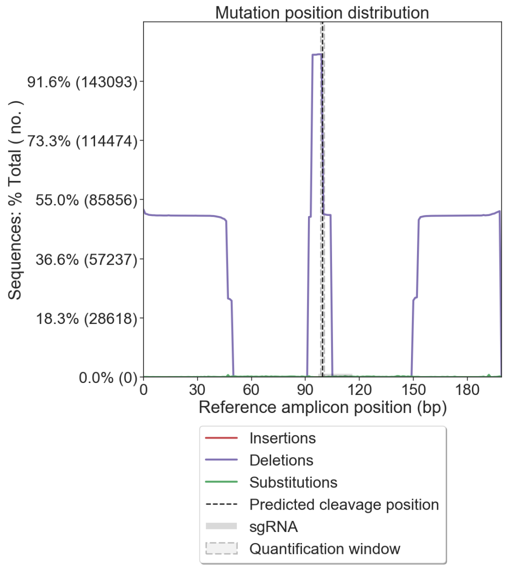

Figure 4b: Frequency of insertions (red), deletions (purple), and substitutions (green) across the entire amplicon, including modifications outside of the quantification window.

Data: Modification frequency

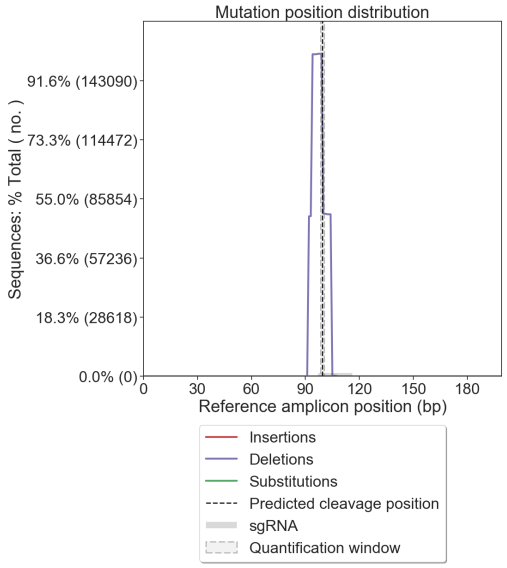

Figure 4c: Frequency of insertions (red), deletions (purple), and substitutions (green) across the entire amplicon, considering only modifications that overlap with the quantification window.

Data: Modification frequency in quantification window

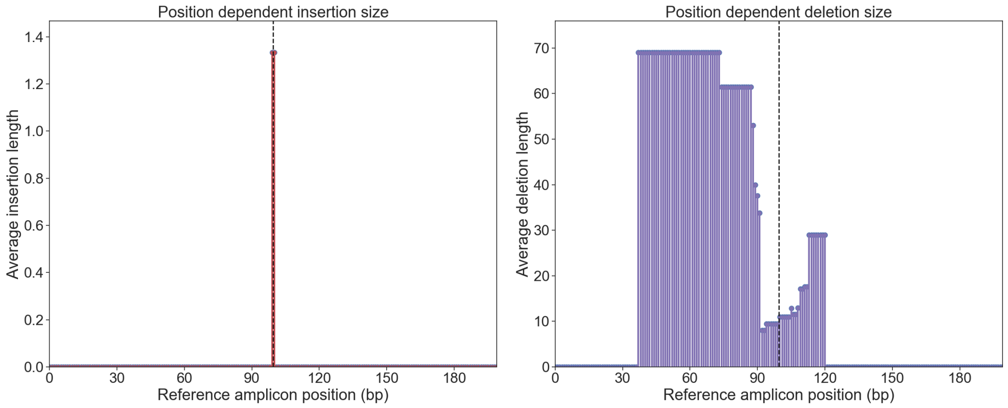

Figure 4d: Position dependent insertion size(left) and deletion size (right), including only modifications that overlap with the quantification window.

Allele plots

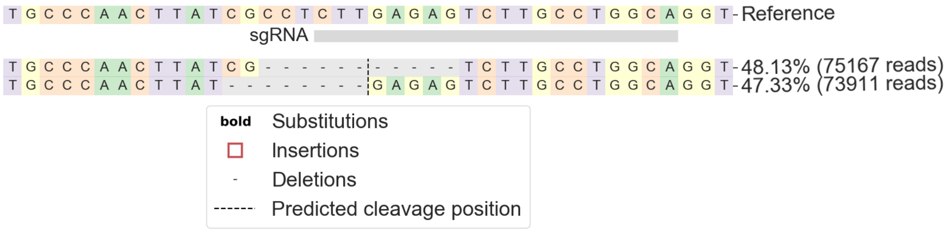

Figure 9: Visualization of the distribution of identified alleles around the cleavage site for the sgRNA TGCCAGGCAAGACTCTCAAG. Nucleotides are indicated by unique colors (A = green; C = red; G = yellow; T = purple). Substitutions are shown in bold font. Red rectangles highlight inserted sequences. Horizontal dashed lines indicate deleted sequences. The vertical dashed line indicates the predicted cleavage site.

Data: Allele frequency table

Download report | Link to report | Print

If you like CRISPResso2 please support us by citing it in your work:
Clement K, Rees H, Canver MC, Gehrke JM, Farouni R, Hsu JY, Cole MA, Liu DR, Joung JK, Bauer DE, Pinello L.
CRISPResso2 provides accurate and rapid genome editing sequence analysis.
Nat Biotechnol. 2019 Mar; 37(3):224-226. doi: 10.1038/s41587-019-0032-3. PubMed PMID: 30809026.

© Copyright Kendell Clement and Luca Pinello

Tweet

CRISPResso2

Analysis of genome editing outcomes from deep sequencing data

CRISPResso2 run information

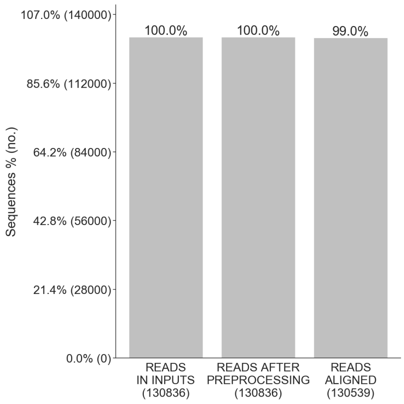

Figure 1a: The number of reads in input fastqs, after preprocessing, and after alignment to amplicons.

Data: Mapping statistics

CRISPResso version: 2.0.45

Run completed: 2021-04-03 19:54:38

Amplicon sequence:

```
ATGGTGCCCAACTTATCGCCTCTTGAGAGTCTTGCCTGGCAGGTTAAGTGCCTTTTAAAATATTCCACAACTTGGAAACCTTTAAATCC
TAATTCCTGGTTGTATCATGCTAAACTGTTGGATCCAAGCACACCAGTCCATATACTTCGAGAGATAGGTCTAAGACTCTCCCATTGTT
CCCATTGTGTCCCCAAACTGGA
```

Guide sequence:

```
CAGTTTAGCATGATACAACC
```

Command used:

```
CRISPResso -r1 CRISPResso_Input_Reads_f9daf084-dd84-4847-86b2-c6fc7e24bb5e.fastq -a
ATGGTGCCCAACTTATCGCCTCTTGAGAGTCTTGCCTGGCAGGTTAAGTGCCTTTTAAAATATTCCACAACTTGGAAACCTTTAAATCC
TAATTCCTGGTTGTATCATGCTAAACTGTTGGATCCAAGCACACCAGTCCATATACTTCGAGAGATAGGTCTAAGACTCTCCCATTGTT
CCCATTGTGTCCCCAAACTGGA -g CAGTTTAGCATGATACAACC --name MHiDRT --output_folder
CRISPRessoRunMHiDRT --write_cleaned_report --place_report_in_output_folder --
default_min_aln_score 60 -q 0 -s 0 --plot_window_size 20 --min_bp_quality_or_N 0 --
exclude_bp_from_left 15 --exclude_bp_from_right 15 --conversion_nuc_from C --
conversion_nuc_to T --prime_editing_pegRNA_extension_quantification_window_size 5 -w 1 -
wc -3
```

Parameters:

```
allele_plot_pcts_only_for_assigned_reference: False
aln_seed_count: 5
aln_seed_len: 10
aln_seed_min: 2
amplicon_min_alignment_score:
amplicon_name: Reference
amplicon_seq:
ATGGTGCCCAACTTATCGCCTCTTGAGAGTCTTGCCTGGCAGGTTAAGTGCCTTTTAAAATATTCCACAACTTGGAAACCTTTAAATCC
TAATTCCTGGTTGTATCATGCTAAACTGTTGGATCCAAGCACACCAGTCCATATACTTCGAGAGATAGGTCTAAGACTCTCCCATTGTT
CCCATTGTGTCCCCAAACTGGA
annotate_wildtype_allele:
auto: False
bam_chr_loc:
bam_input:
base_editor_output: False
coding_seq:
```

Running log

Allele assignments

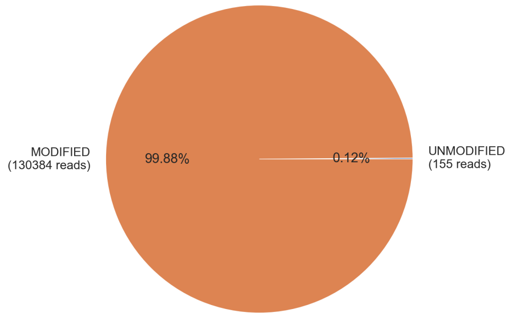

Figure 1b: Alignment and editing frequency of reads as determined by the percentage and number of sequence reads showing unmodified and modified alleles.

Data: Quantification of editing

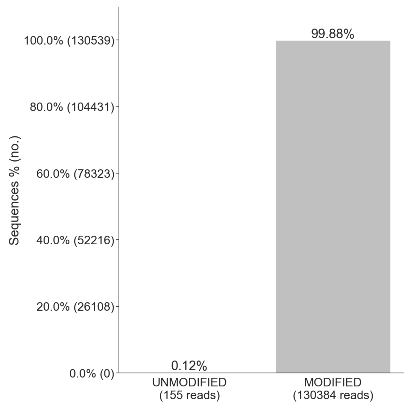

Figure 1c: Alignment and editing frequency of reads as determined by the percentage and number of sequence reads showing unmodified and modified alleles.

Data: [Quantification of editing]

Nucleotide composition

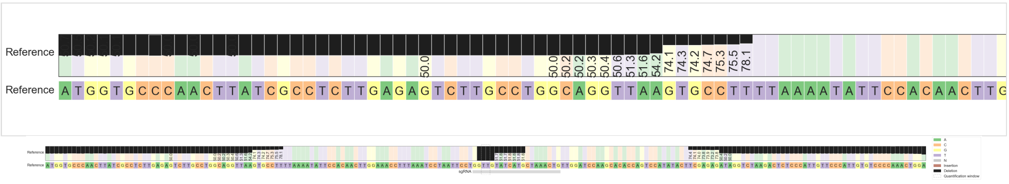

Hover your mouse over the bottom image to zoom in on a specific region.

Figure 2a: Nucleotide distribution across amplicon. At each base in the reference amplicon, the percentage of each base as observed in sequencing reads is shown (A = green; C = orange; G = yellow; T = purple). Black bars show the percentage of reads for which that base was deleted. Brown bars between bases show the percentage of reads having an insertion at that position.

Data: [Nucleotide frequency table]

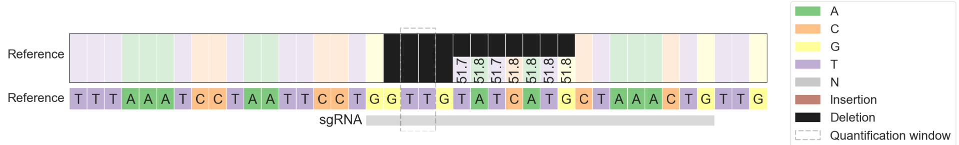

Figure 2b: Nucleotide distribution around the sgRNA CAGTTTAGCATGATACAACC.

Data: [Nucleotide frequency in quantification window]

Modification lengths

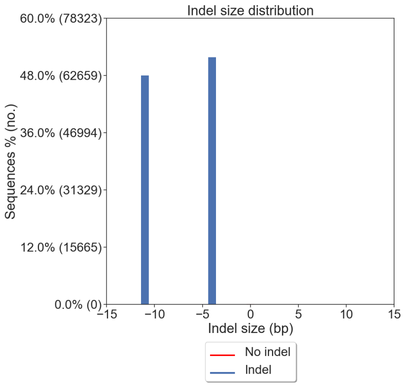

Figure 3a: Frequency distribution of alleles with indels (blue) and without indels (red). Note that histograms are clipped to show 99% of the data. To show all data, run using the parameter '--plot_histogram_outliers'. (Minimum -33 not shown)

Data: Indel histogram

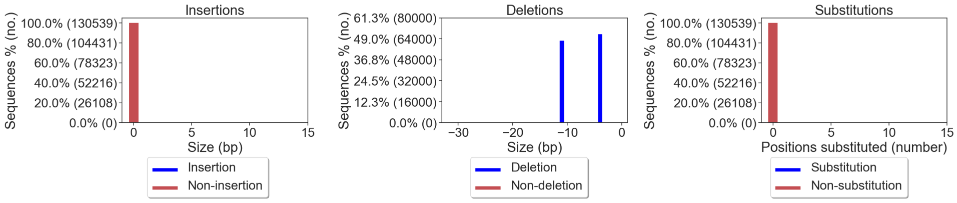

Figure 3b: Left panel, frequency distribution of sequence modifications that increase read length with respect to the reference amplicon, classified as insertions (positive indel size). Middle panel, frequency distribution of sequence modifications that reduce read length with respect to the reference amplicon, classified as deletions (negative indel size). Right panel, frequency distribution of sequence modifications that do not alter read length with respect to the reference amplicon, which are classified as substitutions (number of substituted positions shown).

Data: Insertions frequency

Data: Deletions Frequency

Data: Substitutions Frequency

Indel characterization

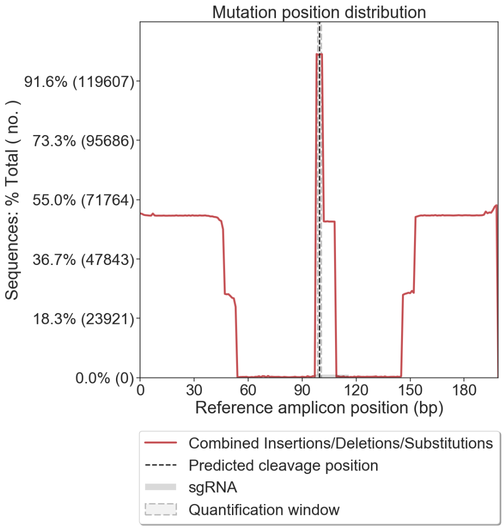

Figure 4a: Combined frequency of any modification across the amplicon. Modifications outside of the quantification window are also shown.

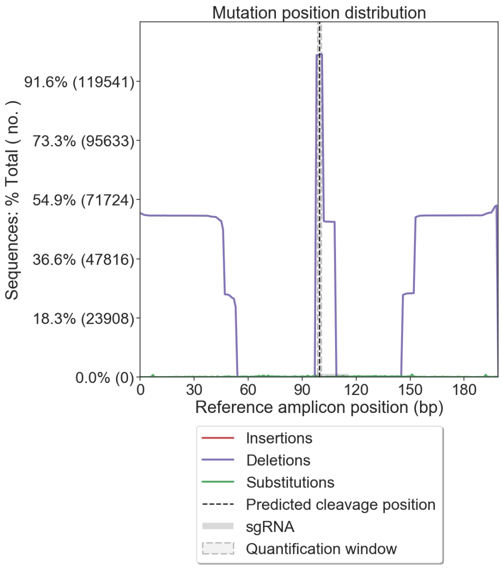

Figure 4b: Frequency of insertions (red), deletions (purple), and substitutions (green) across the entire amplicon, including modifications outside of the quantification window.

Data: Modification frequency

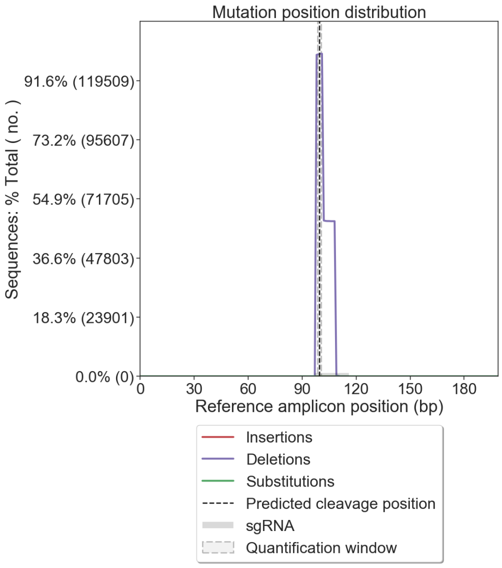

Figure 4c: Frequency of insertions (red), deletions (purple), and substitutions (green) across the entire amplicon, considering only modifications that overlap with the quantification window.

Data: Modification frequency in quantification window

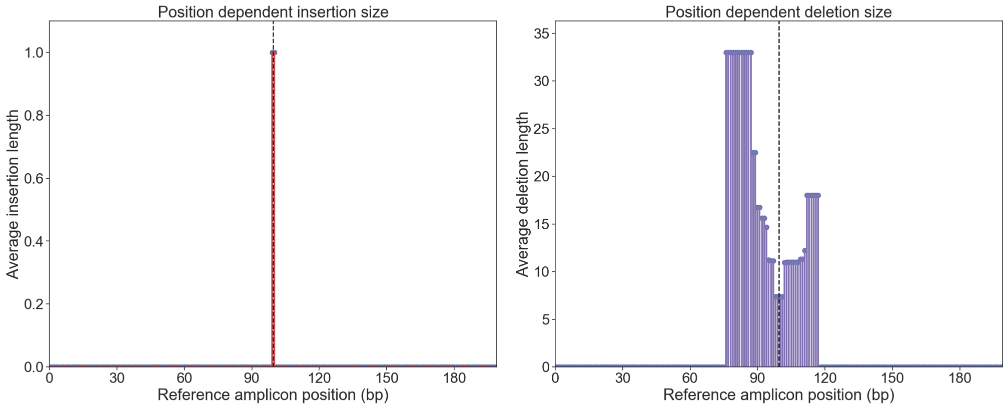

Figure 4d: Position dependent insertion size(left) and deletion size (right), including only modifications that overlap with the quantification window.

Allele plots

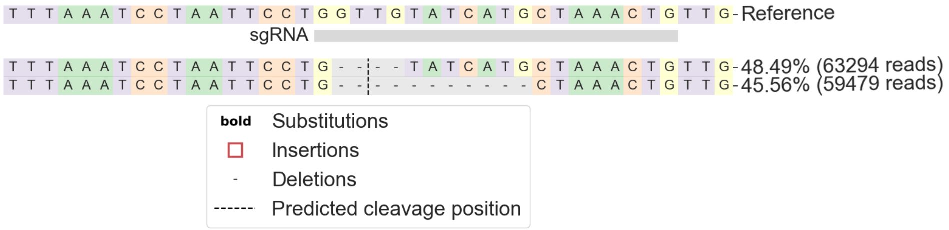

Figure 9: Visualization of the distribution of identified alleles around the cleavage site for the sgRNA CAGTTTAGCATGATACAACC. Nucleotides are indicated by unique colors (A = green; C = red; G = yellow; T = purple). Substitutions are shown in bold font. Red rectangles highlight inserted sequences. Horizontal dashed lines indicate deleted sequences. The vertical dashed line indicates the predicted cleavage site.

Data: Allele frequency table

Download report | Link to report | Print

If you like CRISPResso2 please support us by citing it in your work:
Clement K, Rees H, Canver MC, Gehrke JM, Farouni R, Hsu JY, Cole MA, Liu DR, Joung JK, Bauer DE, Pinello L.
CRISPResso2 provides accurate and rapid genome editing sequence analysis.
Nat Biotechnol. 2019 Mar; 37(3):224-226. doi: 10.1038/s41587-019-0032-3. PubMed PMID: 30809026.

© Copyright Kendell Clement and Luca Pinello

Tweet
