# Supplementary material for: Template-assisted covalent modification underlies activity of covalent molecular glues
Source: Nat Chem Biol. 2024 Jul 29;20(12):1640–9. doi: 10.1038/s41589-024-01668-4 (PMC11582070; doi:10.1038/s41589-024-01668-4)
Supplement: Supplementary file 2 — Reporting Summary [file 41589_2024_1668_MOESM2_ESM.pdf]

nature portfolio

Corresponding author(s): Benjamin L. Ebert, Eric S. Fischer, and Nathanael S. Gray

Last updated by author(s): Apr 30, 2024

Reporting Summary

Nature Portfolio wishes to improve the reproducibility of the work that we publish. This form provides structure for consistency and transparency in reporting. For further information on Nature Portfolio policies, see our Editorial Policies and the Editorial Policy Checklist.

Statistics

For all statistical analyses, confirm that the following items are present in the figure legend, table legend, main text, or Methods section.

n/a	Confirmed	
☐	☒	The exact sample size (*n*) for each experimental group/condition, given as a discrete number and unit of measurement
☐	☒	A statement on whether measurements were taken from distinct samples or whether the same sample was measured repeatedly
☐	☒	The statistical test(s) used AND whether they are one- or two-sided<br>*Only common tests should be described solely by name; describe more complex techniques in the Methods section.*
☒	☐	A description of all covariates tested
☒	☐	A description of any assumptions or corrections, such as tests of normality and adjustment for multiple comparisons
☐	☒	A full description of the statistical parameters including central tendency (e.g. means) or other basic estimates (e.g. regression coefficient) AND variation (e.g. standard deviation) or associated estimates of uncertainty (e.g. confidence intervals)
☐	☒	For null hypothesis testing, the test statistic (e.g. *F*, *t*, *r*) with confidence intervals, effect sizes, degrees of freedom and *P* value noted<br>*Give P values as exact values whenever suitable.*
☒	☐	For Bayesian analysis, information on the choice of priors and Markov chain Monte Carlo settings
☒	☐	For hierarchical and complex designs, identification of the appropriate level for tests and full reporting of outcomes
☒	☐	Estimates of effect sizes (e.g. Cohen's *d*, Pearson's *r*), indicating how they were calculated

Our web collection on statistics for biologists contains articles on many of the points above.

Software and code

Policy information about availability of computer code

Data collection

Western blot data were imaged on the Odyssey Imaging System with Image Studio software (LI-COR).
Flow data were collected on BD LSRFortessa Cell Analyzer with BD FACSDiva 8.0 software (BD Biosciences).
TR-FRET data were acquired on a PHERAstar FS microplate reader (BMG Labtech).
Proteomics data were collected using a TimsTOF Pro2 (Bruker Daltonics) and an Orbitrap Exploris 480 mass spectrometer (Thermo Fisher Scientific).
Intact MS data were collected using a LTQ ion trap mass spectrometer (Thermo Fisher Scientific) or Orbitrap Eclipse mass spectrometer (Thermo Scientific).
AlphaScreen data were acquired on an EnVision 2104 Multilabel Plate Readers (PerkinElmer)
Cryo movies were collected on a Titan Krios Transmission Electron Microscope (Thermo Fisher Scientific).

Data analysis

CRISPR screen data analysis and visualization were done using the R programming language and RStudio with the following packages: tidyverse (version 1.3.0.9000), ggrepel (version 0.8.2), GGally (version 2.0.0), dr4pl (version 1.1.11), ShortRead (version 1.44.3), and Limma (version 3.42.2). Custom R scripts used to analyze and visualize the data are provided in Supplementary Code.
Flow data were analyzed with FlowJo v10 (BD Biosciences), and following data analysis and visualization were done using the R programming language and RStudio with tidyverse (version 1.3.0.9000) and dr4pl (version 1.1.11) packages. Custom R scripts used to analyze and visualize the data are provided in Supplementary Code.
Proteomics data were analyzed with Proteome Discoverer 2.4 or 2.5 (Thermo Fisher Scientific): RRID:SCR_014477 and DIA-NN 1.8. Analysis and visualization were done using the R programming language and RStudio with the following packages: tidyverse (version 1.3.0.9000) and Limma (version 3.42.2). Custom R scripts used to analyze and visualize the data are provided in Supplementary Code.
Intact MS data were analyzed with MagTran (version 1.03 b2) or UniDec (version 6.0.4).
Structural data from cryo-EM were processed with cryoSPARC v3.3.2, Topaz v0.2.5a, deepEMhancer v0.16, COOT v0.9.8, ChimeraX v1.4 ,

ISOLDE v1.3, AceDRG v5.8.0091, Refmac5 v5.8.0091, Phenix.real_space_refine v.1.19.2-4158, PDBePisa v1.52, PDBeFold, 3DFSC v3.0 and SBGrid.

For manuscripts utilizing custom algorithms or software that are central to the research but not yet described in published literature, software must be made available to editors and reviewers. We strongly encourage code deposition in a community repository (e.g. GitHub). See the Nature Portfolio guidelines for submitting code & software for further information.

Data

Policy information about availability of data

All manuscripts must include a data availability statement. This statement should provide the following information, where applicable:

- Accession codes, unique identifiers, or web links for publicly available datasets
- A description of any restrictions on data availability
- For clinical datasets or third party data, please ensure that the statement adheres to our policy

Cryo-EM maps and coordinates have been deposited in the EMDB and PDB, under accession codes EMD-29714 and 8G46 respectively. Raw data files of whole-cell proteome mass spectrometry, IP-MS, and Biotin pull-down mass spectrometry in this study have been deposited in the PRIDE Archive, including PXD047137, PXD047138, PXD047141 and PXD051457. Intact mass spectrometry raw data related to Fig. 2b, 4e, Extended Data Fig. 2d, 2e, 4e, 4f, 7f are available for free download at: ftp://massive.ucsd.edu/MSV000093731. Synthetic procedures of JQ1-derived compounds, schematics of sorting strategies, and deep sequencing results for DCAF16 knockout clones are provided in Supplementary Information. Coding sequences of the DNA constructs used in this study and mammalian cell lines authentication results are provided as Supplementary Table. Source Data are provided with this paper.

Human research participants

Policy information about studies involving human research participants and Sex and Gender in Research.

Reporting on sex and gender	NA
Population characteristics	NA
Recruitment	NA
Ethics oversight	NA

Note that full information on the approval of the study protocol must also be provided in the manuscript.

Field-specific reporting

Please select the one below that is the best fit for your research. If you are not sure, read the appropriate sections before making your selection.

☒ Life sciences ☐ Behavioural & social sciences ☐ Ecological, evolutionary & environmental sciences

For a reference copy of the document with all sections, see nature.com/documents/nr-reporting-summary-flat.pdf

Life sciences study design

All studies must disclose on these points even when the disclosure is negative.

Sample size	No sample size calculation was performed. The sample size (n) of each experiment is provided in the figure legends. The sample size was chosen to allow the assessment of reproducibility and the conduct of statistical hypothesis testing.
Data exclusions	No data was excluded from the analysis.
Replication	Number of replicates is described in the figure legends, where applicable.
Randomization	No randomization was performed since no risk of errors associated with systematic selection bias was expected.
Blinding	Investigators were not blinded during data collection or analysis. However, controls and samples were analyzed in exactly the same way using the same computational pipeline.

Reporting for specific materials, systems and methods

We require information from authors about some types of materials, experimental systems and methods used in many studies. Here, indicate whether each material, system or method listed is relevant to your study. If you are not sure if a list item applies to your research, read the appropriate section before selecting a response.

Materials & experimental systems

n/a	Involved in the study
☐	☒ Antibodies
☐	☒ Eukaryotic cell lines
☒	☐ Palaeontology and archaeology
☒	☐ Animals and other organisms
☒	☐ Clinical data
☒	☐ Dual use research of concern

Methods

n/a	Involved in the study
☒	☐ ChIP-seq
☐	☒ Flow cytometry
☒	☐ MRI-based neuroimaging

Antibodies

Antibodies used

The following antibodies were used:
• anti-BRD4 (Bethyl Laboratories, Cat# A301-985A100, Polyclonal, 1:1000 dilution)
• anti-β-actin (Cell Signaling Technology, Cat# 3700, Clone 8H10D10, 1:10000 dilution)
• anti-Flag (Sigma-Aldrich, Cat# F1804, Clone M2, 1:1000 dilution)
• anti-HA (Cell Signaling Technology, Cat# 3724, Clone C29F4, 1:1000 dilution)
• IRDye 800CW Goat anti-Mouse IgG Secondary Antibody (LI-COR Biosciences, Cat# 926-32210, 1:10000 dilution)
• IRDye 680LT Goat anti-Rabbit IgG Secondary Antibody (LI-COR Biosciences, Cat# 926-68021, 1:10000 dilution)

Validation

All antibodies are commercially available and were validated by their manufacturer. See link below for validation data and previous works that have used the same antibody:
• anti-BRD4: https://www.thermofisher.com/antibody/product/BRD4-Antibody-Polyclonal/A301-985A100
• anti-β-actin: https://www.cellsignal.com/products/primary-antibodies/b-actin-8h10d10-mouse-mab/3700
• anti-Flag: https://www.sigmaaldrich.com/US/en/product/sigma/f3165
• anti-HA: https://www.cellsignal.com/products/primary-antibodies/ha-tag-c29f4-rabbit-mab/3724
• IRDye 800CW Goat anti-Mouse IgG Secondary Antibody: https://www.licor.com/bio/reagents/irdye-800cw-goat-anti-mouse-igg-secondary-antibody
• IRDye 680LT Goat anti-Rabbit IgG Secondary Antibody: https://www.licor.com/bio/reagents/irdye-680lt-goat-anti-rabbit-igg-secondary-antibody

Eukaryotic cell lines

Policy information about cell lines and Sex and Gender in Research

Cell line source(s)

The human HEK293T and HEK293T-Cas9 cell lines were provided by the Genetic Perturbation Platform, Broad Institute. K562-Cas9 cell line was provided by Zuzana Tothova (Dana-Farber Cancer Institute). Spodoptera frugiperda (Sf9) insect cells were purchased from Expression Systems (Cat# 94-001F). Trichoplusia ni High Five insect insect cells were purchased from Thermo Fisher Scientific (Cat# 85502).

Authentication

HEK293T, HEK293T-Cas9, and K562-Cas9 cell lines were authenticated by STR profiling.

Mycoplasma contamination

Mycoplasma negative.

Commonly misidentified lines
(See ICLAC register)

None of commonly misidentified lines were used in this study.

Flow Cytometry

Plots

Confirm that:

☒ The axis labels state the marker and fluorochrome used (e.g. CD4-FITC).

☒ The axis scales are clearly visible. Include numbers along axes only for bottom left plot of group (a 'group' is an analysis of identical markers).

☒ All plots are contour plots with outliers or pseudocolor plots.

☒ A numerical value for number of cells or percentage (with statistics) is provided.

Methodology

Sample preparation

Adherent cells were trypsinized, collected, and the cell pellets resuspended in PBS. Suspension cells were washed with PBS or directly subjected to analysis without fixation.

Instrument

MA900 Cell Sorter (Sony) for sorting screens, and BD LSRFortessa Cell Analyzer (BD Biosciences) for flow analysis.

Software

BD FACSDiva 8.0 (BD Biosciences), FlowJo 10 (BD Biosciences).

Cell population abundance	Round cells (population with forward and side scatter properties consistent with the alive, non-treated cell line) were usually > 50% in most measurements (rarely lower due to drug toxicity), singlets were > 90%. For reporter assays, mCherry positive cells > 50%.
Gating strategy	Cells were first gated for live cells based on forward and side scatter. Single cells were discriminated based on the area vs. height of the side scatter. Finally, reporter positive cells were gated based on the mCherry expression.

☒ Tick this box to confirm that a figure exemplifying the gating strategy is provided in the Supplementary Information.
