# Supplementary material for: Template-assisted covalent modification underlies activity of covalent molecular glues
Source: Nat Chem Biol. 2024 Jul 29;20(12):1640–9. doi: 10.1038/s41589-024-01668-4 (PMC11582070; doi:10.1038/s41589-024-01668-4)
Supplement: Supplementary file 11 — Uncropped western blot. [file 41589_2024_1668_MOESM11_ESM.pdf]

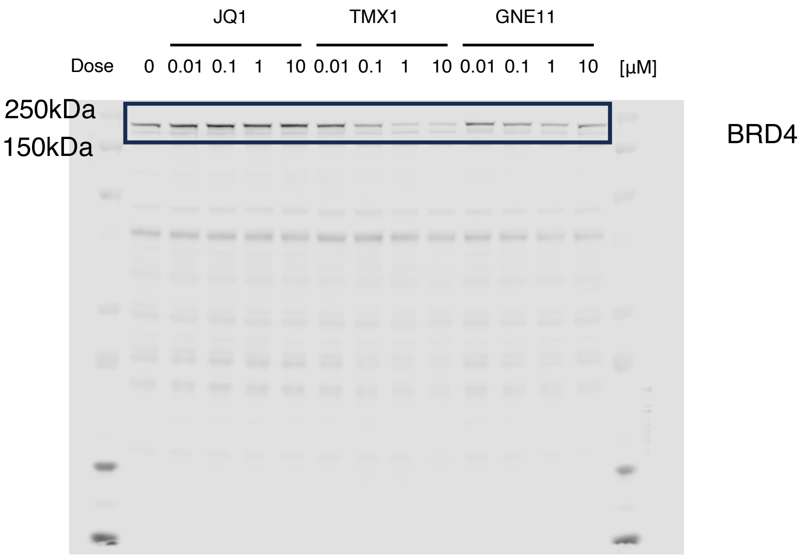

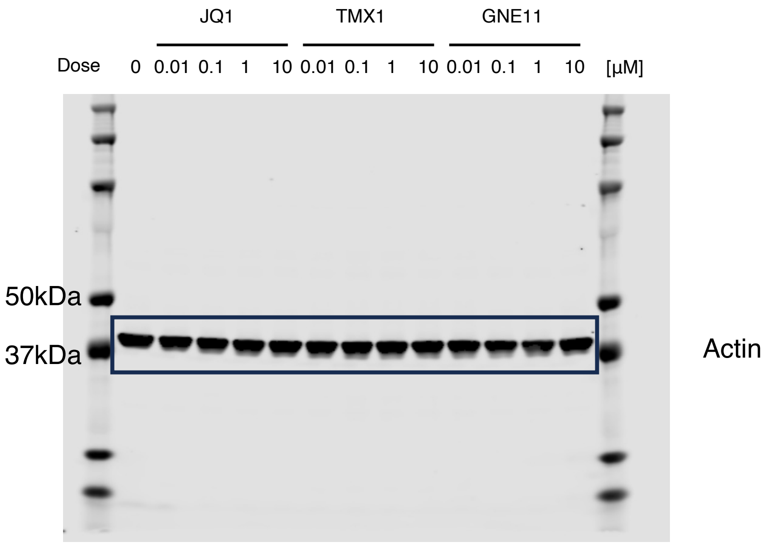

Related to Fig. 1b
BRD4 and Actin blots were run on the same gel

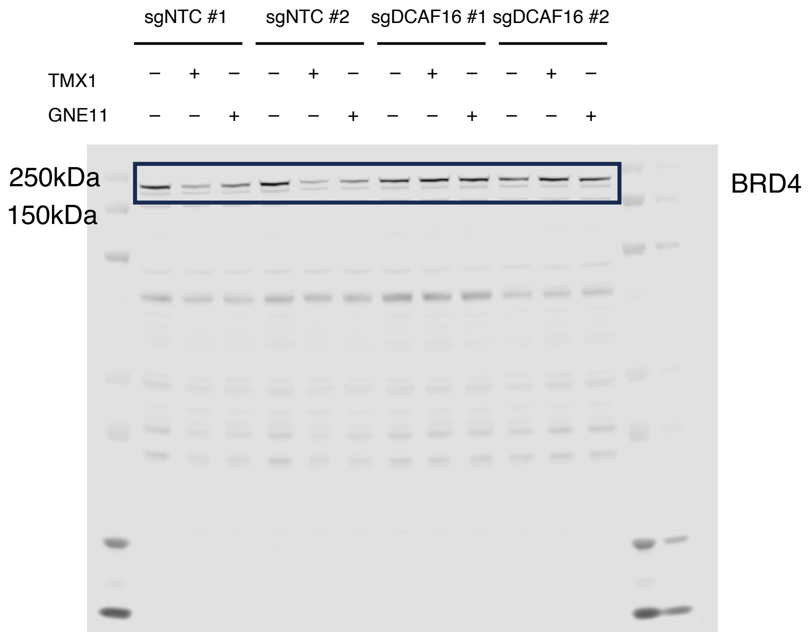

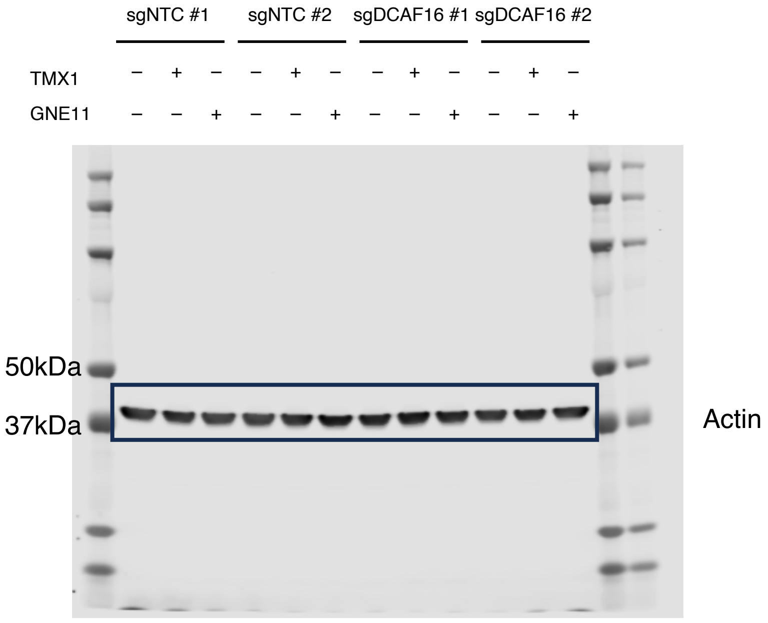

Related to Fig. 1e

BRD4 and Actin blots were run on the same gel
