# Supplementary figures and images for: Template-assisted covalent modification underlies activity of covalent molecular glues
Source: Nat Chem Biol. 2024 Jul 29;20(12):1640–9. doi: 10.1038/s41589-024-01668-4 (PMC11582070; doi:10.1038/s41589-024-01668-4)

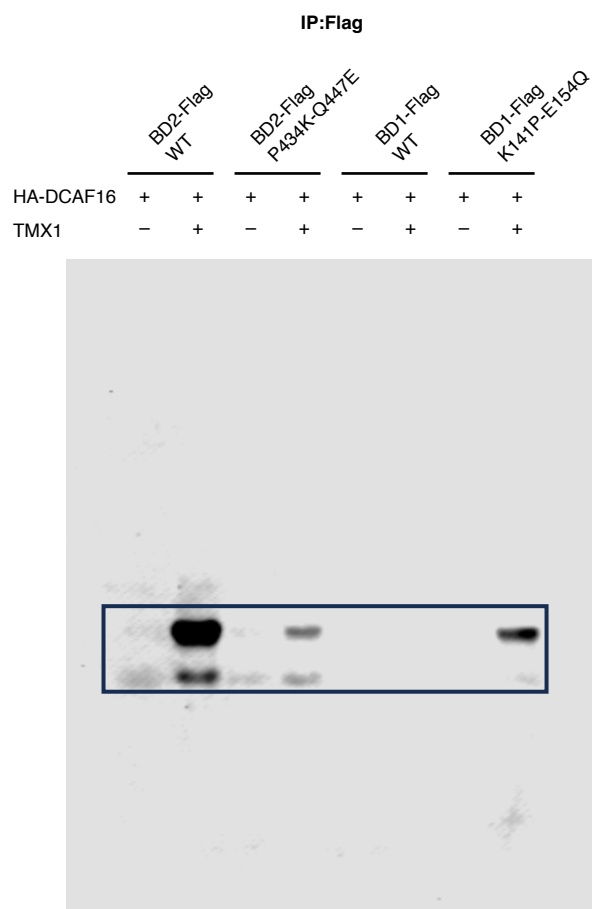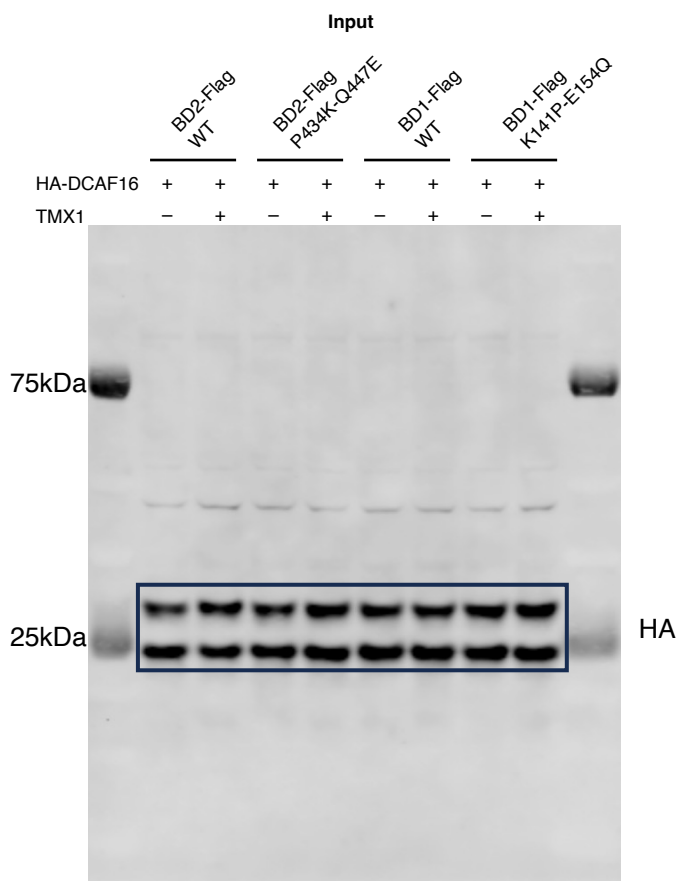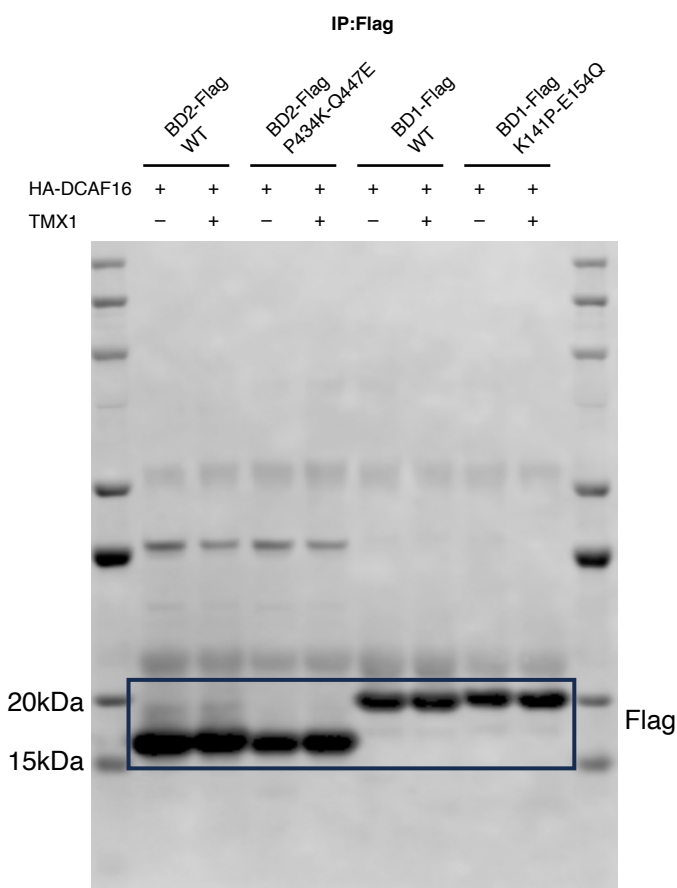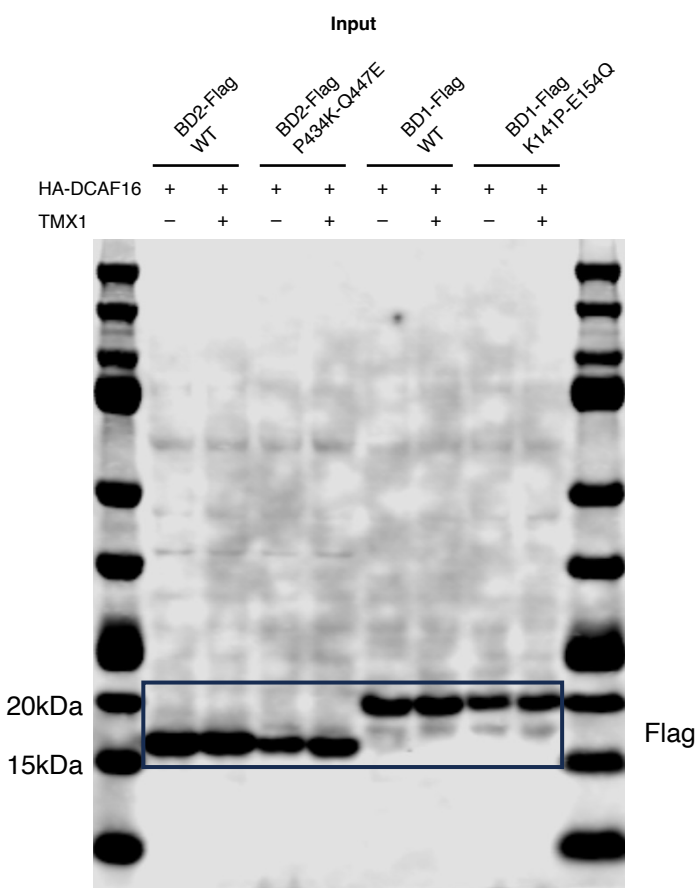

Related to Fig. 5d

IP blots were run on gel 1; Input blots were run on gel 2

Supplement: Supplementary file 17 — Uncropped western blot. [file 41589_2024_1668_MOESM17_ESM.pdf]

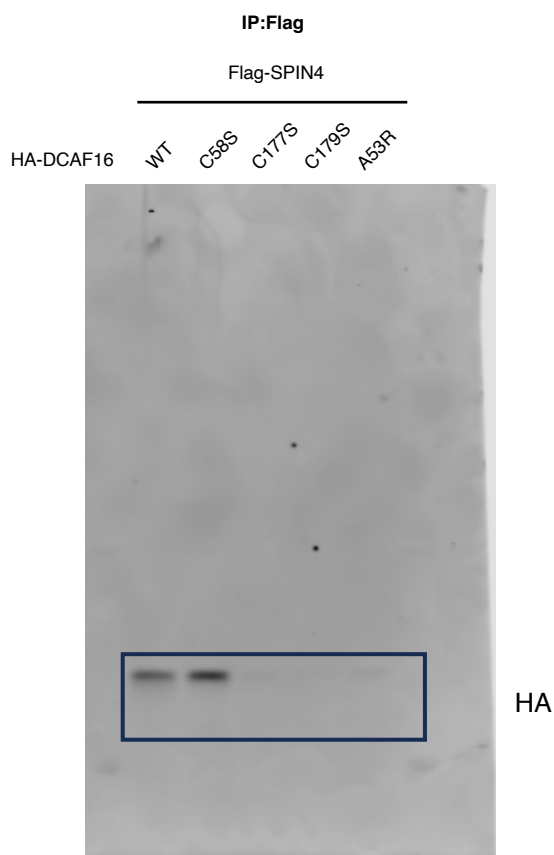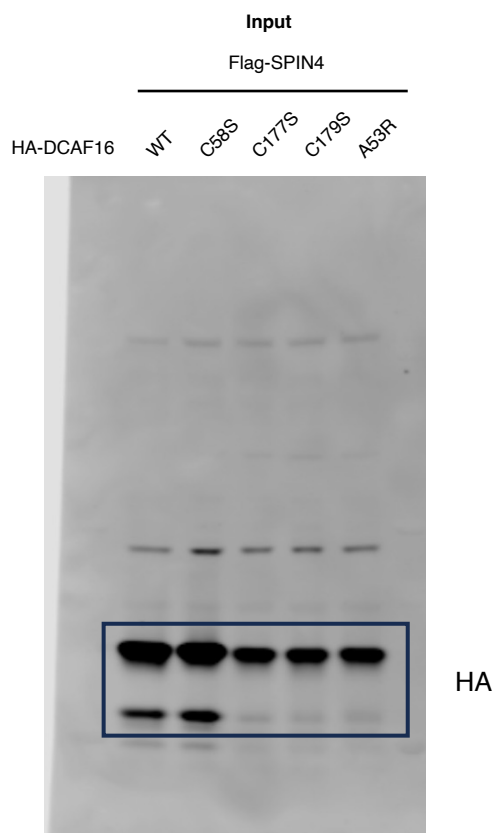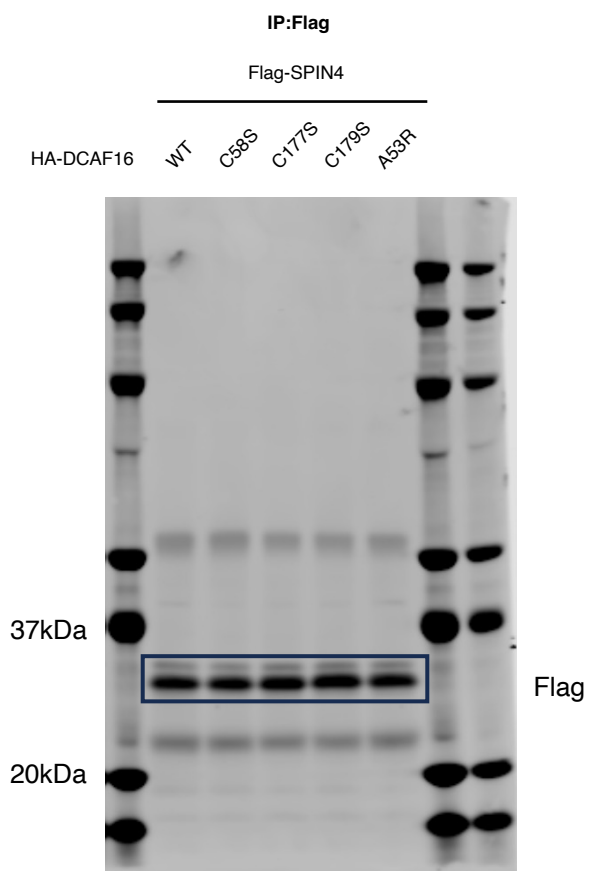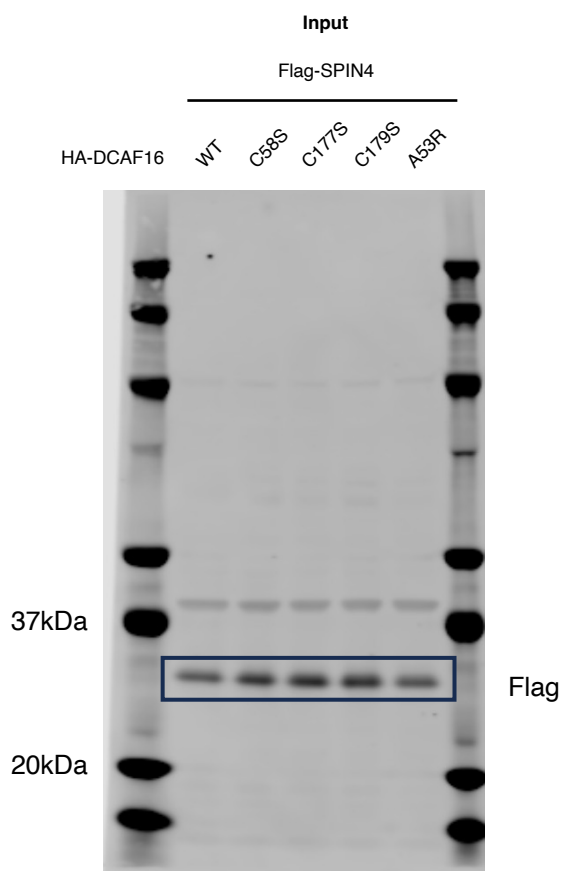

Related to Extended Data Fig. 7c

IP blots were run on gel 1; Input blots were run on gel 2

Supplement: Supplementary file 27 — Uncropped western blot. [file 41589_2024_1668_MOESM27_ESM.pdf]
