# Supplementary material for: Template-assisted covalent modification underlies activity of covalent molecular glues
Source: Nat Chem Biol. 2024 Jul 29;20(12):1640–9. doi: 10.1038/s41589-024-01668-4 (PMC11582070; doi:10.1038/s41589-024-01668-4)
Supplement: Supplementary file 19 — Uncropped western blot and raw data for intact MS. [file 41589_2024_1668_MOESM19_ESM.pdf]

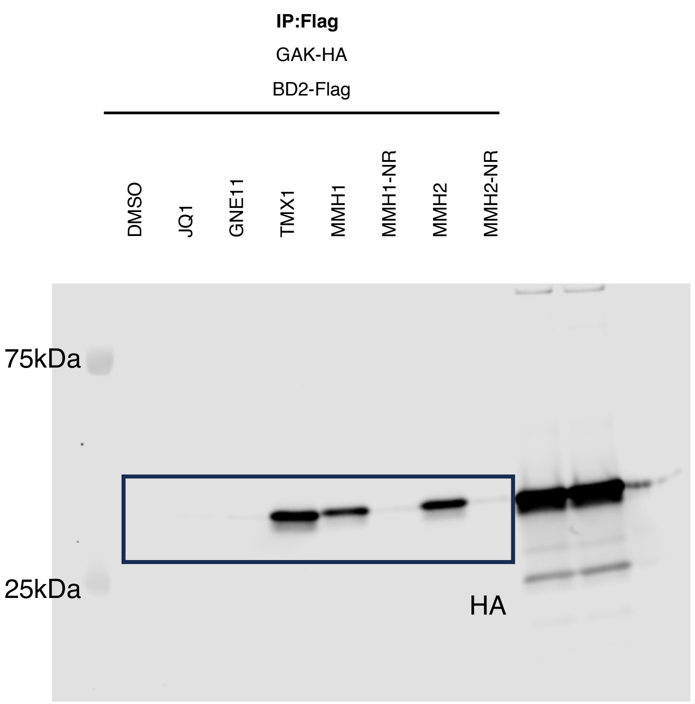

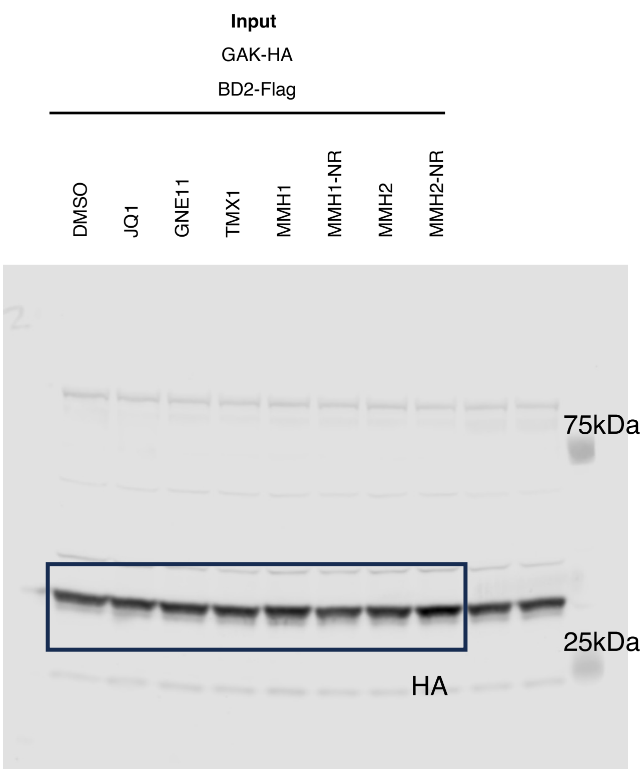

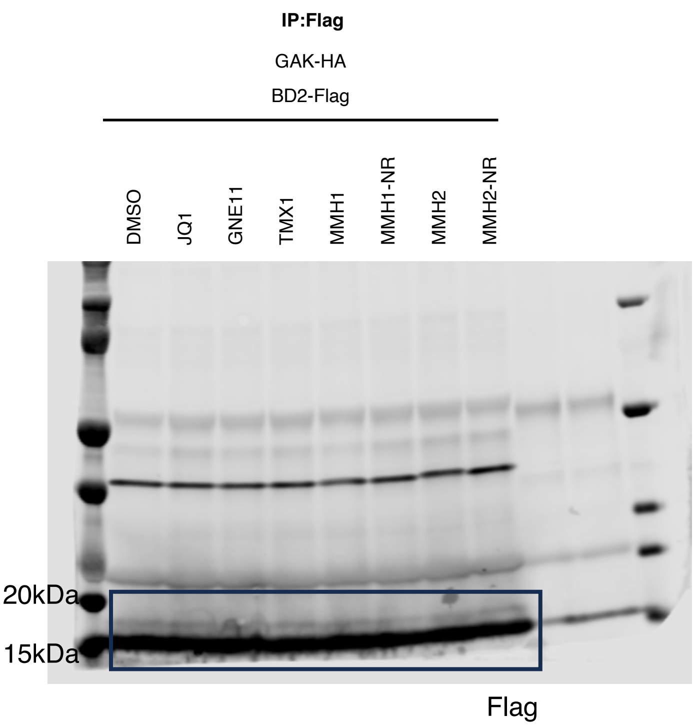

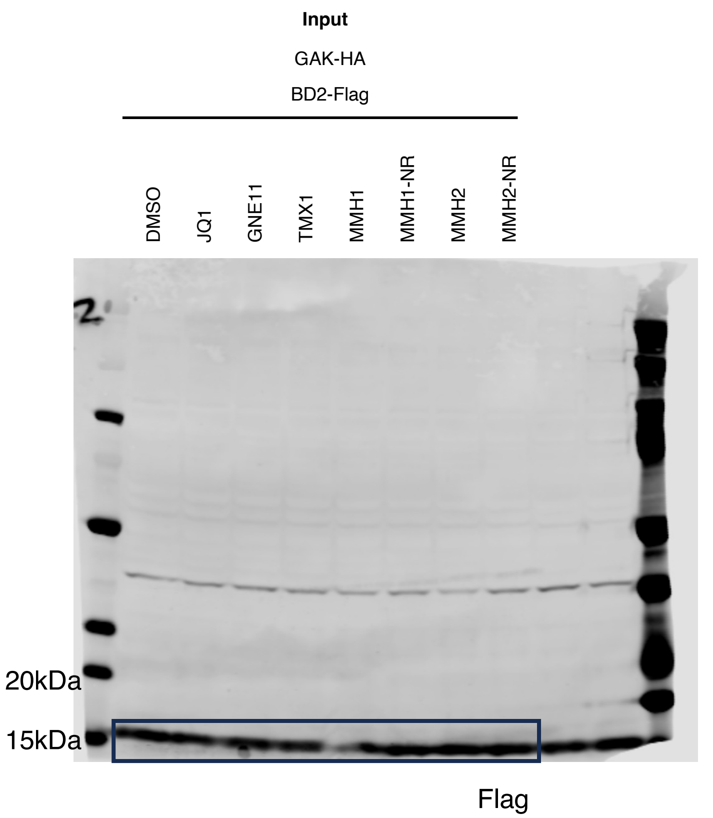

Related to Fig. 6a

IP blots were run on gel 1; Input blots were run on gel 2

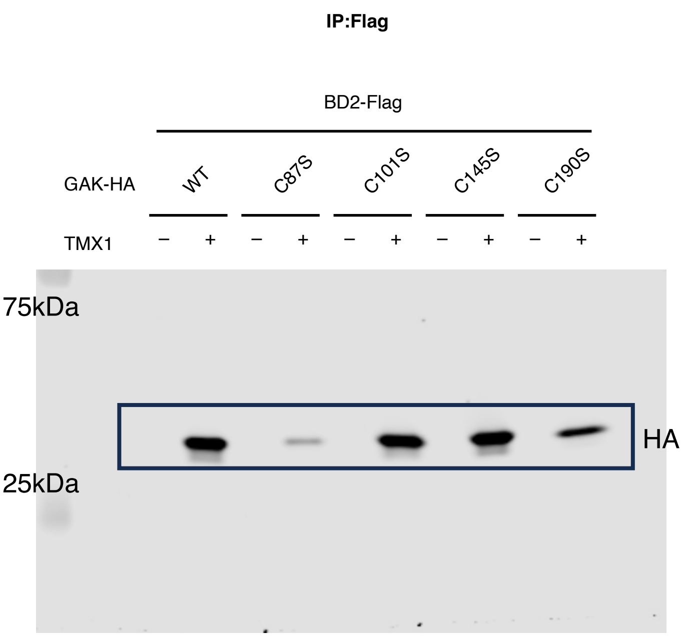

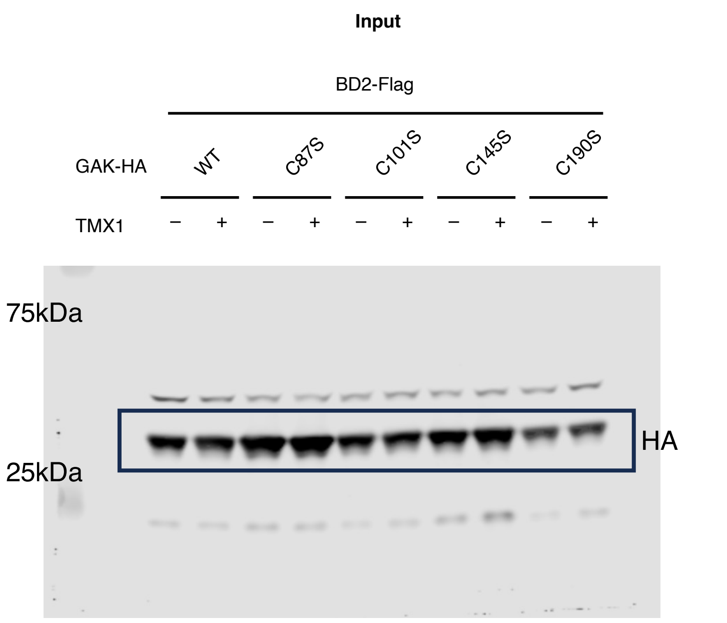

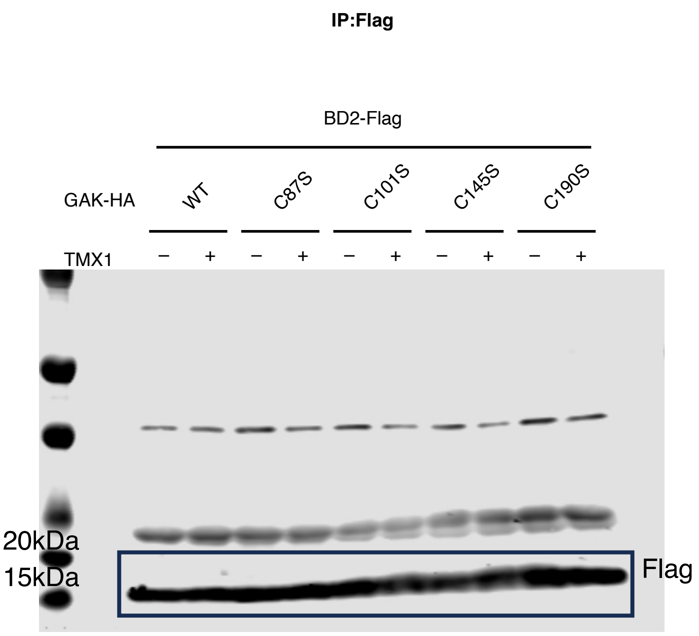

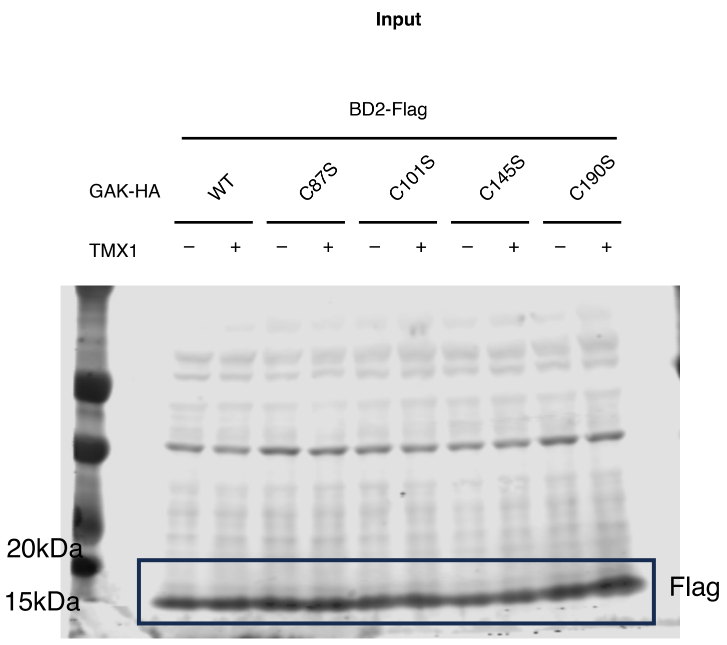

Related to Fig. 6c
IP blots were run on gel 1; Input blots were run on gel 2

intact-esf_014
GAK + MMH2
Fig. 6b

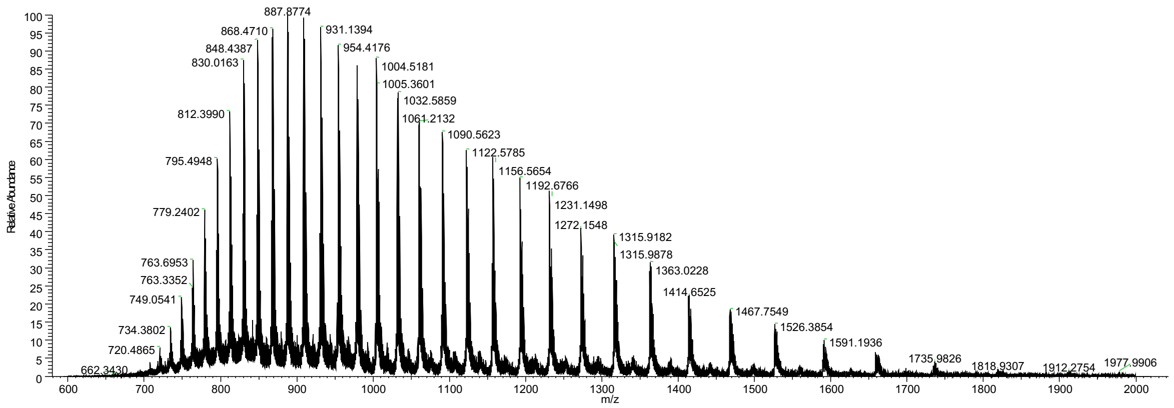

intact-esf_014
GAK + MMH2 + BD2
Fig. 6b

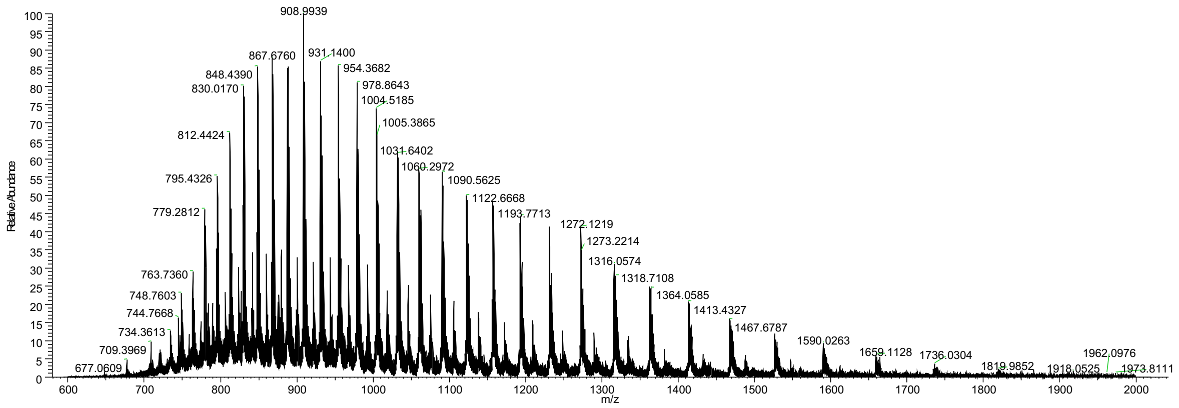

digest-esf_075
GAK + MMH2 + BD2
Fig. 6d

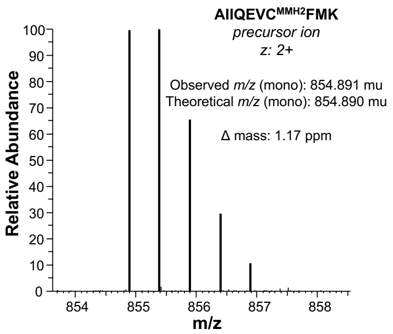

Fragment ion table

m/z theor. = fragment ion theoretical m/z
obs. ppm = delta mass of observed m/z from theoretical in ppm

#1	a^+ *m/z* theor.	a^+ obs. ppm	b^+ *m/z* theor.	b^+ obs. ppm	Seq.	y^+ *m/z* theor.	y^+ obs. ppm	y^+-C_4H_8 *m/z* theor.	y^+-C_4H_8 obs. ppm	y^{2+}-C_4H_8 *m/z* theor.	y^{2+}-C_4H_8 obs. ppm	#2
1	44.050		72.044		A							10
2	157.134	+0.06	185.129	-0.35	I	1637.735		1581.672		791.340		9
3	270.218		298.213	-1.15	I	1524.651		1468.588	+4.92	734.798	+3.72	8
4	398.276		426.271	-1.63	Q	1411.567		1355.504	+4.55	678.256		7
5	527.319	-1.19	555.314	-1.37	E	1283.508		1227.445	+4.54	614.226		6
6	626.387		654.382	-3.14	V	1154.465		1098.403	+5.41	549.705		5
7	1256.563		1284.557		C-MMH2	1055.397		999.334	+4.31	500.171		4
8	1403.631		1431.626		F	425.222	-1.38					3
9	1534.671		1562.666		M	278.153	-1.44					2
10					K	147.113	+1.83					1
