# Supplementary material for: Template-assisted covalent modification underlies activity of covalent molecular glues
Source: Nat Chem Biol. 2024 Jul 29;20(12):1640–9. doi: 10.1038/s41589-024-01668-4 (PMC11582070; doi:10.1038/s41589-024-01668-4)
Supplement: Supplementary file 20 — Uncropped western blot. [file 41589_2024_1668_MOESM20_ESM.pdf]

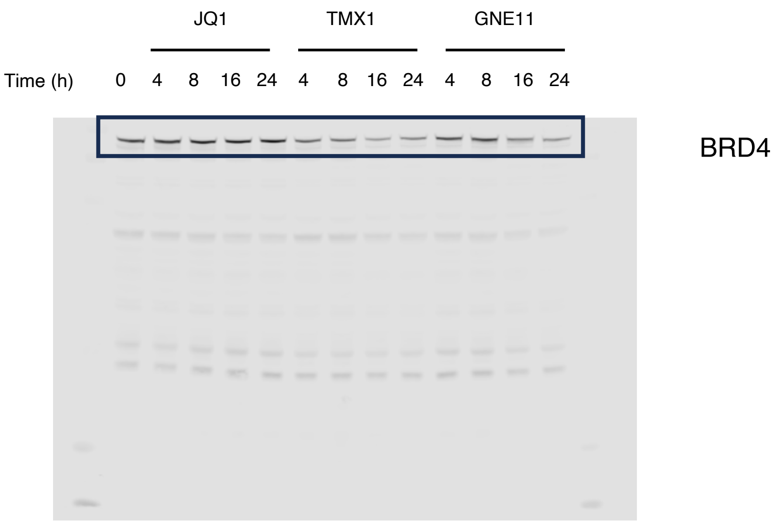


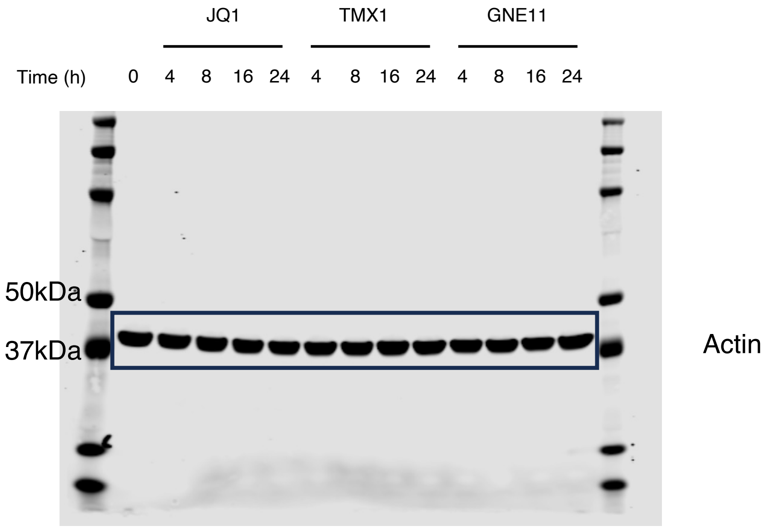


Related to Extended Data Fig. 1d
BRD4 and Actin blots were run on the same gel

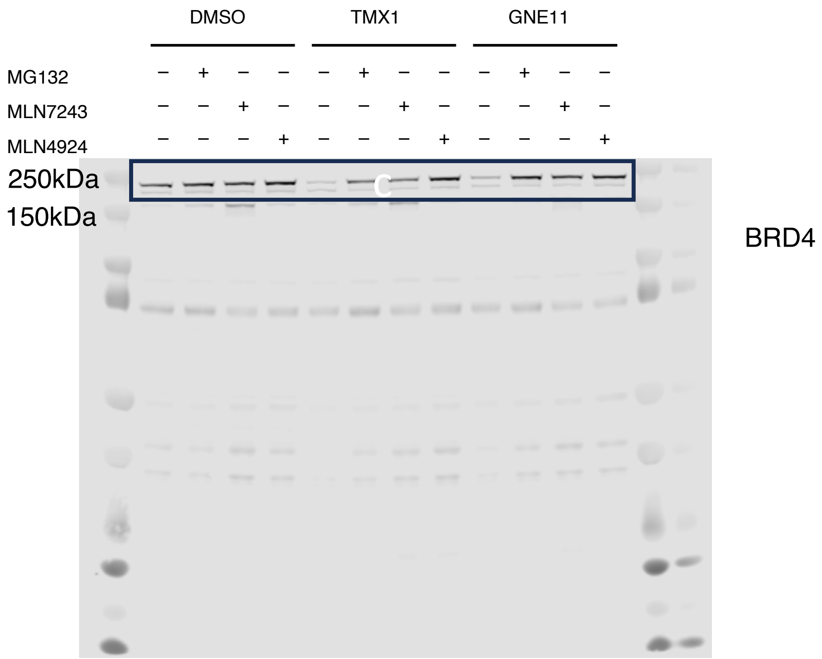


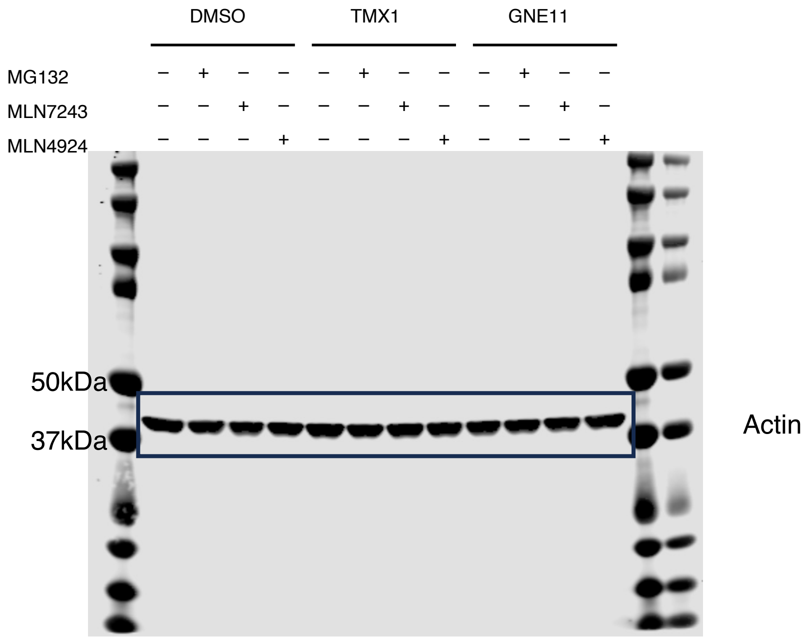


Related to Extended Data Fig. 1g
BRD4 and Actin blots were run on the same gel
