# Supplementary material for: Template-assisted covalent modification underlies activity of covalent molecular glues
Source: Nat Chem Biol. 2024 Jul 29;20(12):1640–9. doi: 10.1038/s41589-024-01668-4 (PMC11582070; doi:10.1038/s41589-024-01668-4)
Supplement: Supplementary file 23 — Uncropped western blot. [file 41589_2024_1668_MOESM23_ESM.pdf]

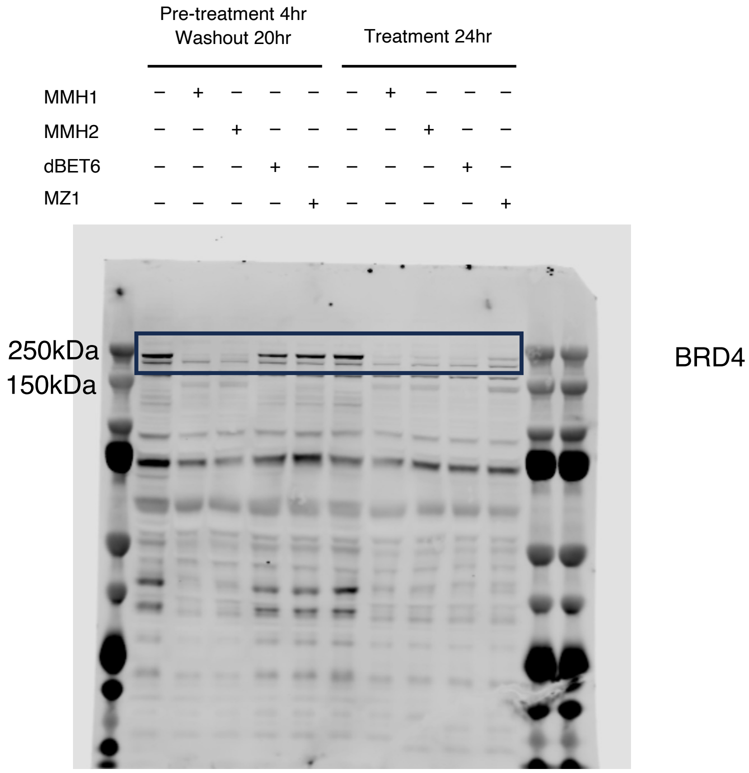

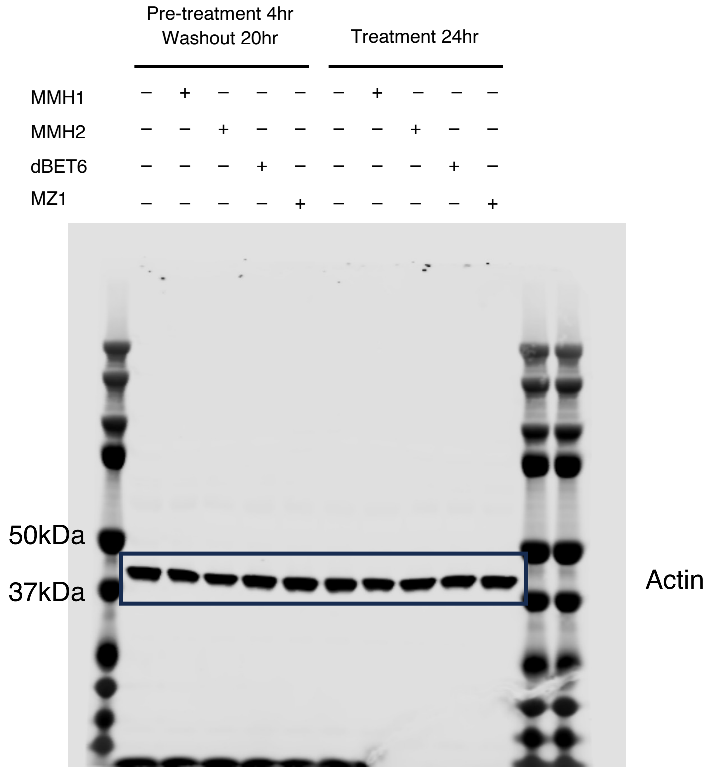

Related to Extended Data Fig. 3f

BRD4 and Actin blots were run on the same gel

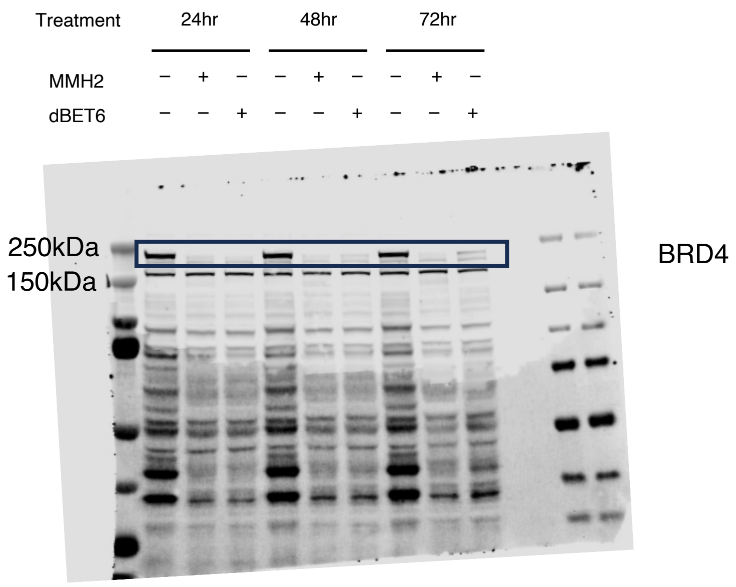

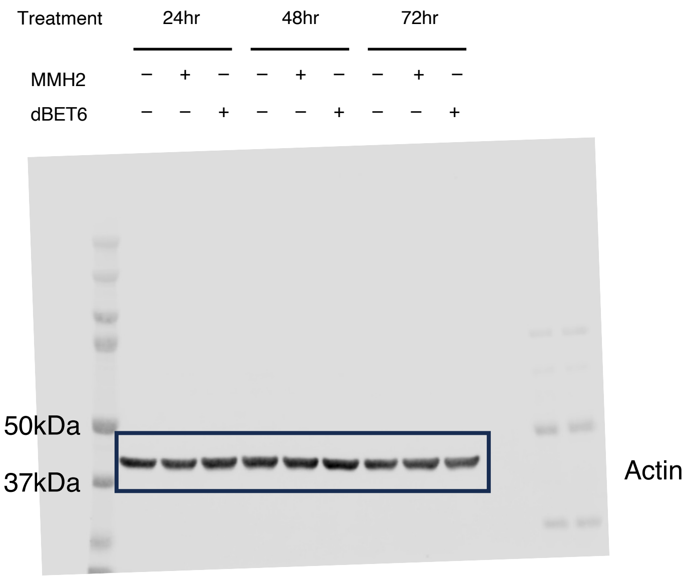

Related to Extended Data Fig. 3g
BRD4 and Actin blots were run on the same gel
