# Supplementary material for: Template-assisted covalent modification underlies activity of covalent molecular glues
Source: Nat Chem Biol. 2024 Jul 29;20(12):1640–9. doi: 10.1038/s41589-024-01668-4 (PMC11582070; doi:10.1038/s41589-024-01668-4)

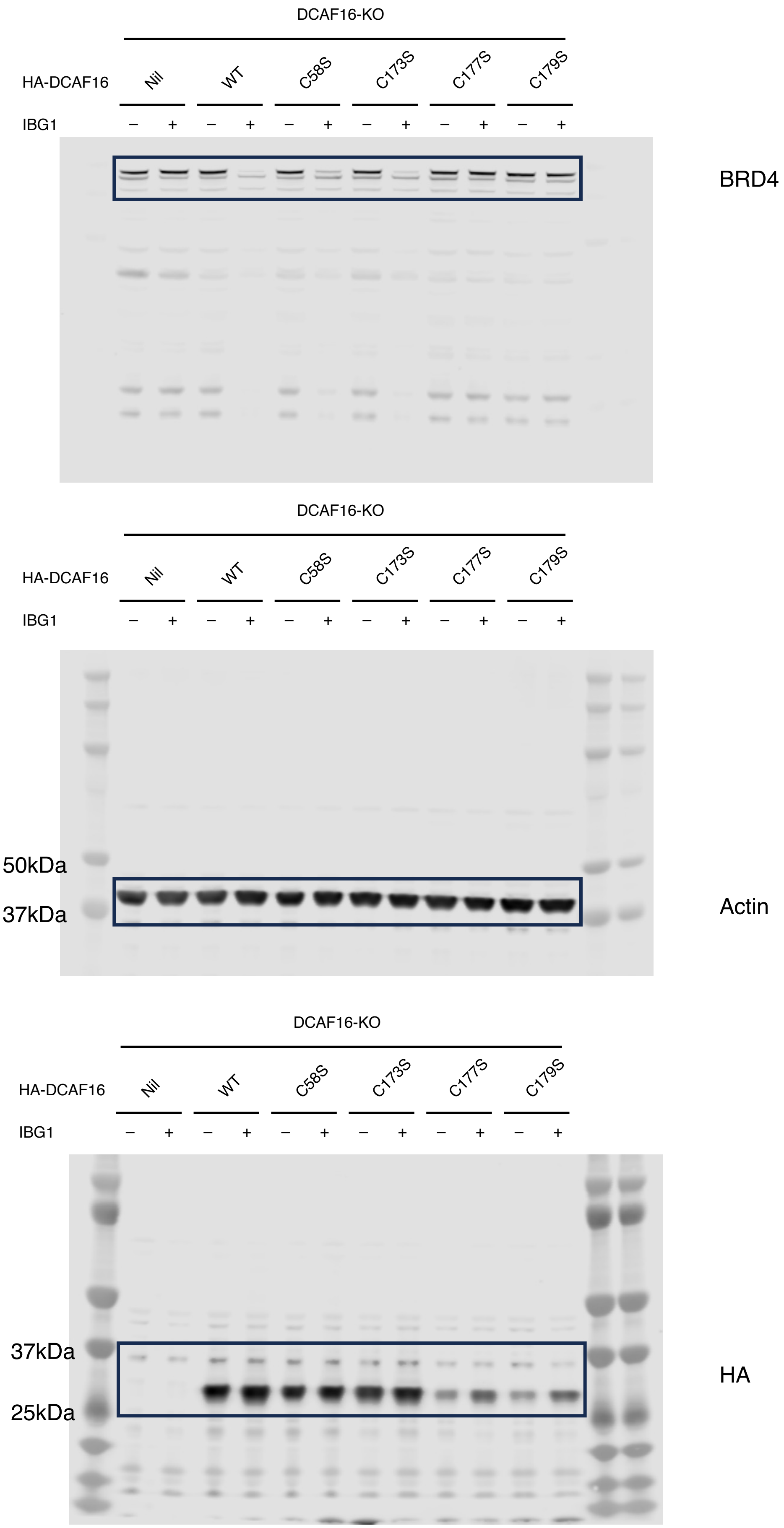

Related to Extended Data Fig. 8b

BRD4 and Actin blots were run on gel 1; HA blot was run on gel 2

intact-esf_001
DCAF16 + IBG1
Extended Data Fig. 8a

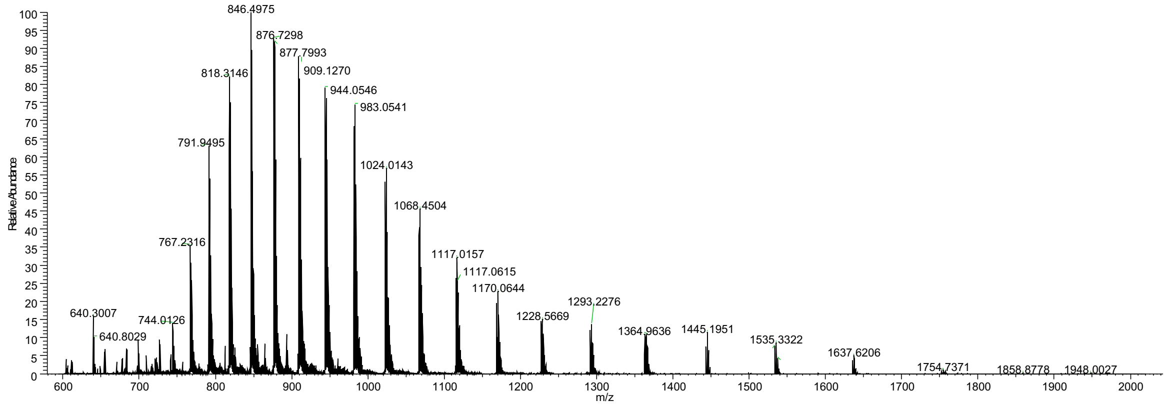

intact-esf_001
DCAF16 + IBG1 + BD2
Extended Data Fig. 8a

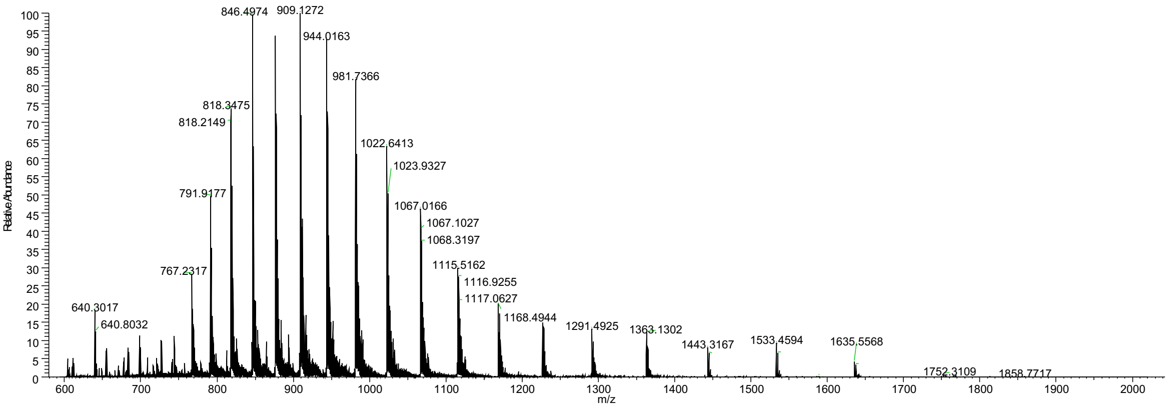

Supplement: Supplementary file 29 — Uncropped western blot and raw data for intact MS. [file 41589_2024_1668_MOESM29_ESM.pdf]
